# Hypothalamic TrkB.FL overexpression improves metabolic outcomes in the BTBR mouse model of autism

**DOI:** 10.1371/journal.pone.0282566

**Published:** 2023-03-09

**Authors:** Jacqueline M. Anderson, Amber A. Boardman, Rhiannon Bates, Xunchang Zou, Wei Huang, Lei Cao

**Affiliations:** 1 Department of Cancer Biology and Genetics, The Ohio State University, Columbus, OH, United States of America; 2 The Comprehensive Cancer Center, The Ohio State University, Columbus, OH, United States of America; Rutgers University, UNITED STATES

## Abstract

BTBR *T*+ *Itpr3tf*/J (BTBR) mice are used as a model of autism spectrum disorder (ASD), displaying similar behavioral and physiological deficits observed in patients with ASD. Our recent study found that implementation of an enriched environment (EE) in BTBR mice improved metabolic and behavioral outcomes. Brain-derived neurotrophic factor (*Bdnf*) and its receptor tropomyosin kinase receptor B (*Ntrk2*) were upregulated in the hypothalamus, hippocampus, and amygdala by implementing EE in BTBR mice, suggesting that BDNF-TrkB signaling plays a role in the EE-BTBR phenotype. Here, we used an adeno-associated virus (AAV) vector to overexpress the TrkB full-length (TrkB.FL) BDNF receptor in the BTBR mouse hypothalamus in order to assess whether hypothalamic BDNF-TrkB signaling is responsible for the improved metabolic and behavioral phenotypes associated with EE. Normal chow diet (NCD)-fed and high fat diet (HFD)-fed BTBR mice were randomized to receive either bilateral injections of AAV-TrkB.FL or AAV-YFP as control, and were subjected to metabolic and behavioral assessments up to 24 weeks post-injection. Both NCD and HFD TrkB.FL overexpressing mice displayed improved metabolic outcomes, characterized as reduced percent weight gain and increased energy expenditure. NCD TrkB.FL mice showed improved glycemic control, reduced adiposity, and increased lean mass. In NCD mice, TrkB.FL overexpression altered the ratio of TrkB.FL/TrkB.T1 protein expression and increased phosphorylation of PLCγ in the hypothalamus. TrkB.FL overexpression also upregulated expression of hypothalamic genes involved in energy regulation and altered expression of genes involved in thermogenesis, lipolysis, and energy expenditure in white adipose tissue and brown adipose tissue. In HFD mice, TrkB.FL overexpression increased phosphorylation of PLCγ. TrkB.FL overexpression in the hypothalamus did not improve behavioral deficits in either NCD or HFD mice. Together, these results suggest that enhancing hypothalamic TrkB.FL signaling improves metabolic health in BTBR mice.

## Introduction

Autism spectrum disorder (ASD) is a complex neurodevelopmental disorder characterized by deficits in social communication and social interaction and by repetitive and restricted patterns of behaviors, interests, and activities [1]. As of 2016, the prevalence of ASD in the United States is 1 in 54 children, with males being four times more likely than females to be diagnosed [2]. ASD is a very heterogeneous disorder, as individuals display varied combinations and severity of symptoms and comorbidities [1]. This heterogeneity makes it difficult to elucidate the underlying etiologies of ASD, but research suggests it likely involves a combination of genetics and environment influencing the developing brain [3]. Considering the environmental influence on the etiology of ASD and the use of environmental and sensory based therapies to treat ASD [4–6], investigating the effects of environment in an ASD-like murine model can help to elucidate the mechanisms behind ASD and ASD interventions.

Past studies in our lab have extensively examined the effects of an enriched environment (EE) in a variety of mouse models of disease, including cancer and obesity. We have found that placing mice in an EE providing physical, social, and cognitive stimuli induces an anti-obesity, anti-cancer, and anxiolytic phenotype [7–10]. We have elucidated one mechanism behind these effects, termed the hypothalamic-sympathoneural-adipocyte (HSA) axis. Stimuli from the EE upregulate brain-derived neurotrophic factor (BDNF) expression in the hypothalamus, which elevates sympathetic tone preferentially to white adipose tissues (WAT) [7, 8]. As a result, the norepinephrine released from sympathetic nerve acts on β-adrenergic receptor on the adipocytes leading to profound adipose remodeling. These adipose phenotypic changes are in parallel but all driven by the HSA axis: decreased leptin expression and release contributing to an anti-tumor effect; increased levels of vascular endothelial growth factor (VEGF), which increases energy expenditure and leanness through inducing beige cells; increased PTEN expression contributing to the reduction of adipocyte size and the increase of lipolysis; increased interleukin 15 (IL-15) expression leading to induction of adipose resident natural killer (NK) cells. The HSA axis-driven adipose remodeling plays a critical role in mediating the anticancer and anti-obesity effects of EE [7, 8, 10–12].

Based on the beneficial effects we saw in models of obesity and cancer after EE intervention, we recently investigated the effects of EE in the BTBR *T*+ *Itpr3tf*/J (BTBR) murine model of ASD. We found that placing BTBR mice in an EE mitigated both metabolic and behavioral deficits. Enriched male BTBR mice displayed lower adiposity, increased lean mass, lower levels of circulating leptin, and improved glucose tolerance. Behavioral tests suggested that an EE decreased anxiety-like behavior and improved social affiliation. Gene expression of *Bdnf* was significantly upregulated, which was consistent with HSA axis activation. Gene expression of *Ntrk2*, encoding the BDNF receptor tropomyosin kinase receptor B (TrkB), was upregulated 3 to 6 folds in the hypothalamus, hippocampus, and amygdala of EE mice, an extent larger than *Bdnf* [13].

The BTBR mouse was originally bred for studies on insulin-resistance, diabetes-induced nephropathy and phenylketonuria [14]. BTBR mice have a genetic propensity to obesity and type II diabetes. They have a genetic variation that promotes insulin resistance and this results in severe diabetes when crossed with the ob mutation in the leptin gene (BTBR ob/ob). BTBR mice also have alleles that increase body weight and obesity as compared to C57Bl/6 mice [15]. BTBR mice have higher fasting insulin levels and are insulin resistant as compared to C57Bl/6 mice due to adipose tissue insulin resistance, but they retain hepatic insulin sensitivity [16]. Insulin stimulated glucose uptake in adipose tissue and muscle tissue are defective in BTBR mice. Several inflammation related genes are upregulated in the adipose tissue of BTBR mice, and mRNA levels of leptin in adipose tissue are higher in BTBR mice than in C57Bl/6 mice [17]. Proteomic and transcriptomic studies of the hippocampus and cortex have found aberrant expression of proteins and genes involved in neurodevelopment, connectivity maintenance and guidance, neurogenesis, and neuroprotection, as well as disruption of inter and intracellular signaling pathways. The altered expression of genes involved in neurodevelopment and connectivity leads to the unique neuroanatomy of BTBR mice, which includes agenesis of the corpus callosum and a reduction of the hippocampal commissure. In addition to abnormal gene/protein expression and neuroanatomy, studies have found that BTBR mice may display an excitatory/inhibitory neurotransmission imbalance. BTBR mice also show increased basal corticosterone levels, which may be due to dysfunctional regulation of the HPA axis. Additionally, BTBR mice display aberrant immune responses. Basal plasma levels of IgG, IgE, anti-brain antibodies (Abs), and proinflammatory cytokines are higher in BTBR mice than in B6 mice. Within the brain, there is a higher number of mast cells and an increased proportion of MHC class II-expressing microglia, which suggests ongoing neuroinflammation [14, 18].

Over a decade ago, researchers discovered that BTBR mice could be used as a model of ASD, as it displayed relevant key diagnostic symptoms of ASD—selectively reduced social approach, low reciprocal social interactions, impaired juvenile play, and repetitive behaviors [19]. Since this discovery, much research has focused on behavioral phenotype characterization and genetic profiling of the BTBR mouse [19, 20], but less is known about the neurobiological mechanisms underlying the phenotypes. Notably, one study found that feeding BTBR mice a high-fat diet exacerbates cognitive rigidity and social deficiency [21]. Children with ASD are often more likely to develop obesity than children with typical development [22, 23]. This highlights a need for understanding the relationship between metabolic and cognitive health in individuals with ASD.

Our previous cancer and obesity studies identified BDNF as the key brain mediator for improved metabolic and immunity outcomes following EE, and our EE-BTBR study found that *Bdnf* and *Ntrk2* were upregulated following EE. The actions of BDNF are mediated by two major TrkB isoforms—full length TrkB (TrkB.FL) and truncated TrkB (TrkB.T1) [24]. Therefore, we hypothesized that BDNF-TrkB signaling was integral to the phenotypic outcomes induced by EE in BTBR mice. The purpose of this current study was to investigate the role of hypothalamic TrkB signaling in metabolic and behavioral phenotypes for both a normal chow diet (NCD)-fed and a high-fat diet (HFD)-fed BTBR mouse model.

## Materials and methods

### Mice and diet

**Fig 1:**
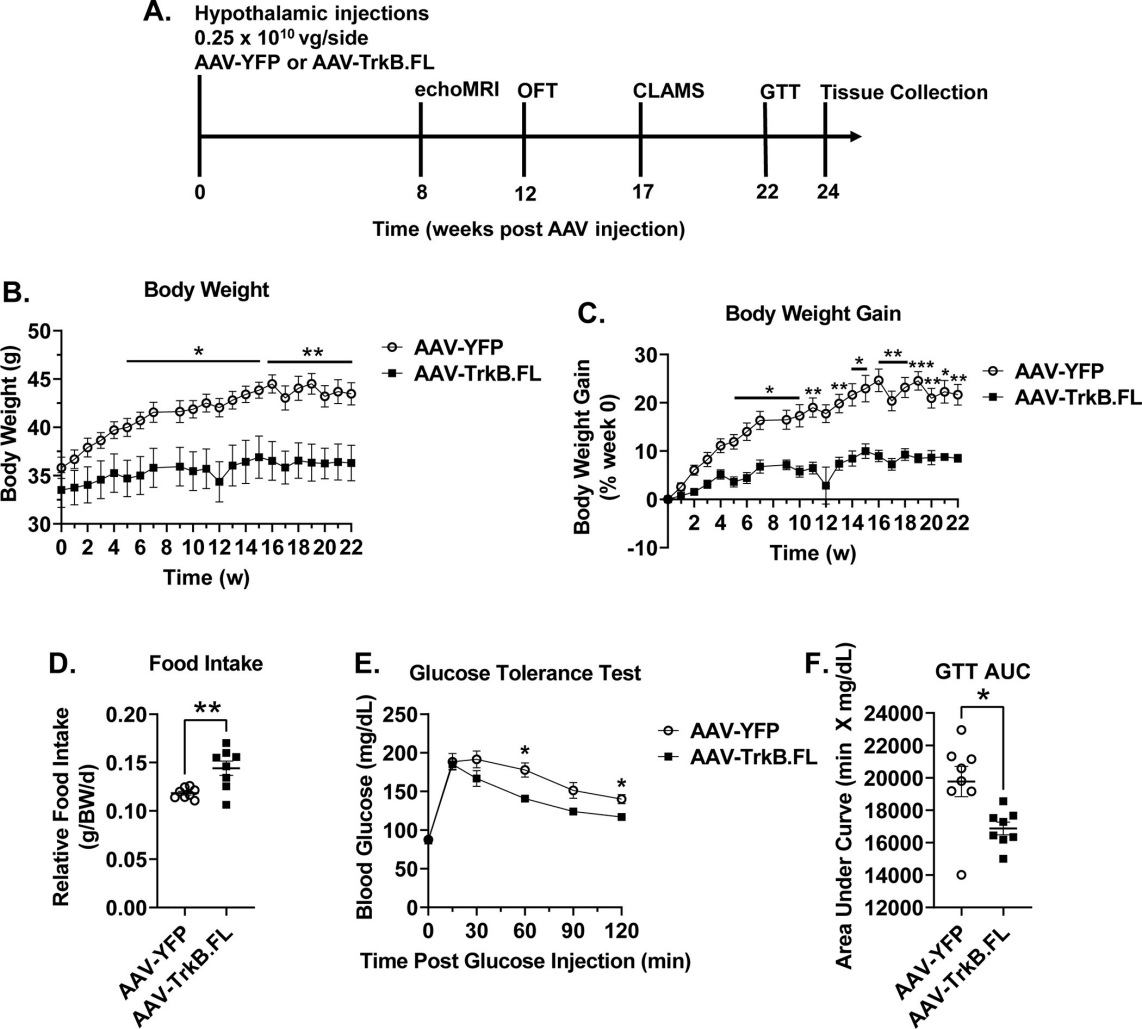
Hypothalamic AAV-TrkB.FL overexpression decreases body weight gain and improves glucose tolerance in NCD BTBR mice. (A) Experimental timeline. (B) Body weight. (C) Percent body weight gain. (D) Relative food intake. (E) Glucose tolerance test (GTT) (F) GTT area under the curve (AUC). Data are means ±SEM. AAV-YFP: n = 8, AAV-TrkB.FL: n = 8. * P<0.05, ** P<0.01, *** P<0.001.

Male BTBR *T*+ *Itpr3tf*/J (Jackson Laboratory #002282) mice were used to investigate the effects of adeno-associated virus (AAV) mediated hypothalamic TrkB.FL overexpression. Five cohorts of male mice were used—a long-term (24 wks), NCD (11% fat, caloric density 3.4 kcal/g, Teklad) fed cohort (n = 16); a long-term (23 wks), HFD (60% fat, caloric density 5.21 kcal/g, Research Diets, Inc. #D12492) fed cohort (n = 11); a short-term (4 wks) NCD fed cohort (n = 10); a short-term (4 wks) HFD fed cohort (n = 10), and a short-term C57BL/6 NCD fed cohort (n = 10). Ages of the mice were 15–17 weeks old (long-term NCD), 4–5 weeks old (long-term HFD), 4 weeks old (short-term NCD), 8–16 weeks old (short-term HFD), and 7–9 weeks old (short-term C57BL/6 NCD). For the HFD cohorts, mice were fed HFD for five weeks prior to AAV injections and maintained on the HFD for the duration of the studies. Weekly food consumption and body weights were recorded. One mouse from the short-term NCD cohort and one mouse from the short-term HFD cohort died during the study and were excluded from subsequent analysis. Experimental timelines for the long-term NCD and long-term HFD groups can be seen in Fig 1A and Fig 3A, respectively. All mice had *ad libitum* access to food and water. All mice were group housed (3–5 mice) in standard laboratory environment cages and housed in temperature (22–23°C) and humidity (30–70%) controlled rooms under a 12:12 light:dark cycle. All animal experiments were approved by The Ohio State University Institutional Animal Care and Use Committee.

### rAAV vector construction and packaging

The rAAV plasmid contains an expression cassette consisting of the CMV enhancer and chicken β-actin (CBA) promoter, woodchuck post-transcriptional regulatory element (WPRE) and bovine growth hormone poly-A flanked by AAV2 inverted terminal repeats. cDNA of TrkB full length (TrkB FL, NM-001025074) was amplified by using a mouse clone of Ntrk2 (ORIGENE, MR226130) as a template through polymerase chain reaction (PCR), with EcoRI flanking at each side. Primers for TrkB.FL amplification are as follows: forward, 5’-AATTAAGAATTCATGTCGCCCTGGCTGA-3’ and reverse, 5’-AATATAGAATTCTTA CAGATCCTCTTCTGAGA-3’. PCR product of TrkB-FL was then cloned into EcoRI site of the rAAV plasmid. The insert sequence was confirmed by sequencing at the OSU core facility. Amplified TrkB-FL retains a Myc tag at C-terminal before stop codon. rAAV plasmids containing TrkB-FL or yellow fluorescence protein (YFP) were packaged into serotype AAV1 vectors. The details of generation of rAAV were described previously [25].

### Stereotaxic surgery

Mice were randomized to receive either bilateral injections of AAV-YFP or AAV-TrkB.FL to the hypothalamus. Mice were anaesthetized with a single dose of ketamine/xylazine (100 and 20 mg kg^−1^; i.p.) and secured via ear bars and incisor bar on a Kopf stereotaxic frame. A mid-line incision was made through the scalp to reveal the skull and two small holes were drilled into the skull with a dental drill above the injection sites (-1.2 AP, ±0.5 ML, -6.2 DV, mm from bregma). rAAV vectors (2.5 × 10^9^ genomic particles per site) were injected bilaterally into the hypothalamus at a rate of 0.1 μl minute^−1^ using a 10 μl Hamilton syringe attached to Micro4 Micro Syringe Pump Controller (World Precision Instruments, Sarasota, FL). At the end of infusion, the syringe was slowly raised from the brain and the scalp was sutured. Animals were placed back into a clean cage and carefully monitored until recovery from anesthesia.

### Body composition

For the long-term, NCD group, echoMRI was utilized to measure body composition of fat, lean, free water, and total water masses in live mice without anesthesia at 8 weeks post-injection (wpi). Body composition analysis was performed with an echoMRI 3-in-1 Analyzer at the Small Animal Imaging Core of The Dorothy M. Davis Heart & Lung Research Institute, The Ohio State University.

### Energy expenditure

At 17 wpi (long-term NCD) and 12 wpi (long-term HFD), mice underwent indirect calorimetry using the Oxymax Comprehensive Lab Animal Monitoring System (CLAMS) (Columbus Instruments, Columbus, OH). Mice were singly housed with *ad libitum* access to food and water. Mice were acclimatized in the metabolic chambers for 18 hours, then behavior and physiological parameters (O_2_ consumption, CO_2_ production, respiratory exchange ratio, and physical activity) were recorded for 24 hours at room temperature.

### Glucose tolerance test

A glucose tolerance test (GTT) was conducted at 22 wpi (long-term NCD) and 11 wpi (long-term HFD). Mice were fasted for 16 hours overnight, then injected with glucose solution intraperitoneally (1.0 mg glucose/kg body weight). Blood was collected from the tail at baseline, 15, 30, 60, 90, and 120 minutes post glucose injection. Blood glucose concentrations were measured with a portable glucose meter (Bayer Contour Next).

### Behavioral methods

Methods for the behavioral assays can be viewed in S1 Text.

### Tissue harvest

Mice were sacrificed at 24 wpi (long-term NCD), 23 wpi (long-term HFD), and 4 wpi (short-term BTBR and C57BL/6 NCD and HFD BTBR). Mice were anesthetized by isoflurane and decapitated. Brown adipose tissue (BAT), gonadal WAT (gWAT), inguinal WAT (iWAT), and retroperitoneal WAT (rWAT), and liver were collected and weighed from both long-term groups. Gastrocnemius and pancreas were also dissected and weighed from the long-term NCD group. Hypothalamus was dissected from all groups. Tissues were flash-frozen on dry ice and stored at -80° C until further analysis.

### Quantitative real-time PCR

Total RNA was isolated from the hypothalamus, iWAT, gWAT, and BAT using the RNeasy Mini Kit plus RNase-free DNase treatment (Qiagen #74804). First-strand cDNA was generated using TaqMan Reverse Transcription Reagent (Applied Biosystems #N8080234). Quantitative real-time PCR was performed using Power SYBR Green PCR Master Mix (Applied Biosystems #A25742) on a StepOnePlus Real-Time PCR System (Applied Biosystems). Primer sequences can be viewed in S1 Table. Data were calibrated to endogenous control *Hprt1* for hypothalamus and *Actinb* for adipose tissues and the relative gene expression was quantified using the 2 ^-ΔΔCT^ method [26].

### Western blotting

Hypothalamus, iWAT, gWAT, and BAT were homogenized in ice-cold Pierce RIPA buffer containing 1× Roche PhosSTOP and Calbiochem protease inhibitor cocktail III, then spun at 13,000 rpm for 15 min. Tissue lysates were separated by gradient gel (4–20%, Mini-PROTEAN TGX, Bio-Rad) and transferred to a nitrocellulose membrane (Bio-Rad). Blots were incubated overnight at 4°C with primary antibodies listed in S2 Table. Blots were rinsed and incubated with HRP-conjugate secondary antibody (Bio-Rad). Chemiluminescence signal was detected and visualized by LI-COR Odyssey Fc imaging system (LI-COR Biotechnology). Quantification analysis was carried out with image studio software version 5.2 (LI-COR Biotechnology). Phosphorylated proteins were calibrated to their total protein levels and presented as ratio. TrkB.FL, TrkB.T1, Ras, and PTEN were normalized to reference proteins.

### Statistical analysis

Data are expressed as mean ± SEM. GraphPad Prism 7 software (GraphPad, La Jolla, CA) was used to analyze our data, using Student’s t-tests. *P* ≤0.05 was considered statistically significant. Data were tested for normality using the Shapiro-Wilk test. If data violated assumptions of normality, either log2 transforms or Mann-Whitney tests were performed, and analysis was repeated. Welch’s correction was performed for data that violated assumptions of homogeneity of variance. Power analyses were conducted post-hoc. An ANOVA mixed effects model was used to analyze all longitudinal data, and Bonferroni’s test was used for corrections post-hoc.

## Results

### Hypothalamic TrkB.FL overexpression improves metabolic outcomes in BTBR mice fed on normal chow diet

We firstly assessed effects of hypothalamic TrkB.FL overexpression in BTBR mice fed on NCD (Fig 1A). All outliers for all data have been included. Visualization to verify AAV injection location can be viewed in S1 Fig. AAV-TrkB.FL mice displayed reduced body weight gain (Fig 1B) and reduced percent body weight gain (Fig 1C) as compared to AAV-YFP mice. AAV-TrkB.FL mice consumed significantly more food relative to body weight than AAV-YFP mice (P = 0.01) (Fig 1D).

**Fig 2:**
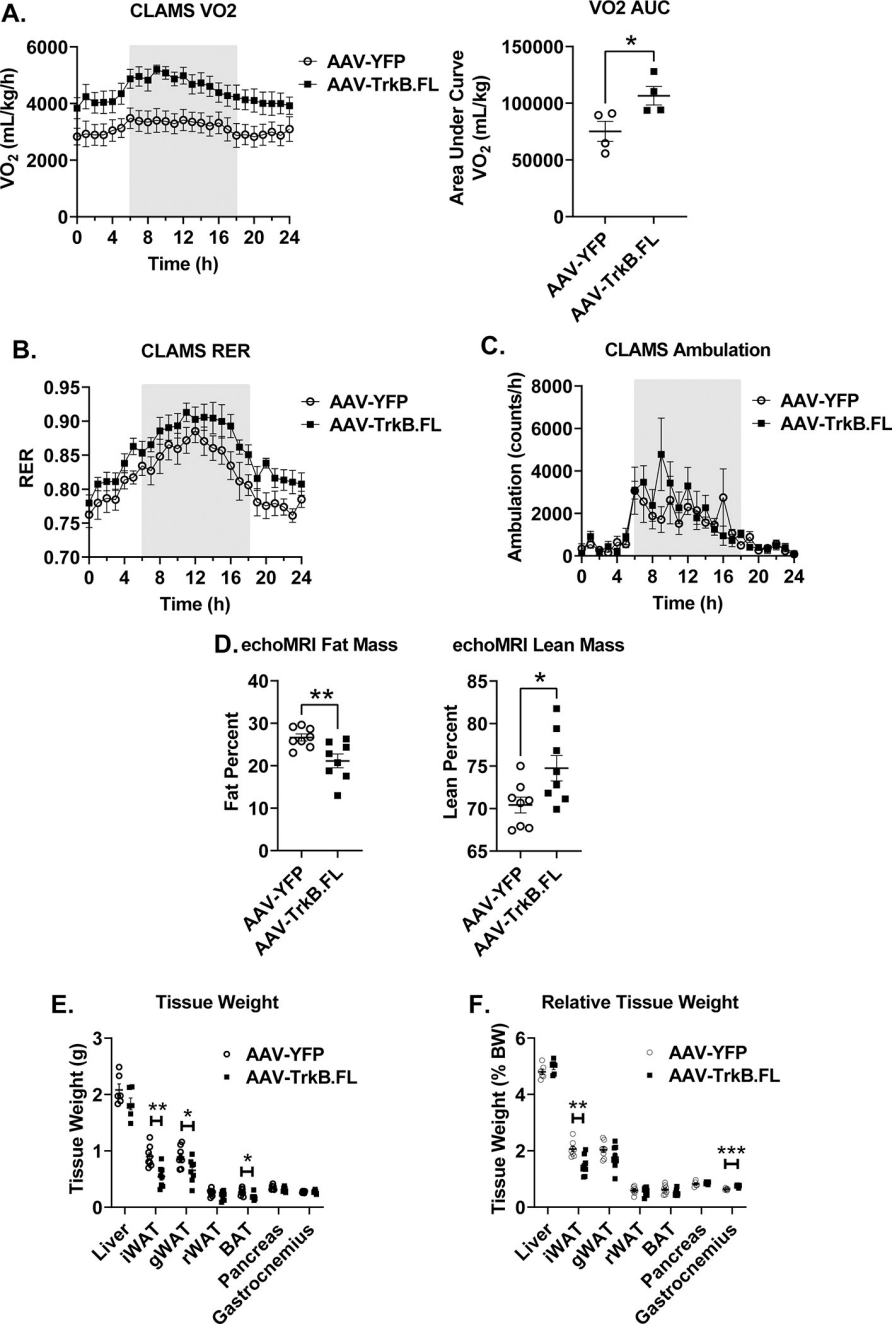
Hypothalamic TrkB.FL overexpression improves metabolic outcomes in NCD BTBR mice. (A) O2 consumption and AUC. (B) Respiratory exchange ratio (RER). (C) Ambulation. (D) Percent fat mass and percent lean mass, as measured by echoMRI. (E) Tissue weight (liver, inguinal white adipose tissue, gonadal white adipose tissue, retroperitoneal white adipose tissue, brown adipose tissue, pancreas, gastrocnemius) (F) Relative tissue weight, normalized to body weight. Data are means ±SEM. AAV-YFP: n = 8, AAV-TrkB.FL: n = 8. * P<0.05, ** P<0.01, *** P<0.001.

At 22 wpi, mice were subjected to a GTT (Fig 1E). AAV-TrkB.FL mice displayed improved glycemic control compared to AAV-YFP mice as measured by an ANOVA mixed effects model (P = 0.0195) and area under the curve calculation (P = 0.0126) (Fig 1F). At 17 wpi, mice underwent indirect calorimetry using CLAMS. AAV-TrkB.FL animals showed significantly higher oxygen consumption compared to their AAV-YFP counterparts, measured by an ANOVA mixed effects model (P = 0.0418) and area under the curve calculation (P = 0.0403) (Fig 2A). The respiratory exchange ratio (RER) and physical activity was not significantly different between AAV-TrkB.FL and AAV-YFP animals (Fig 2B and 2C). An *in vivo* echoMRI was performed at 8 wpi to assess body composition. AAV-TrkB.FL mice displayed significantly decreased percent fat mass (P = 0.0086) and significantly increased percent lean mass as compared to AAV-YFP mice (P = 0.028) (Fig 2D).

At sacrifice, tissue weights of iWAT, gWAT and BAT were significantly reduced (P = 0.0016, P = 0.027, P = 0.014) in the long-term NCD AAV-TrkB.FL mice as compared to AAV-YFP mice (Fig 2E). When normalized to body weight, iWAT mass was significantly reduced (P = 0.0022) and gastrocnemius mass was significantly increased (P = 0.0003) in the AAV-TrkB.FL animals (Fig 2F).

### Hypothalamic gene transfer of TrkB.FL improves metabolic outcomes in obese BTBR mice

**Fig 3:**
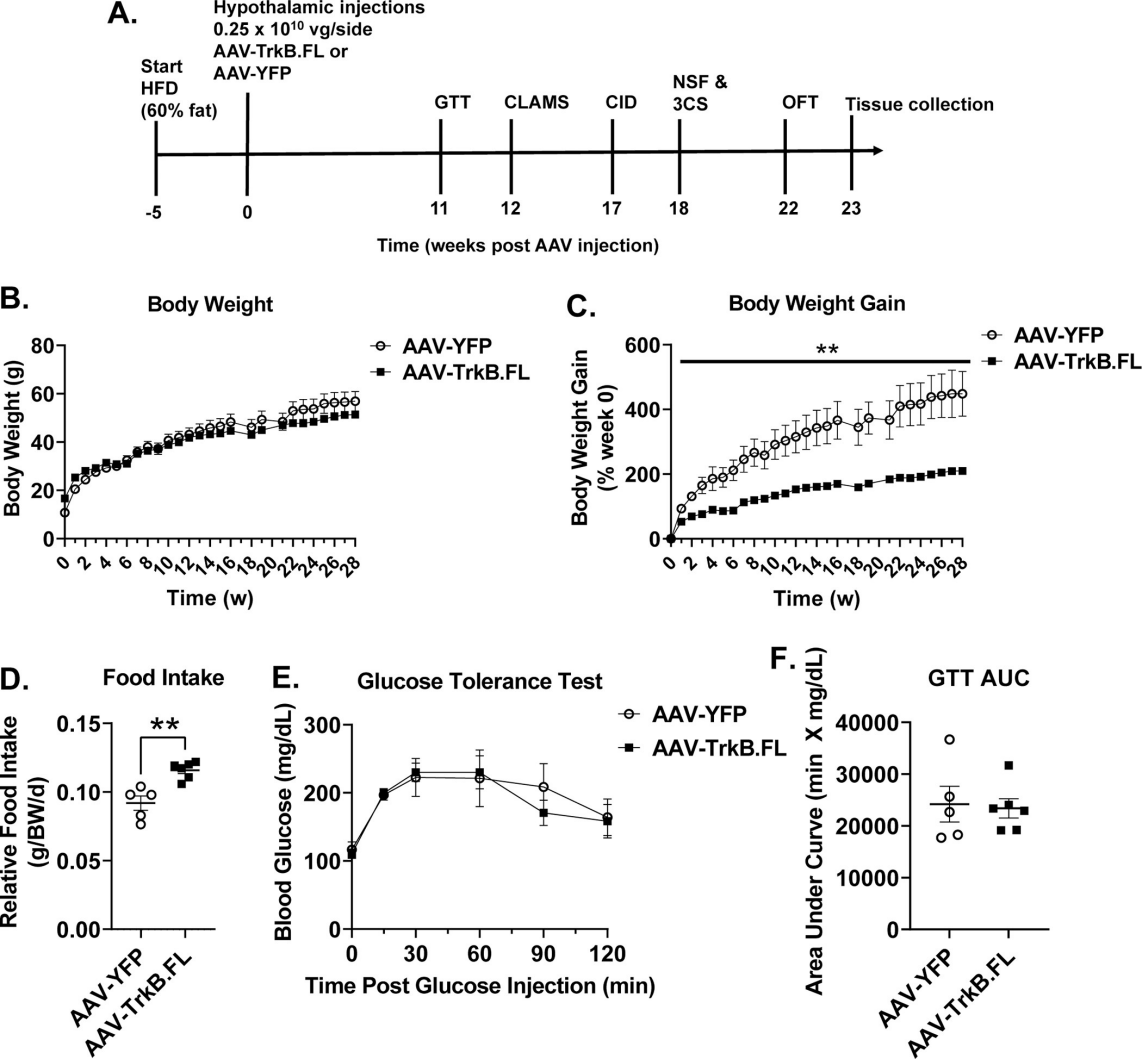
Hypothalamic AAV-TrkB.FL overexpression decreases body weight gain in HFD BTBR mice. (A) Experimental timeline. (B) Body weight. (C) Percent body weight gain. (D) Relative food intake. (E) Glucose tolerance test (GTT) (F) GTT area under the curve (AUC). Data are means ±SEM. AAV-YFP: n = 5, AAV-TrkB.FL: n = 6. * P<0.05, ** P<0.01.

**Fig 4:**
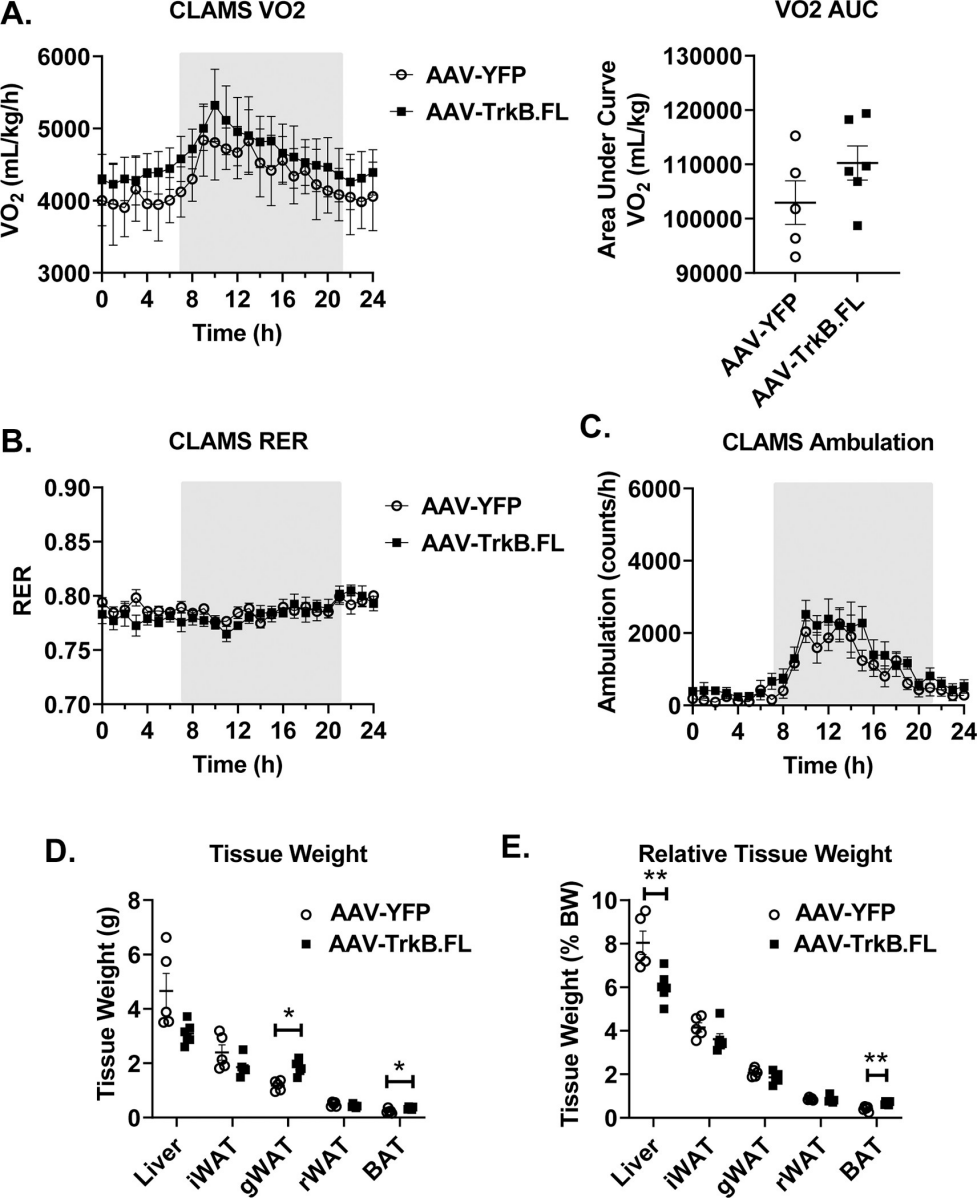
Metabolic outcomes of hypothalamic AAV-TrkB.FL overexpression in HFD BTBR mice. (A) O2 consumption and AUC. (B) Respiratory exchange ratio (RER). (C) Ambulation. (D) Tissue weight (liver, inguinal white adipose tissue, gonadal white adipose tissue, retroperitoneal white adipose tissue, brown adipose tissue) (E) Relative tissue weight, normalized to body weight. Data are means ±SEM. AAV-YFP: n = 5, AAV-TrkB.FL: n = 6. * P<0.05, ** P<0.01.

We next examined whether hypothalamic gene transfer of TrkB.FL alters obesity and associated metabolic dysfunction in obese BTBR mice. AAV-TrkB.FL and AAV-YFP mice had a significant weight difference at the start of HFD feeding and did not display significant differences in total weight gain over the course of the experiment (Fig 3B). Due to the significant difference in body mass at the beginning of the experiment, we calculated percent body weight gain and found a significantly reduced percent weight gain in the AAV-TrkB.FL injected mice as compared to the AAV-YFP injected mice (Fig 3C). AAV-TrkB.FL mice consumed significantly more food relative to body weight than AAV-YFP mice (P = 0.0018) (Fig 3D). At 11 wpi, mice were subjected to a GTT (Fig 3E) and showed no significant difference (Fig 3F). At 12 wpi, AAV-TrkB.FL animals showed no significant differences in VO2, RER, or ambulation as compared to their AAV-YFP counterparts (Fig 4A–4C).

At termination of the experiment 23 wpi, relative liver weight was significantly reduced (P = 0.0067) (Fig 4D) while relative BAT weight was significantly increased (P = 0.0011) in AAV-TrkB.FL mice as compared to AAV-YFP mice in the long-term HFD experiment (Fig 4E).

### Hypothalamic TrkB.FL overexpression does not improve behavioral deficits in BTBR mice

In our previous study investigating the effects of enriched environment on BTBR metabolism and behavior, we found significant differences between groups for the open field test and three chambered sociability, so we repeated those tests in this study. To measure anxiety-like behavior and locomotion, an open field test was performed at 12 wpi (long-term NCD) and 22 wpi (long-term HFD). Total distance traveled, distance traveled in the periphery/distance traveled total ratio, and distance traveled in the center/distance traveled total ratio were measured. There was no significant difference between AAV-TrkB.FL and AAV-YFP mice for any measures, in both the long-term NCD (S2 Fig) and the long-term HFD group (S3A–S3C Fig).

Another test to measure anxiety-like behavior is the novelty suppressed feeding test, which was performed at 18 wpi for the long-term HFD group. Mice were fasted overnight and then placed in a new cage with a piece of chow. Latency to feed and food consumed were measured. No significant differences were found between AAV-TrkB.FL and AAV-YFP mice for either measure (S3D and S3E Fig).

In addition, long-term HFD mice were subjected to the third anxiety-like behavioral test, cold induced defecation test, at 18 wpi. Cold temperatures can induce stress in mice and stress increases defecation [27, 28]. We expected that an increase in number of fecal boli would reflect an increase in anxiety-like behavior. No significant differences were found between AAV-TrkB.FL and AAV-YFP mice (S3F Fig).

For the long-term HFD experiment, the three-chambered sociability (3CS) test was conducted at 18 wpi. For the first phase, the time spent in the mouse-filled chamber, time spent in the center, and time spent in the empty chamber was recorded. The social affiliation index was calculated by taking the ratio of the time spent in the mouse chamber over the time spent in the empty chamber. No significant differences were found between AAV-TrkB.FL mice and AAV-YFP mice for any of these measures. For the second phase, the time spent in each chamber was recorded. The social novelty index was calculated by taking the ratio of time spent in the novel mouse chamber over the familiar mouse chamber. Again, there were no significant differences between AAV-TrkB.FL and AAV-YFP mice for any measures (S4 Fig). In ASD sometimes social deficits appear not as a lack of overall sociability but as inappropriate or indiscriminate approaches to strangers. The two parts of the three chamber sociability test allow us to test two distinct aspects of social behavior- 1) overall social approach and 2) social memory and novelty. The first part of the three chamber sociability test measured overall sociability, examining whether the mouse preferred an inanimate object to a mouse. We utilized the second part of the three chamber sociability test to measure social novelty preference. Generally, a C57Bl/6J mouse, when presented with both a familiar mouse and a novel mouse, will spend more time investigating the novel mouse due to the highly social nature of mice. In models of autism the mice spend less or equal time investigating the novel mouse, which is abnormal as wild-type mice have a preference for social novelty. This behavior could be analogous to social behavior in humans with ASD, where they may prefer to spend time with familiar people over new people or they approach strangers indiscriminately [29–31].

### Hypothalamic TrkB.FL overexpression changes hypothalamic gene expression in NCD BTBR mice

**Fig 5:**
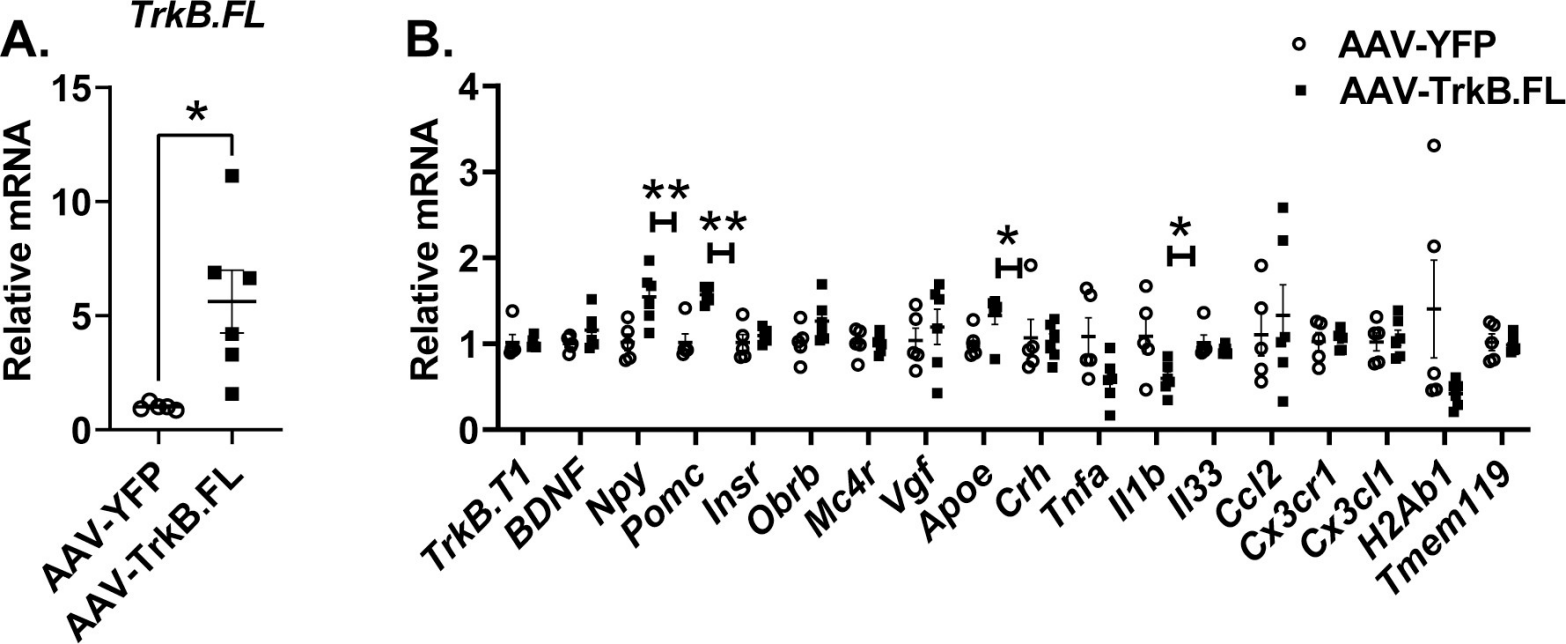
Hypothalamic AAV-TrkB.FL treatment alters hypothalamic gene expression in NCD BTBR mice. (A) Relative mRNA expression of *TrkB*.*FL*. (B) Relative mRNA expression. Data are means ±SEM. AAV-YFP: n = 8, AAV-TrkB.FL: n = 8. * P<0.05, ** P<0.01.

Real-time quantitative RT-PCR was used to profile hypothalamic gene expression in the long-term NCD experiment (Fig 5). AAV-TrkB.FL injected animals showed approximately 5-fold higher expression of Trk.FL compared to control mice (P = 0.0202) (Fig 5A) while the isoform TrkB.T1 was not different (Fig 5B). Genes involved in energy homeostasis and BDNF signaling were examined including *Bdnf*, *Mc4r* (encoding melanocortin-4 receptor), *Vgf* (encoding nerve growth factor inducible), *Insr* (encoding insulin receptor), *Obrb* (encoding long form leptin receptor), *Crh* (encoding corticotropin-releasing hormone), *Npy* (encoding neuropeptide Y), and *Pomc* (encoding proopiomelanocortin). Interestingly, both anorexigenic *Pomc* and orexigenic *Npy* were significantly upregulated in AAV-TrkB.FL injected mice (P = 0.0043, P = 0.0092) (Fig 5B).

Neuroinflammation is implicated in the aberrant behaviors of BTBR mice [32]. Accordingly, we examined a panel of immunomodulatory genes and microglial markers in the long-term NCD experiment. Overexpressing TrkB.FL significantly downregulated the expression of *Il1b* (encoding interleukin-1β) (P = 0.037) while upregulated *Apoe* (encoding apolipoprotein) (P = 0.039). It is reported that BTBR mice show an increased proportion of MHC class II (encoded by *H2ab1*)-expressing microglia compared to sociable strain C57BL/6 [32]. Hypothalamic *H2ab1* and *Tnfa* (encoding tumor factor-α) expression showed a trend of downregulation in the AAV-TrkB.FL mice although not reaching significance (P = 0.089, P = 0.052). No changes were observed among *Il33* (encoding interleukin-33), *Ccl2* (encoding C-C motif chemokine ligand 2), or *Cx3cr1* (encoding C-X3-C motif chemokine receptor 1) (Fig 5B).

**Fig 6:**
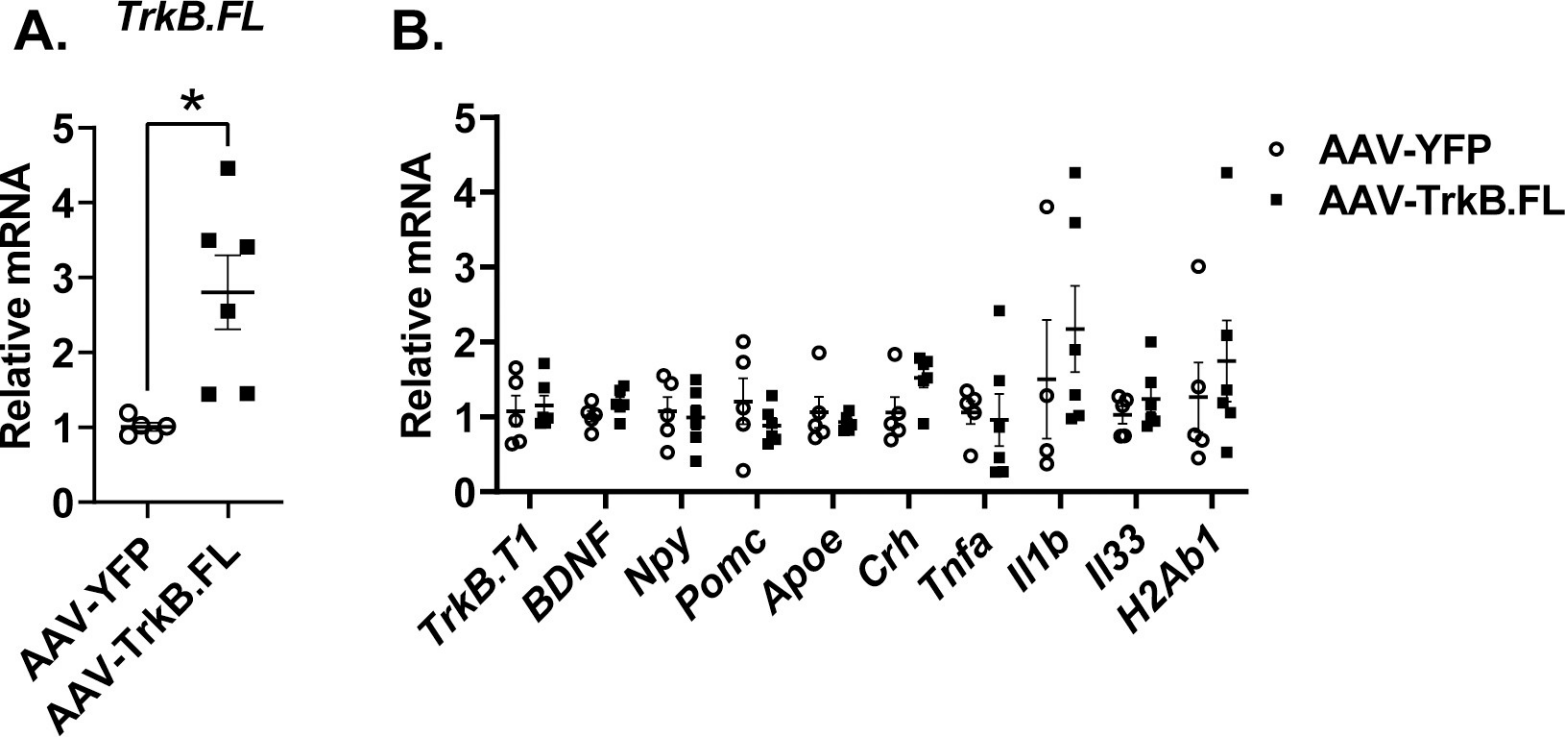
Hypothalamic AAV-TrkB.FL treatment alters hypothalamic gene expression in HFD BTBR mice. (A) Relative mRNA expression of *TrkB*.*FL*. (B) Relative mRNA expression. Data are means ±SEM. AAV-YFP: n = 5, AAV-TrkB.FL: n = 6. * P<0.01.

We profiled hypothalamic gene expression in the long-term HFD experiment including BDNF-TrkB relevant genes and the genes whose expression was altered by TrkB.FL overexpression in the long-term NCD experiment (Fig 6). TrkB.FL overexpression was confirmed in the HFD AAV-TrkB.FL injected mice but to a milder extent relative to the NCD experiment (P = 0.015) (Fig 6A, Fig 5A). There were no other significant differences in the long-term HFD group (Fig 6B).

### Hypothalamic TrkB.FL overexpression alters adipose gene expression in NCD BTBR mice

**Fig 7:**
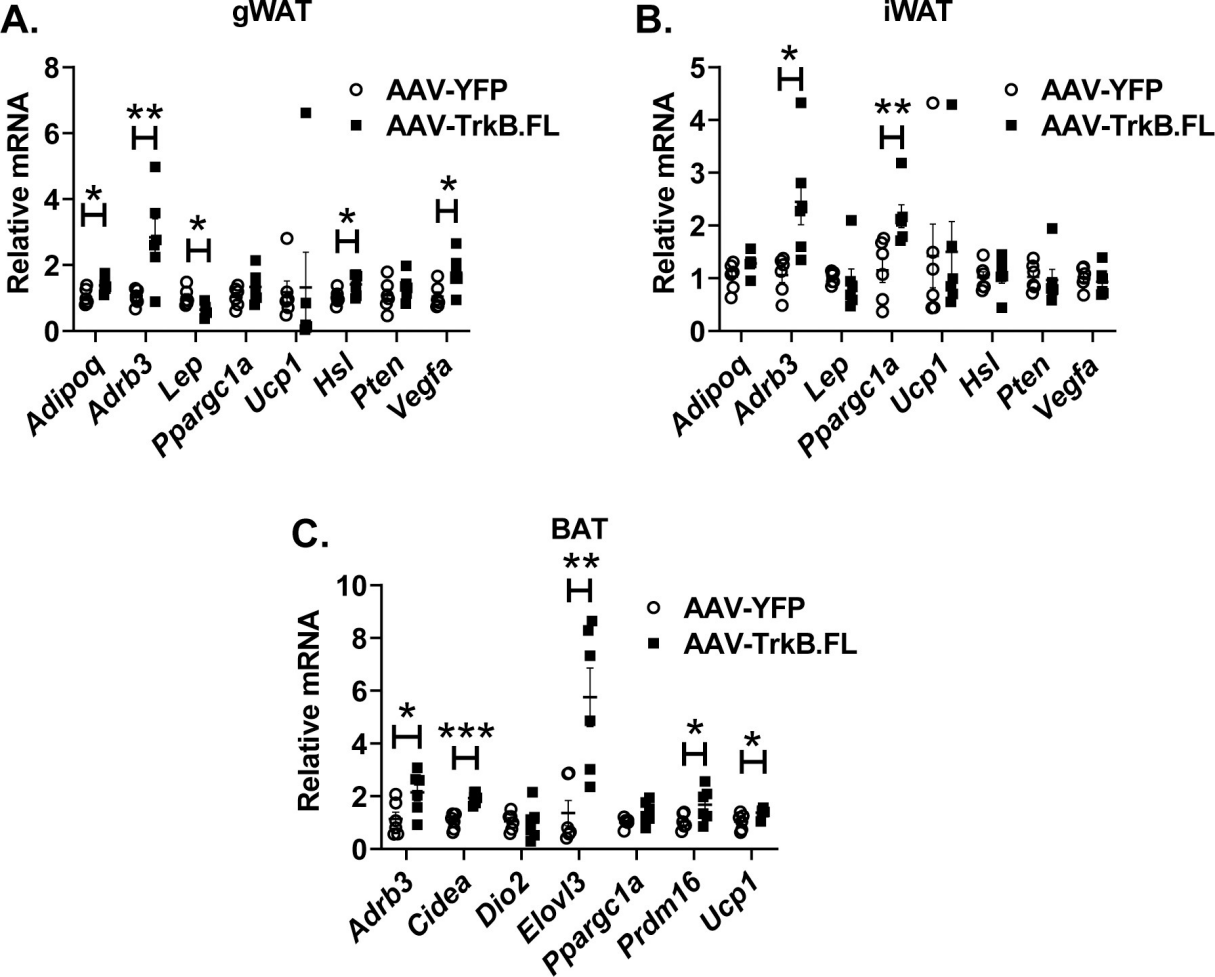
Hypothalamic TrkB.FL overexpression regulates adipose gene expression in NCD BTBR mice. (A) Relative mRNA expression in gWAT. (B) Relative mRNA expression in iWAT. (C) Relative mRNA expression in BAT. Data are means ±SEM. AAV-YFP: n = 8, AAV-TrkB.FL: n = 8. * P<0.05, ** P<0.01, *** P<0.001.

Our previous studies have found that hypothalamic BDNF overexpression results in sympathoneural activation of adipose tissue and revealed adipose depot-dependent gene expression signatures consisting of the genes involved in thermogenesis, lipolysis, and energy metabolism [7, 8, 10–12]. Thus, we profiled gWAT, iWAT, and BAT gene expression in the long-term NCD experiment (Fig 7) targeting the gene signatures associated with hypothalamic BDNF overexpression. In gWAT, overexpression of TrkB.FL significantly upregulated gene expression of *Adipoq* (encoding adiponectin, C1Q and collagen domain containing, P = 0.0218), *Hsl* (encoding hormone sensitive lipase, P = 0.0332), *Vegfa* (encoding vascular endothelial growth factor A, P = 0.0249) and downregulated *Lep* (encoding leptin, P = 0.0178) (Fig 7A). Hypothalamic overexpressing TrKB.FL significantly upregulated *Adrb3* (encoding adrenoceptor β3) expression in all adipose depots examined, gWAT (P = 0.0218), iWAT (P = 0.0226), and BAT (P = 0.0359). *Ppargc1α* (encoding PPARG coactivator 1 alpha) was upregulated in iWAT of TrkB.FL mice (P = 0.0095) (Fig 7B). In BAT, a cluster of thermogenic genes including *Cidea* (encoding cell death inducing DFFA like effector a, P = 0.0001), *Elovl3* (encoding ELOVL fatty acid elongase 3, P = 0.0046), *Prdm16* (encoding PR/SET domain 16, P = 0.0483), and *Ucp1* (encoding uncoupling protein 1, P = 0.0396) were significantly upregulated in TrkB.FL injected mice (Fig 7C).

### Hypothalamic TrkB.FL gene transfer affects signaling mediators downstream of TrkB in BTBR mice

The TrkB family includes at least three well-characterized TrkB isoforms through alternative RNA splicing. TrkB.FL is a receptor tyrosine kinase containing an extracellular ligand-binding domain, a single transmembrane domain, and a typical intracellular domain for tyrosine kinases. Two other isoforms (TrkB.T1 and TrkB.T2) are truncated for their intracellular kinase binding domain [33]. TrkB.FL expressed by our rAAV vector will not generate the truncated isoforms.

**Fig 8:**
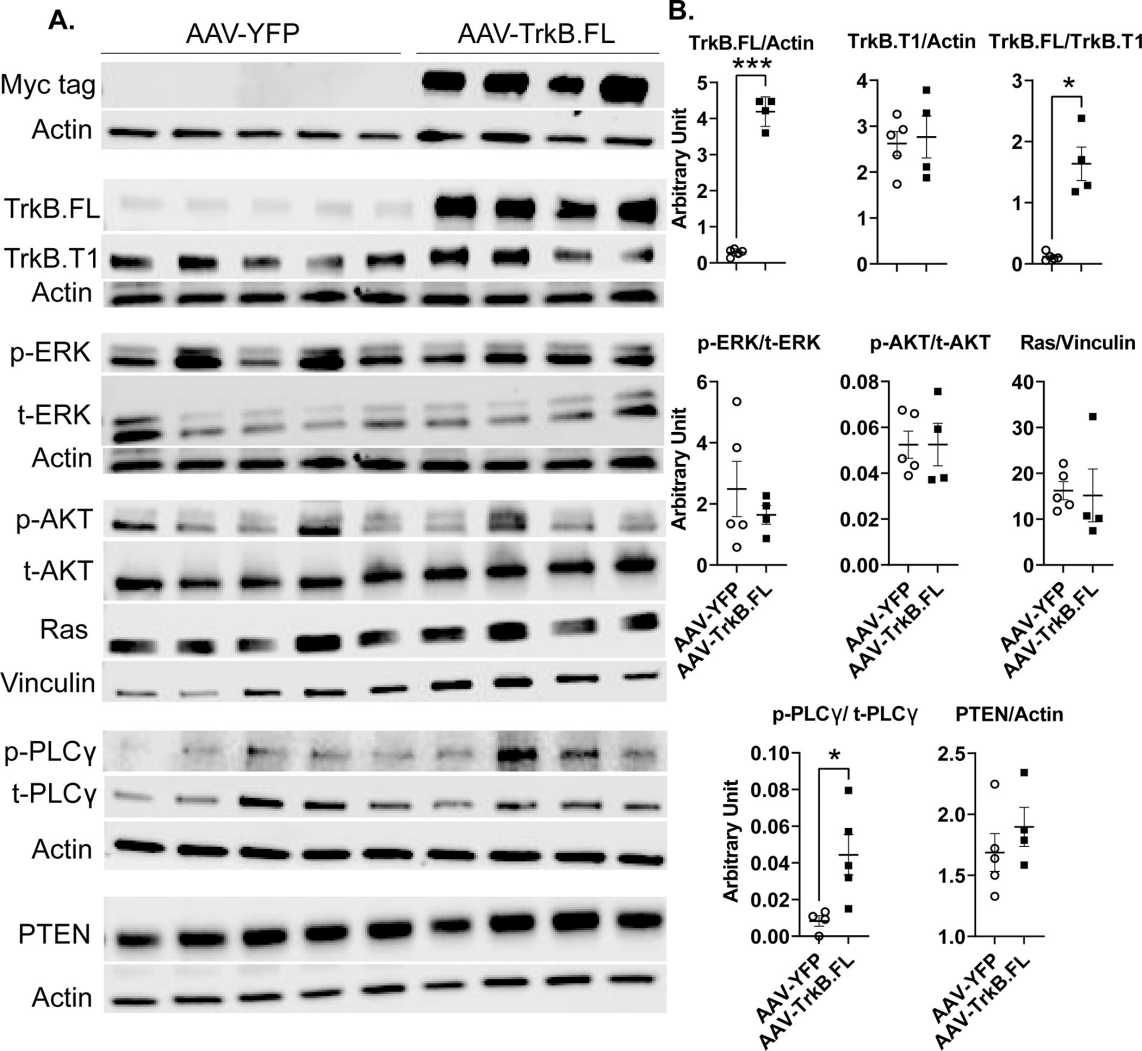
Hypothalamic AAV-TrkB.FL overexpression affects signaling mediators downstream of TrkB in NCD BTBR mice. (A) Western blotting of right lobe of the hypothalamus. (B) Quantification of (A). Data are means ±SEM. AAV-YFP: n = 5, AAV-TrkB.FL: n = 4. * P<0.05, *** P<0.001.

Three canonical pathways are activated following TrkB receptor activation—MAPK/ERK, PI3K/AKT, and PLCγ [34]. Because we used all hypothalamic dissections from the long-term NCD experiment for qRT-PCR, we were unable to examine the signaling molecules. Hence, we carried out a short-term NCD experiment to determine which pathways were activated following TrkB.FL overexpression. Hypothalamic samples were collected by 4 weeks post AAV injection. Of note, no changes in body weight were observed in this short-term experiment (S5 Fig). Western blotting was performed to analyze ERK1/2, AKT, and PLCγ phosphorylation states, as well as protein levels of TrkB.FL, TrkB.T1, Ras, and PTEN. The presence of Myc, a tag to the transgene TrkB.FL carried by AAV vector, verified the transgene expression in the hypothalamus. TrkB.FL protein level was significantly elevated in AAV-TrkB.FL animals (P = 0.0002), confirming transduction by the AAV vector. The ratio of TrkB.FL/TrkB.T1 was significantly higher in AAV-TrkB.FL animals (P = 0.0109). TrkB.FL overexpression also significantly increased phospho-PLCγ levels (P = 0.034). There were no significant differences between AAV-TrkB.FL and AAV-YFP animals for TrkB.T1, phospho-AKT, phospho-ERK, Ras, or PTEN (Fig 8).

**Fig 9:**
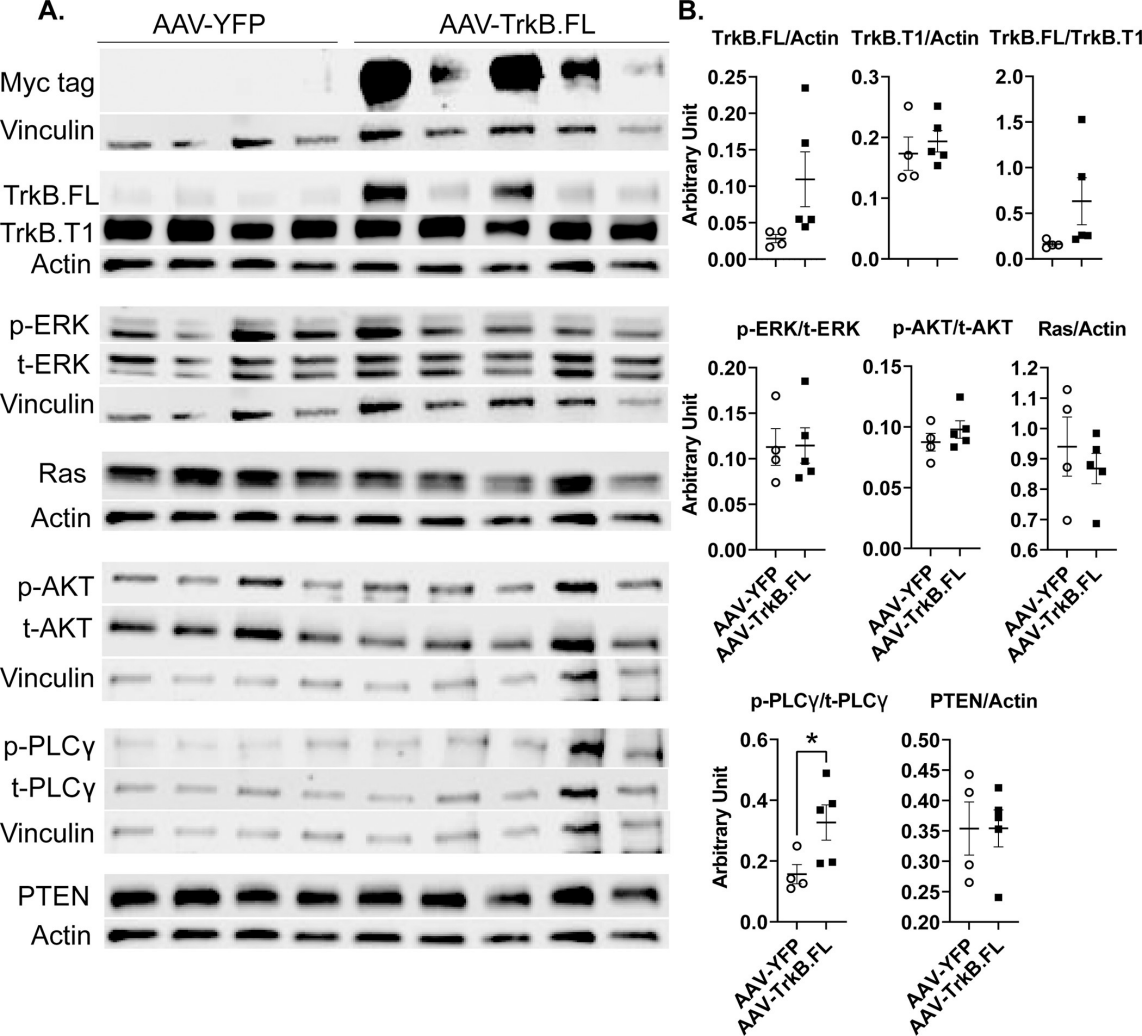
Hypothalamic AAV-TrkB.FL overexpression affects signaling mediators downstream of TrkB in HFD BTBR mice. (A) Western blotting of right lobe of the hypothalamus. (B) Quantification of (A). Data are means ±SEM. AAV-YFP: n = 4, AAV-TrkB.FL: n = 5. * P<0.05.

To determine which pathways were activated following TrkB.FL overexpression in obese BTBR mice, we carried out a short-term HFD experiment. Hypothalamic samples were collected at 4 weeks post AAV injection. As in the short-term NCD experiment, no changes in body weight were observed for this experiment (S6 Fig). We performed Western blotting to analyze the same phosphorylation states and protein levels as in the short-term NCD experiment. The presence of Myc confirmed transgene expression in the hypothalamus. AAV-TrkB.FL animals showed a trend toward increased TrkB.FL (P = 0.10) and a trend toward an increased TrkB.FL/TrkB.T1 ratio (P = 0.11). TrkB.FL overexpression significantly increased phospho-PLCγ levels (P = 0.048). There were no significant differences between AAV-TrkB.FL and AAV-YFP animals for TrkB.T1, phospho-AKT, phospho-ERK, Ras, or PTEN (Fig 9).

### Hypothalamic TrkB.FL gene transfer does not affects signaling mediators downstream of TrkB in C57BL/6 mice

**Fig 10:**
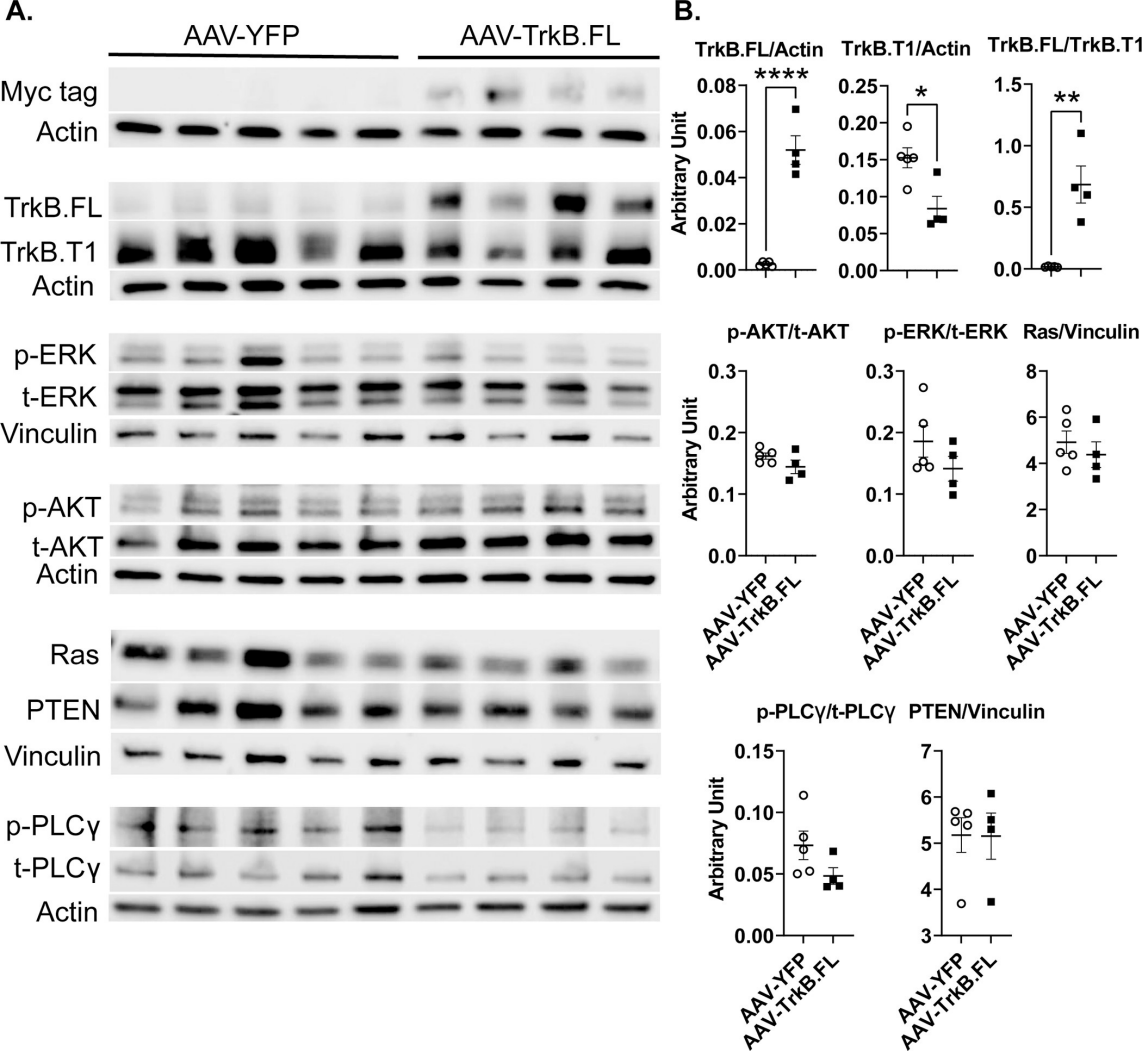
Hypothalamic TrkB.FL gene transfer does not affect signaling mediators downstream of TrkB in C57BL/6 mice. (A) Western blotting of right lobe of the hypothalamus. (B) Quantification of (A). Data are means ±SEM. AAV-YFP: n = 5, AAV-TrkB.FL: n = 4. * P<0.05, ** P<0.01, **** P<0.0001.

To examine hypothalamic signaling changes that occur following TrkB.FL overexpression in C57BL/6 mice of normal weight, we performed a short-term experiment mirroring the BTBR short-term experiments. Hypothalamic samples were collected 4 weeks post injection. There were no changes in body weight over the course of this experiment (S7 Fig). Myc expression verified TrkB.FLtransgene expression in the hypothalamus. Myc was not expressed in one TrkB.FL sample and was thus excluded from further analysis. As seen in the short-term NCD BTBR experiment, AAV-TrkB.FL mice showed significantly higher protein levels of TrkB.FL (P<0.0001) and a significantly higher TrkB.FL/TrkB.T1 ratio as compared to AAV-YFP mice (P = 0.0015). However, overexpressing TrkB.FL in C57BL/6 mice did not alter phospho-AKT, phospho-ERK, Ras, PTEN, or phospho-PLCγ (Fig 10).

## Discussion

In this study, we found that hypothalamic TrkB.FL gene transfer in BTBR mice improved metabolic outcomes, but not behavioral deficits. Both long-term NCD and long-term HFD AAV-TrkB.FL groups displayed lower percent body weight gain in the absence of decrease of food intake. Long-term NCD AAV-TrkB.FL animals showed increased leanness and decreased adiposity as well as improved glycemic control. The effects of TrkB.FL overexpression on metabolic outcomes were not as drastic in HFD-fed animals as in NCD-fed animals, and TrkB.FL NCD-fed mice displayed lower relative weight of iWAT while HFD-fed did not show a difference between AAV-YFP and AAV-TrkB.FL. BTBR animals show metabolic impairments at baseline [14]. It is possible that TrkB.FL overexpression was effective for improving metabolic outcomes in the BTBR mouse under normal feeding conditions, but introducing an additional metabolic disturbance in the form of a HFD was too much for the transgenic TrkB.FL to sufficiently mitigate. An alternative explanation could be that two animals in the HFD TrkB.FL group showed much higher protein levels of TrkB.FL than the other three animals. It is possible that the vector was more successful at transducing TrkB.FL in these animals and that these animals displayed greater changes than the other three animals, suggesting that TrkB.FL is crucially involved in the mechanism leading to improved metabolic outcomes but the administration of the AAV-TrkB.FL vector did not transduce neurons as successfully in three of the animals. Additionally, while the TrkB.FL protein level was elevated in the TrkB.FL HFD group, the elevation did not significantly alter the TrkB.FL/TrkB.T1 ratio as was observed in the TrkB.FL NCD group. TrkB.T1 can block the BDNF-TrkB.FL signaling cascade, so the lack of improved TrkB.FL/TrkB.T1 ratio could be limiting the effects of BDNF [35, 36]. An additional difference in outcomes between NCD and HFD mice was relative liver weight. NCD mice did not show a difference in relative liver weight between AAV-YFP and AAV-TrkB.FL while in HFD mice, TrkB.FL overexpression decreased relative liver weight. HFD has been shown to cause excessive fat deposition in the liver [37]. The HFD BTBR mice displayed both higher absolute liver weight and higher relative liver weight as compared to NCD BTBR mice. It is possible that in the HFD group, TrkB.FL overexpression helped to reduce potential excessive accumulation of lipids in the liver. In general, hypothalamic TrkB.FL overexpression resembles the metabolic outcomes induced by hypothalamic BDNF overexpression in C57BL/6 mice of normal weight as well as genetic obesity and DIO models on C57BL/6 background [7, 38–41]. For both BDNF and TrkB.FL gene transfer studies, AAV1 serotype vectors, largely neuronal-tropic, were used, and therefore transgenes were overexpressed primarily in the neurons. However, BDNF expressed from AAV1 vector can be secreted from the transduced neurons to act on other cell types within the hypothalamus or transport to extrahypothalamic area. In contrast, overexpressed TrkB.FL receptor remains on the transduced neurons, Hence, TrkB.FL overexpression data from the current study suggest hypothalamic neuronal BDNF-TrkB signaling is likely the main action mode for the metabolic benefits associated with either increasing the level of BDNF ligand or the level of TrkB receptor. BDNF can be expressed in different cell types such as microglia and astrocytes [42–44]. It is possible that BDNF is eliciting its effects on metabolism through neuronal BDNF-neuronal TrkB signaling, microglial BDNF-neuronal TrkB signaling, astrocytic BDNF-neuronal TrkB signaling, or a combination of several signaling mechanisms.

One notable finding of the study is the downregulation of proinflammatory *Il1b* expression and alteration of the expression of some microglial-related genes in the hypothalamus of AAV-TrkB.FL injected mice. These molecular changes were observed in the long-term NCD study a few months after systemic metabolic effects occurred. We did not see these inflammatory changes in the long-term HFD study. Mice fed a high-fat diet display chronic hypothalamic inflammation [45, 46]. BDNF-TrkB is involved in regulating neuroinflammation [47, 48]. It is possible that in the long-term NCD study, upregulation of TrkB.FL was involved in mitigating neuroinflammation, but in the long-term HFD study perhaps the level of transgenic TrkB.FL was not sufficient to overcome the baseline neuroinflammation involved in HFD feeding. Additionally, we observed upregulation of *Pomc* and *Npy* in TrkB.FL NCD mice, but not in TrkB.FL HFD mice. Pomc and Npy expressing neurons act antagonistically to control energy homeostasis, and a disruption in this system is associated with obesity. Particularly, a HFD downregulates *Pomc* and *Npy* [49, 50]. Again, perhaps the level of transgenic TrkB.FL was not sufficient to overcome the baseline lower levels of *Pomc* and *Npy* involved in HFD feeding. New studies are required to investigate whether the potential modulation of neuroinflammation and/or microglial functions is a direct effect of hypothalamic neuronal BDNF-TrkB activation, or alternatively, a feedback of the systemic metabolic outcomes induced by hypothalamic TrkB.FL.

Research points toward impaired neural circuit development as a potential cause of ASD. Due to the importance of neurotrophins in synaptic plasticity and neural growth and development, alterations in neurotrophin levels and their associated signaling pathways are one area of research into the pathophysiology of ASD in human subjects and in animal models of ASD [51]. Several studies have found a decrease in mRNA and protein levels of BDNF and its receptor TrkB in the hippocampus and cortex of BTBR mice [52–55]. One study found that the use of a TrkB receptor agonist, 7–8, DHF, in the BTBR mouse model reversed several aspects of social deficits [56]. Several studies have found an up-regulation of the Ras/Raf/Erk1/2 pathway in the hippocampus and prefrontal cortex of BTBR mice, which may contribute to the pathogenesis of ASD [57–59]. However, no research has examined these pathways in the hypothalamus of the BTBR mouse.

BDNF-TrkB activation initiates three main signaling pathways—MAPK, PI3K, and PLCγ. Activation of TrkB at its Tyr490 and Tyr515 sites results in recruitment of GTPase Ras, which activates the MAPK/ERK pathway. MAPK/ERK pathway activation ultimately drives BDNF expression to regulate neuronal survival, differentiation, and synaptic plasticity [34]. Recruitment of Ras at the TrkB Tyr515 site also activates the PI3K/AKT pathway. Activation of this pathway regulates proteins that are essential for neuronal growth, differentiation, and survival [34]. Finally, TrkB phosphorylation at the Tyr816 residue activates the PLCγ pathway, which is important for survival, neurite outgrowth, and synaptic plasticity [34]. In addition, our previous EE-BTBR study indicated that *Bdnf* and its receptor *Ntrk2* were both up-regulated, suggesting BDNF-TrkB signaling plays a role in mediating the EE-BTBR phenotype. Therefore, hypothalamic TrkB.FL overexpression in BTBR mice would also promote its downstream pathway signaling, thereby leading to the improved beneficial outcomes we saw in the EE-BTBR study.

In the NCD group, strong Myc bands in the AAV-TrkB.FL group confirmed on-target transduction of the AAV vector (Fig 8A). There was a significant increase in TrkB.FL protein level in the AAV-TrkB.FL mice compared to the AAV-YFP mice. In BTBR mice, studies have found that TrkB levels are significantly lower in the hippocampus than in C57BL/6 mice [54], but no data is available regarding relative levels of TrkB in the hypothalamus.

In this study, we assessed the ratio of TrkB.FL to TrkB.T1. TrkB.T1 is a truncated isoform of TrkB that does not contain an intracellular kinase domain, and therefore it does not activate the classical signaling pathways associated with TrkB.FL. TrkB.T1 can bind BDNF to prevent activation of the MAPK/ERK, PI3K/AKT, and PLCγ pathways by forming a heterodimer with TrkB.FL, acting as a dominant negative inhibitor of TrkB.FL. Increased expression of TrkB.T1 can thus prevent BDNF mediated cell survival and proliferation [60]. Our previous studies have indicated that hypothalamic overexpression of TrkB.T1 can block BDNF-TrkB signaling [7, 10]. Additionally, research has also found an association between neuropsychiatric and neurodegenerative disorders and altered levels of TrkB.FL and TrkB.T1 isoforms [24, 61, 62]. One study also showed that reducing TrkB.T1 levels *in vivo* in a *Bdnf* heterozygous knockout mouse partially rescued the obesity phenotype [63]. Here, TrkB.FL overexpression in BTBR mice increased the TrkB.FL/TrkB.T1 ratio and may contribute to the improved metabolic outcomes we saw.

pPLCγ was significantly increased in the AAV-TrkB.FL group, leading us to believe that the pPLCγ pathway is important for the improved metabolic outcomes we saw in the NCD group. As previously mentioned, activation of the PLCγ pathway is important for synaptic plasticity. Over the course of the NCD experiment, AAV-TrkB.FL animals ate significantly more relative food (Fig 1D), but gained significantly less weight (Fig 1C). It is possible that overexpressing hypothalamic TrkB.FL activates the PLCγ pathway, leading to synaptic plasticity that increases energy expenditure, improving downstream metabolic outcomes.

To investigate a mechanism downstream of hypothalamic TrkB.FL signaling that may explain the improved metabolic outcomes, we profiled a number of metabolism related genes in iWAT, gWAT, and BAT. WAT and BAT make up an organism’s adipose organ. WAT stores excess energy and acts as an endocrine organ, while BAT is metabolically active and dissipates energy as heat for thermoregulation. BAT dissipates energy as heat through thermogenesis, whereby uncoupling protein-1 (UCP1) mediates the process of uncoupling fatty acid oxidation from ATP production [64]. BAT is also involved in body weight regulation and contributes to energy expenditure. WAT can acquire brown fat characteristics in response to chronic cold exposure or prolonged B-adrenergic stimulation through a process termed “browning” or “beiging”, which is associated with increased energy expenditure and resistance against obesity [64–67]. Our previous studies have found a mechanism that links activation of hypothalamic BDNF to increased energy expenditure, termed the HSA axis. We discovered that upregulation of hypothalamic BDNF leads to increased sympathetic tone to WAT adipocytes via β-adrenergic signaling within the sympathetic nervous system (SNS). This increased sympathetic tone induces WAT “beiging”, a decrease of adipocyte leptin production and secretion, and an upregulation of adiponectin level, leading to increased energy expenditure and improved metabolic outcomes [7, 10, 38]. Moreover, environmental or genetic upregulation of hypothalamic BDNF prevents aging-related decline of BAT [38, 68].

Based on these previous studies we hypothesized that overexpression of TrkB.FL, similar to upregulation of BDNF, would activate the HSA axis leading to adipose remodeling and metabolic improvement. Adiponectin and leptin levels are both associated with adiposity, with adiponectin expression being reduced as adiposity is increased, and leptin being increased as adiposity is increased [69]. We found that in TrkB.FL overexpressing mice, gWAT *Adipoq* was upregulated and *Lep* was downregulated and iWAT *Adipoq* was upregulated. This is consistent with the reduction in body weight we observed, as well as our previous studies showing that activation of the HSA axis increases adiponectin and decreases leptin in WAT adipocytes. β-adrenergic signaling is important for the activation of BAT in response to cold and for the regulation of adiposity and it also plays an important role in inducing beiging of WAT [70, 71]. We found that in all three fat depots (iWAT, gWAT, BAT), TrkB.FL overexpression upregulated gene expression of *Adrb3*, suggesting increased sympathetic tone to the adipose tissues and possible induction of beiging in the WAT. *Hsl* was upregulated in gWAT of TrkB.FL overexpressing mice, suggesting increased lipolysis in WAT [72]. Our previous studies identified *Vegfa* as a key component of the HSA axis underlying the beiging effect of WAT, likely directly caused by the SNS stimulation to the WAT [10]. We found that TrkB.FL overexpression upregulated *Vegfa* expression in gWAT, suggesting beiging may be occurring in WAT. *Ppargc1a* encodes for PGC-1α, which has been shown to be an important modulator of UCP1 expression and thermogenesis in BAT and also plays an important role in the brown adipocyte differentiation process [73]. We found that *Ppargc1a* was upregulated in the iWAT of TrkB.FL overexpressing animals, which suggests beiging of WAT. *Prdm16* controls a cell fate switch determining formation and function of brown adipocytes [74] and was upregulated in BAT of TrkB.FL mice. This was accompanied by upregulation of a BAT molecular signature, including *Cidea*, *Elovl3*, and *Ucp1*, which are all BAT selective markers and are positively regulated by *Prdm16* [74]. Overall, the gene expression profile of iWAT, gWAT, and BAT demonstrates that hypothalamic TrkB.FL gene transfer induced adipose gene expression signature overlapped with hypothalamic BDNF gene transfer, which is known to increase energy expenditure and improves metabolic outcomes mediated by the HSA axis.

TrkB.FL overexpression resulted in signaling changes in the HFD group similar to those in the NCD group. Just as in the NCD group, TrkB.FL overexpression in the HFD group significantly increased phospho-PLCy, while there were no significant differences for phospho-AKT, phospho-ERK, PTEN, or Ras. Thus, it seems that the PLCγ pathway is important for the improved metabolic outcomes in both NCD BTBR mice and HFD BTBR mice. In the AAV-TrkB.FL animals, increased TrkB.FL levels and the increased TrkB.FL/TrkB.T1 ratio trended toward significance, but did not reach the highly increased levels found in the NCD study. In C57BL/6 mice, a HFD has been found to decrease hippocampal TrkB activation and expression and reduce activation of hippocampal BDNF-TrkB signaling pathways [75, 76]. It’s possible that the HFD blunted the effects of the AAV vector mediated TrkB.FL overexpression. This is the first study examining the effects of a HFD on the hypothalamus of BTBR mice; more investigation is needed to understand the complex molecular and signaling interactions between diet and the BTBR model.

To investigate whether the effects of TrkB.FL overexpression can be generalized to non-BTBR mice, we injected C57BL/6 mice with either TrkB.FL or YFP and examined protein expression of signaling mediators downstream of TrkB.FL. Similar to BTBR NCD mice, we found that TrkB.FL overexpression altered the ratio of TrkB.FL/TrkB.T1. In contrast to BTBR mice we found that TrkB.FL overexpression did not upregulate pPLCγ, suggesting strain-dependent response to TrkB.FL overexpression in mice of normal weight. We did not perform behavioral testing for C57Bl/6J mice in this study. The purpose of including C57Bl/6J mice was to compare the changes in TrkB.FL signaling pathways following TrkB.FL overexpression between C57Bl/6J mice and BTBR mice. Because C57Bl/6J mice do not have baseline deficits in behavior and sociability like BTBR mice we did not expect to see any significant changes in these groups following TrkB.FL overexpression. We have not overexpressed neuronal TrkB.FL in C57Bl/6 mice before, but we have overexpressed neuronal BDNF in the same area of the brain. We found that in C57Bl/6 mice fed a NCD, overexpressing BDNF led to weight loss; reduced adiposity; a decrease in serum leptin, insulin, cholesterol, triglycerides, and IGF-1 and an increase in adiponectin; and altered expression of metabolism related genes in the hypothalamus and adipose tissue [8, 39]. In C57Bl/6 mice fed a HFD to induce DIO, we found that overexpressing BDNF led to prevention of weight gain and weight loss; prevention of abdominal obesity; a decrease in serum leptin, insulin, cholesterol, triglycerides, IGF-1, and glucose and an increase in adiponectin; improved insulin sensitivity and glucose tolerance; prevention of liver steatosis; and altered expression of metabolism related genes in the hypothalamus and adipose tissue [7, 39].

In our previous EE-BTBR study, we found EE improved metabolic outcomes and several behavioral measures. Because of our additional prior studies showing that hypothalamic BDNF mediates the improved metabolism and cancer outcomes in previous obesity and cancer EE studies, we hypothesized that TrkB signaling was also responsible for the outcomes we saw in the BTBR EE study. In the current study, we found that hypothalamic TrkB.FL overexpression specifically in the hypothalamus of BTBR mice improved the metabolic outcomes, but not behavioral deficits. This suggests that there is an additional mechanism responsible for the EE-induced behavioral improvements in BTBR mice. Much of the research surrounding BTBR behavior has focused on characterizing the behavioral phenotypes [77, 78] or administering pharmacological or environmental interventions and assaying behavioral outcomes [79–82]. Less research has focused on the biological mechanisms underlying the behavioral outcomes. It seems likely that the behavioral phenotypes of BTBR mice are at least partially associated with deficits in the hippocampus or amygdala, as these are important brain regions for learning and memory [83] and emotion and motivation [84], respectively. Our previous study found that *Ntrk2* was significantly upregulated by 3 to 6 folds in the hypothalamus, hippocampus, and amygdala of male mice upon EE exposure [13]. It is possible that the improved metabolic outcomes in our BTBR EE study were due to enhanced hypothalamic BDNF-TrkB signaling, as seen in our current study, and the improved behavioral outcomes might involve BDNF-TrkB signaling in other brain regions, such as the hippocampus and amygdala. Studies have found that feeding BTBR mice a HFD exacerbates autistic-like behaviors. One study found that feeding BTBR mice a HFD induced severe metabolic impairments including weight gain, increase in fat mass and decrease in lean mass, and decrease in energy expenditure. Mice fed with a HFD displayed enhanced cognitive rigidity and impaired social memory. Autism-like severity was associated with body weight and dopaminergic signaling in the hypothalamus [21]. Another study found that feeding BTBR mice a HFD led to an increase in body weight and induced fasting hyperglycemia and glucose intolerance. HFD feeding increased hyperactivity, rescued sociability but not social novelty in the three-chamber sociability test, and aggravated self-grooming repetitive behavior [85]. These studies suggest that in the BTBR model of ASD, metabolism and behavior may be linked. More research is needed to connect behavioral outcomes to the neurobiological mechanisms of the BTBR mouse. Age is another factor to investigate. Studies have found levels of BDNF and TrkB differ throughout the lifespan of BTBR mice, with fetal BTBR mice expressing significantly higher brain BDNF protein levels than fetal FVB/NJ mice [86] while aged BTBR mice expressing significantly lower hippocampal and cortical BDNF protein levels compared to age-matched C57BL/6 controls [53]. Future studies could investigate the differences in effectiveness of overexpressing hypothalamic TrkB.FL at different age points throughout the lifespan.

Another path to examine is sex differences. Our previous EE-BTBR study found significant sex-dependent outcomes. EE induced more robust improvements in metabolic outcomes in male mice, with only modest improvements in females. It is worthy of noting that upregulation of *Ntrk2* induced by EE was only observed in male mice [13], which promoted us to investigate TrkB.FL overexpression in male BTBR mice in the current study. However, BDNF-TrkB signaling should not be ignored in the females. In fact, there are also significant sex differences in prevalence and presentation of ASD in humans [87], so investigating the mechanisms underlying sex differences in BTBR mice and in people with ASD is an important area to investigate. Genetic manipulation of TrkB in specific brain regions could help to interrogate signaling pathways underlying the sex differences we found in our EE-BTBR study. Finally, to definitively confirm that hypothalamic BDNF-TrkB signaling is indeed critical for EE-induced metabolic benefits in BTBR mice, we can house BTBR mice in an EE while knocking out hypothalamic TrkB.FL to see if it blocks the metabolic improvements with similar strategies as in our previous studies [7, 8].

One drawback to this study are the age differences and the differences in time points of metabolic and behavioral measurements between the long-term NCD group and the long-term HFD group. As such, we were unable to directly compare these two groups. Age-matched experiments would have allowed us to examine exact differences in response to hypothalamic TrkB.FL overexpression between BTBR mice of a normal weight and obese BTBR mice and controlled for possible age effects. Unfortunately, COVID-19 restrictions placed limitations on the timings of the experiments and breeding availability. Due to these age differences we must note that direct comparisons or concrete conclusions cannot be made regarding the differences between the long-term NCD and long-term HFD groups.

To date, this is the first study investigating hypothalamic BDNF-TrkB signaling pathways and their impact on metabolism and behavior in the BTBR mouse model of ASD or in a diet-induced obesity BTBR model. Dysregulated BDNF-TrkB signaling has been implicated in both ASD and in obesity, with dysfunction of the hypothalamus being critical in obesity, but the changes in signaling mediators downstream of hypothalamic TrkB in BTBR mice have not been studied sufficiently. Here we provide evidence that hypothalamic TrkB.FL overexpression changes downstream signaling mediators, leading to improved metabolic outcomes in the BTBR mouse model of ASD.

## Supporting information

S1 Data (XLSX)

S2 Data (XLSX)

S3 Data (XLSX)

S1 Raw images (PDF)

S1 Text Behavioral methods. (DOCX)

S1 Table Primer sequences used for qPCR. (DOCX)

S2 Table Primary antibodies used for western blotting. (DOCX)

S1 Fig YFP fluorescence in the hypothalamus of long-term NCD mouse injected with AAV-YFP. Representative image was taken at 10x magnification. ARC, arcuate nucleus; VMH, ventromedial hypothalamus.
(TIF)

S2 Fig Open Field Test in NCD BTBR mice. (A) Open field test total distance. (B) Open field test center/total distance ratio. (C) Open field test periphery/total distance ratio. Data are means ±SEM. AAV-YFP: n = 8, AAV-TrkB.FL: n = 8.
(TIF)

S3 Fig Open Field Test, novelty suppressed feeding, and cold-induced defecation in HFD BTBR mice. (A) Open field test total distance. (B) Open field test center/total distance ratio. (C) Open field test periphery/total distance ratio. (D) Novelty suppressed feeding food consumed. (E) Novelty suppressed feeding latency to feed. (F) Cold induced defecation fecal boli. Data are means ±SEM. AAV-YFP: n = 5, AAV-TrkB.FL: n = 6.
(TIF)

S4 Fig Three chamber sociability test in HFD BTBR mice. (A) Three chamber sociability social affiliation. (B) Three chamber test sociability social affiliation index. (C) Three chamber sociability test social novelty. (D) Three chamber sociability test social novelty index. Data are means ±SEM. AAV-YFP: n = 5, AAV-TrkB.FL: n = 6.
(TIF)

S5 Fig Body weight in short-term NCD BTBR mice. (A) Body weight. Data are means ±SEM. AAV-YFP: n = 5, AAV-TrkB.FL: n = 4.
(TIF)

S6 Fig Body weight in short-term HFD BTBR mice. (A) Body weight. Data are means ±SEM. AAV-YFP: n = 4, AAV-TrkB.FL: n = 5.
(TIF)

S7 Fig Body weight in short-term NCD C57BL/6 mice. (A) Body weight. Data are means ±SEM. AAV-YFP: n = 5, AAV-TrkB.FL: n = 5.
(TIF)

## References

[1] Association AP. Neurodevelopmental Disorders, Diagnostic and Statistical Manual of Mental Disorders. 2013;5th ed. American Psychiatric Association, Washington, DC.

[2] Maenner MS, KA; Baio J; et al. Prevalence of Autism Spectrum Disorder Among Children Aged 8 Years—Autism and Developmental Disabilities Monitoring Network, 11 Sites, United States, 2016. MMWR Surveill Summ 2020. 2020;69(SS-4):1–12. 10.15585/mmwr.ss6904a1 PMC7119644 32214087

[3] Hodges H, Fealko C, Soares N. Autism spectrum disorder: definition, epidemiology, causes, and clinical evaluation. Transl Pediatr. 2020;9(Suppl 1):S55–S65. doi: 10.21037/tp.2019.09.09 32206584 PMC7082249

[4] Masi A, DeMayo MM, Glozier N, Guastella AJ. An Overview of Autism Spectrum Disorder, Heterogeneity and Treatment Options. Neurosci Bull. 2017;33(2):183–93. doi: 10.1007/s12264-017-0100-y 28213805 PMC5360849

[5] Sharma SR, Gonda X, Tarazi FI. Autism Spectrum Disorder: Classification, diagnosis and therapy. Pharmacol Ther. 2018;190:91–104. doi: 10.1016/j.pharmthera.2018.05.007 29763648

[6] Kodak T, Bergmann S. Autism Spectrum Disorder: Characteristics, Associated Behaviors, and Early Intervention. Pediatr Clin North Am. 2020;67(3):525–35. doi: 10.1016/j.pcl.2020.02.007 32443991

[7] Cao L, Choi EY, Liu X, Martin A, Wang C, Xu X, et al. White to brown fat phenotypic switch induced by genetic and environmental activation of a hypothalamic-adipocyte axis. Cell Metab. 2011;14(3):324–38. doi: 10.1016/j.cmet.2011.06.020 21907139 PMC3172615

[8] Cao L, Liu X, Lin EJ, Wang C, Choi EY, Riban V, et al. Environmental and genetic activation of a brain-adipocyte BDNF/leptin axis causes cancer remission and inhibition. Cell. 2010;142(1):52–64. doi: 10.1016/j.cell.2010.05.029 20603014 PMC3784009

[9] Foglesong GD, Queen NJ, Huang W, Widstrom KJ, Cao L. Enriched environment inhibits breast cancer progression in obese models with intact leptin signaling. Endocr Relat Cancer. 2019;26(5):483–95. doi: 10.1530/ERC-19-0075 30856610 PMC6717689

[10] During MJ LX, Huang W, Magee D, Slater A, McMurphy T, Wang C, Cao L. Adipose VEGF Links the White-to-Brown Fat Switch With Environmental, Genetic, and Pharmacological Stimuli in Male Mice. Endocrinology. 2015;156(6):2059–73. doi: 10.1210/en.2014-1905 25763639 PMC4430610

[11] Huang wQ N.J.; McMurphy T.B.; Ali S.; Wilkins R.K.; Appana B.; Cao L. Adipose PTEN acts as a downstream mediator of a brain-fat axis in environmental enrichment. Comprehensive Psychoneuroendocrinology. 2020;4. 10.1016/j.cpnec.2020.100013 PMC8963210 35355831

[12] Bergin SM, Xiao R, Huang W, Judd CRT, Liu X, Mansour AG, et al. Environmental activation of a hypothalamic BDNF-adipocyte IL-15 axis regulates adipose-natural killer cells. Brain Behav Immun. 2021;95:477–88. doi: 10.1016/j.bbi.2021.05.005 33989745 PMC8493653

[13] Queen NJ, Boardman AA, Patel RS, Siu JJ, Mo X, Cao L. Environmental enrichment improves metabolic and behavioral health in the BTBR mouse model of autism. Psychoneuroendocrinology. 2020;111:104476. doi: 10.1016/j.psyneuen.2019.104476 31648110 PMC6914218

[14] Meyza KZ, Blanchard DC. The BTBR mouse model of idiopathic autism—Current view on mechanisms. Neurosci Biobehav Rev. 2017;76(Pt A):99–110. doi: 10.1016/j.neubiorev.2016.12.037 28167097 PMC5403558

[15] Karunakaran S, Manji A, Yan CS, Wu ZJ, Clee SM. Moo1 obesity quantitative trait locus in BTBR T+ Itpr3tf/J mice increases food intake. Physiol Genomics. 2013;45(5):191–9. doi: 10.1152/physiolgenomics.00159.2012 23341217

[16] Rabaglia ME, Gray-Keller MP, Frey BL, Shortreed MR, Smith LM, Attie AD. Alpha-Ketoisocaproate-induced hypersecretion of insulin by islets from diabetes-susceptible mice. Am J Physiol Endocrinol Metab. 2005;289(2):E218–24. doi: 10.1152/ajpendo.00573.2004 15741243

[17] Flowers JB, Oler AT, Nadler ST, Choi Y, Schueler KL, Yandell BS, et al. Abdominal obesity in BTBR male mice is associated with peripheral but not hepatic insulin resistance. Am J Physiol Endocrinol Metab. 2007;292(3):E936–45. doi: 10.1152/ajpendo.00370.2006 17132824

[18] Meyza KZ, Defensor EB, Jensen AL, Corley MJ, Pearson BL, Pobbe RL, et al. The BTBR T+ tf/J mouse model for autism spectrum disorders-in search of biomarkers. Behav Brain Res. 2013;251:25–34. doi: 10.1016/j.bbr.2012.07.021 22958973 PMC3529977

[19] McFarlane HG, Kusek GK, Yang M, Phoenix JL, Bolivar VJ, Crawley JN. Autism-like behavioral phenotypes in BTBR T+tf/J mice. Genes Brain Behav. 2008;7(2):152–63. doi: 10.1111/j.1601-183X.2007.00330.x 17559418

[20] Mizuno S, Hirota JN, Ishii C, Iwasaki H, Sano Y, Furuichi T. Comprehensive Profiling of Gene Expression in the Cerebral Cortex and Striatum of BTBRTF/ArtRbrc Mice Compared to C57BL/6J Mice. Front Cell Neurosci. 2020;14:595607. doi: 10.3389/fncel.2020.595607 33362469 PMC7758463

[21] Zilkha N, Kuperman Y, Kimchi T. High-fat diet exacerbates cognitive rigidity and social deficiency in the BTBR mouse model of autism. Neuroscience. 2017;345:142–54. doi: 10.1016/j.neuroscience.2016.01.070 26855190

[22] Hill AP, Zuckerman KE, Fombonne E. Obesity and Autism. Pediatrics. 2015;136(6):1051–61. doi: 10.1542/peds.2015-1437 26527551 PMC4657601

[23] Zheng Z, Zhang L, Li S, Zhao F, Wang Y, Huang L, et al. Association among obesity, overweight and autism spectrum disorder: a systematic review and meta-analysis. Sci Rep. 2017;7(1):11697. doi: 10.1038/s41598-017-12003-4 28916794 PMC5601947

[24] Fenner ME, Achim CL, Fenner BM. Expression of full-length and truncated trkB in human striatum and substantia nigra neurons: implications for Parkinson’s disease. J Mol Histol. 2014;45(3):349–61. doi: 10.1007/s10735-013-9562-z 24374887

[25] Cao L, Jiao X, Zuzga DS, Liu Y, Fong DM, Young D, et al. VEGF links hippocampal activity with neurogenesis, learning and memory. Nat Genet. 2004;36(8):827–35. doi: 10.1038/ng1395 15258583

[26] Livak KJ, Schmittgen TD. Analysis of relative gene expression data using real-time quantitative PCR and the 2(-Delta Delta C(T)) Method. Methods. 2001;25(4):402–8. doi: 10.1006/meth.2001.1262 11846609

[27] Okano S, Nagaya H, Inatomi N. Novelty stress increases fecal pellet output in mongolian gerbils: effects of several drugs. J Pharmacol Sci. 2005;98(4):411–8. doi: 10.1254/jphs.fp0050353 16079466

[28] Kizaki T, Oh-ishi S, Ohno H. Acute cold stress induces suppressor macrophages in mice. Journal of Applied Physiology. 1996;81(1):393–9. doi: 10.1152/jappl.1996.81.1.393 8828690

[29] Kaidanovich-Beilin O, Lipina T, Vukobradovic I, Roder J, Woodgett JR. Assessment of social interaction behaviors. J Vis Exp. 2011(48). doi: 10.3791/2473 21403628 PMC3197404

[30] Crawley JN. Designing mouse behavioral tasks relevant to autistic-like behaviors. Ment Retard Dev Disabil Res Rev. 2004;10(4):248–58. doi: 10.1002/mrdd.20039 15666335

[31] Crawley JN. Mouse behavioral assays relevant to the symptoms of autism. Brain Pathol. 2007;17(4):448–59. doi: 10.1111/j.1750-3639.2007.00096.x 17919130 PMC8095652

[32] Heo Y, Zhang Y, Gao D, Miller VM, Lawrence DA. Aberrant immune responses in a mouse with behavioral disorders. PLoS One. 2011;6(7):e20912. doi: 10.1371/journal.pone.0020912 21799730 PMC3140472

[33] Huang EJ, Reichardt LF. Trk receptors: roles in neuronal signal transduction. Annu Rev Biochem. 2003;72:609–42. doi: 10.1146/annurev.biochem.72.121801.161629 12676795

[34] Pradhan J, Noakes PG, Bellingham MC. The Role of Altered BDNF/TrkB Signaling in Amyotrophic Lateral Sclerosis. Front Cell Neurosci. 2019;13:368. doi: 10.3389/fncel.2019.00368 31456666 PMC6700252

[35] Fenner BM. Truncated TrkB: beyond a dominant negative receptor. Cytokine Growth Factor Rev. 2012;23(1–2):15–24. doi: 10.1016/j.cytogfr.2012.01.002 22341689

[36] Tessarollo L, Yanpallewar S. TrkB Truncated Isoform Receptors as Transducers and Determinants of BDNF Functions. Front Neurosci. 2022;16:847572. doi: 10.3389/fnins.2022.847572 35321093 PMC8934854

[37] Lian CY, Zhai ZZ, Li ZF, Wang L. High fat diet-triggered non-alcoholic fatty liver disease: A review of proposed mechanisms. Chem Biol Interact. 2020;330:109199. doi: 10.1016/j.cbi.2020.109199 32805210

[38] Liu X, McMurphy T, Xiao R, Slater A, Huang W, Cao L. Hypothalamic gene transfer of BDNF inhibits breast cancer progression and metastasis in middle age obese mice. Mol Ther. 2014;22(7):1275–84. doi: 10.1038/mt.2014.45 24637454 PMC4089014

[39] Cao L, Lin EJ, Cahill MC, Wang C, Liu X, During MJ. Molecular therapy of obesity and diabetes by a physiological autoregulatory approach. Nat Med. 2009;15(4):447–54. doi: 10.1038/nm.1933 19270710 PMC3900280

[40] Siu JJ, Queen NJ, Liu X, Huang W, McMurphy T, Cao L. Molecular Therapy of Melanocortin-4-Receptor Obesity by an Autoregulatory BDNF Vector. Mol Ther Methods Clin Dev. 2017;7:83–95. doi: 10.1016/j.omtm.2017.09.005 29296625 PMC5744069

[41] McMurphy T, Huang W, Liu X, Siu JJ, Queen NJ, Xiao R, et al. Hypothalamic gene transfer of BDNF promotes healthy aging in mice. Aging Cell. 2019;18(2):e12846. doi: 10.1111/acel.12846 30585393 PMC6413658

[42] Ameroso D, Meng A, Chen S, Felsted J, Dulla CG, Rios M. Astrocytic BDNF signaling within the ventromedial hypothalamus regulates energy homeostasis. Nat Metab. 2022;4(5):627–43. doi: 10.1038/s42255-022-00566-0 35501599 PMC9177635

[43] Parkhurst CN, Yang G, Ninan I, Savas JN, Yates JR, 3rd, Lafaille JJ, et al. Microglia promote learning-dependent synapse formation through brain-derived neurotrophic factor. Cell. 2013;155(7):1596–609. doi: 10.1016/j.cell.2013.11.030 24360280 PMC4033691

[44] Urabe H, Kojima H, Chan L, Terashima T, Ogawa N, Katagi M, et al. Haematopoietic cells produce BDNF and regulate appetite upon migration to the hypothalamus. Nat Commun. 2013;4:1526. doi: 10.1038/ncomms2536 23443554 PMC3640364

[45] Santos LS, Cordeiro GS, Matos RJB, Perez GS, Silva RT, Boaventura GT, et al. High-fat diet promotes hypothalamic inflammation in animal models: a systematic review. Nutr Rev. 2022;80(3):392–9. doi: 10.1093/nutrit/nuab033 34010412

[46] Kim JD, Yoon NA, Jin S, Diano S. Microglial UCP2 Mediates Inflammation and Obesity Induced by High-Fat Feeding. Cell Metab. 2019;30(5):952–62 e5. doi: 10.1016/j.cmet.2019.08.010 31495690 PMC7251564

[47] Han R, Liu Z, Sun N, Liu S, Li L, Shen Y, et al. BDNF Alleviates Neuroinflammation in the Hippocampus of Type 1 Diabetic Mice via Blocking the Aberrant HMGB1/RAGE/NF-kappaB Pathway. Aging Dis. 2019;10(3):611–25. 31165005 10.14336/AD.2018.0707 PMC6538223

[48] Wu SY, Pan BS, Tsai SF, Chiang YT, Huang BM, Mo FE, et al. BDNF reverses aging-related microglial activation. J Neuroinflammation. 2020;17(1):210. doi: 10.1186/s12974-020-01887-1 32664974 PMC7362451

[49] Lin S, Storlien L.H., Huang X. Leptin receptor, NPY, POMC mRNA expression in the diet-induced obese mouse brain. Brain Research. 2000;875(1–2):89–95. doi: 10.1016/s0006-8993(00)02580-4 10967302

[50] Vohra MS, Benchoula K, Serpell CJ, Hwa WE. AgRP/NPY and POMC neurons in the arcuate nucleus and their potential role in treatment of obesity. Eur J Pharmacol. 2022;915:174611. doi: 10.1016/j.ejphar.2021.174611 34798121

[51] Reim D. SMJ. Neurotrophic Factors in Mouse Models of Autism Spectrum Disorder: Focus on BDNF and IGF-1. Schmeisser M, Boeckers T (eds) Translational Anatomy and Cell Biology of Autism Spectrum Disorder Advances in Anatomy, Embryology and Cell Biology. 2017;224. doi: 10.1007/978-3-319-52498-6_7 28551754

[52] Daimon CM, Jasien JM, Wood WH, 3rd, Zhang Y, Becker KG, Silverman JL, et al. Hippocampal Transcriptomic and Proteomic Alterations in the BTBR Mouse Model of Autism Spectrum Disorder. Front Physiol. 2015;6:324. doi: 10.3389/fphys.2015.00324 26635614 PMC4656818

[53] Jasien JM, Daimon CM, Wang R, Shapiro BK, Martin B, Maudsley S. The effects of aging on the BTBR mouse model of autism spectrum disorder. Front Aging Neurosci. 2014;6:225. doi: 10.3389/fnagi.2014.00225 25225482 PMC4150363

[54] Scattoni ML, Martire A, Cartocci G, Ferrante A, Ricceri L. Reduced social interaction, behavioural flexibility and BDNF signalling in the BTBR T+ tf/J strain, a mouse model of autism. Behav Brain Res. 2013;251:35–40. doi: 10.1016/j.bbr.2012.12.028 23270976

[55] Stephenson DT, O’Neill SM, Narayan S, Tiwari A, Arnold E, Samaroo HD, et al. Histopathologic characterization of the BTBR mouse model of autistic-like behavior reveals selective changes in neurodevelopmental proteins and adult hippocampal neurogenesis. Mol Autism. 2011;2(1):7. doi: 10.1186/2040-2392-2-7 21575186 PMC3135520

[56] Rhine MA, Parrott JM, Schultz MN, Kazdoba TM, Crawley JN. Hypothesis-driven investigations of diverse pharmacological targets in two mouse models of autism. Autism Res. 2019;12(3):401–21. doi: 10.1002/aur.2066 30653853 PMC6402976

[57] Yang K, Cao F, Sheikh AM, Malik M, Wen G, Wei H, et al. Up-regulation of Ras/Raf/ERK1/2 signaling impairs cultured neuronal cell migration, neurogenesis, synapse formation, and dendritic spine development. Brain Struct Funct. 2013;218(3):669–82. doi: 10.1007/s00429-012-0420-7 22555958

[58] Cheng N, Alshammari F, Hughes E, Khanbabaei M, Rho JM. Dendritic overgrowth and elevated ERK signaling during neonatal development in a mouse model of autism. PLoS One. 2017;12(6):e0179409. doi: 10.1371/journal.pone.0179409 28609458 PMC5469475

[59] Faridar A J-DD, Rider E, Li J, Gobius I, Morcom L, Richards LJ, et al. Mapk/Erk activation in an animal model of social deficits shows a possible link to autism. Mol Autism. 2014;5(57). 10.1186/2040-2392-5-57 PMC4396809 25874073

[60] Cao T, Matyas JJ, Renn CL, Faden AI, Dorsey SG, Wu J. Function and Mechanisms of Truncated BDNF Receptor TrkB.T1 in Neuropathic Pain. Cells. 2020;9(5). doi: 10.3390/cells9051194 32403409 PMC7290366

[61] Dwivedi Y RH, Conley RR, Roberts RC, Tamminga CA, Pandey GN. Altered Gene Expression of Brain-Derived Neurotrophic Factor and Receptor Tyrosine Kinase B in Postmortem Brain of Suicide Subjects. Arch Gen Psychiatry. 2003;60(8):804–15. doi: 10.1001/archpsyc.60.8.804 12912764

[62] Isidro Ferrer M, PhD, Conxita Marín, MD, PhD, Ma Jesús Rey, MD, Teresa Ribalta, MD, PhD, Esther Goutan, BSc, Rosa Blanco, et al. BDNF and Full-length and Truncated TrkB Expression in Alzheimer Disease. Implications in Therapeutic Strategies. Journal of Neuropathology & Experimental Neurology. 1999;58(7):729–39. 10411343 10.1097/00005072-199907000-00007

[63] Carim-Todd L, Bath KG, Fulgenzi G, Yanpallewar S, Jing D, Barrick CA, et al. Endogenous truncated TrkB.T1 receptor regulates neuronal complexity and TrkB kinase receptor function in vivo. J Neurosci. 2009;29(3):678–85. doi: 10.1523/JNEUROSCI.5060-08.2009 19158294 PMC2719435

[64] Fenzl A, Kiefer FW. Brown adipose tissue and thermogenesis. Horm Mol Biol Clin Investig. 2014;19(1):25–37. doi: 10.1515/hmbci-2014-0022 25390014

[65] Auffret J, Viengchareun S, Carre N, Denis RG, Magnan C, Marie PY, et al. Beige differentiation of adipose depots in mice lacking prolactin receptor protects against high-fat-diet-induced obesity. FASEB J. 2012;26(9):3728–37. doi: 10.1096/fj.12-204958 22637534

[66] Lim S, Honek J, Xue Y, Seki T, Cao Z, Andersson P, et al. Cold-induced activation of brown adipose tissue and adipose angiogenesis in mice. Nat Protoc. 2012;7(3):606–15. doi: 10.1038/nprot.2012.013 22383039

[67] Wu J, Cohen P, Spiegelman BM. Adaptive thermogenesis in adipocytes: Is beige the new brown? Genes & Development. 2013;27(3):234–50. doi: 10.1101/gad.211649.112 23388824 PMC3576510

[68] McMurphy TH, Wei Queen, Nicholas J; Ali Seemaab; Widstrom Kyle J.;, Liu XX, et al. Implementation of environmental enrichment after middle age promotes healthy aging. Aging. 2018;10(7):1698–721. doi: 10.18632/aging.101502 30036185 PMC6075449

[69] Yadav A, Kataria MA, Saini V, Yadav A. Role of leptin and adiponectin in insulin resistance. Clin Chim Acta. 2013;417:80–4. doi: 10.1016/j.cca.2012.12.007 23266767

[70] Queathem ED, Welly RJ, Clart LM, Rowles CC, Timmons H, Fitzgerald M, et al. White Adipose Tissue Depots Respond to Chronic Beta-3 Adrenergic Receptor Activation in a Sexually Dimorphic and Depot Divergent Manner. Cells. 2021;10(12). 10.3390/cells10123453 PMC8700379 34943961

[71] Bachman ES, Dhillon H, Zhang C., Cinti S., Bianco A.C., Kobilka B.K., et al. betaAR signaling required for diet-induced thermogenesis and obesity resistance. Science. 2002;297(5582):843–5. doi: 10.1126/science.1073160 12161655

[72] Fruhbeck G, Mendez-Gimenez L, Fernandez-Formoso JA, Fernandez S, Rodriguez A. Regulation of adipocyte lipolysis. Nutr Res Rev. 2014;27(1):63–93. doi: 10.1017/S095442241400002X 24872083

[73] Puigserver P, Wu Z., Park C.W., Graves R., Wright M., Spiegelman B.M. A Cold-Inducible Coactivator of Nuclear Receptors Linked to Adaptive Thermogenesis. Cell. 1998;92(6):829–39. 9529258 10.1016/s0092-8674(00)81410-5

[74] Seale P, Bjork B, Yang W, Kajimura S, Chin S, Kuang S, et al. PRDM16 controls a brown fat/skeletal muscle switch. Nature. 2008;454(7207):961–7. doi: 10.1038/nature07182 18719582 PMC2583329

[75] Wang H, Wang B, Yin H, Zhang G, Yu L, Kong X, et al. Reduced neurotrophic factor level is the early event before the functional neuronal deficiency in high-fat diet induced obese mice. Metab Brain Dis. 2017;32(1):247–57. doi: 10.1007/s11011-016-9905-z 27624843

[76] Woo J SK, Park SY, Jang KS, Kang S. Effects of exercise and diet change on cognition function and synaptic plasticity in high fat diet induced obese rats. Lipids Health Dis. 2013;12(144). doi: 10.1186/1476-511X-12-144 24098984 PMC3851938

[77] Amodeo DA, Pahua AE, Zarate M, Taylor JA, Peterson S, Posadas R, et al. Differences in the expression of restricted repetitive behaviors in female and male BTBR T+tf/J mice. Behav Brain Res. 2019;372:112028. 31212059 10.1016/j.bbr.2019.112028

[78] Defensor EB, Pearson BL, Pobbe RL, Bolivar VJ, Blanchard DC, Blanchard RJ. A novel social proximity test suggests patterns of social avoidance and gaze aversion-like behavior in BTBR T+ tf/J mice. Behav Brain Res. 2011;217(2):302–8. doi: 10.1016/j.bbr.2010.10.033 21055421 PMC3124342

[79] Cai Y, Wang L, Xiao R, Li X, He X, Gao J, et al. Autism-like behavior in the BTBR mouse model of autism is improved by propofol. Neuropharmacology. 2017;118:175–87. doi: 10.1016/j.neuropharm.2017.03.021 28341205

[80] Reynolds S, Urruela M, Devine DP. Effects of environmental enrichment on repetitive behaviors in the BTBR T+tf/J mouse model of autism. Autism Res. 2013;6(5):337–43. doi: 10.1002/aur.1298 23813950 PMC4158697

[81] Yang M, Perry K, Weber MD, Katz AM, Crawley JN. Social peers rescue autism-relevant sociability deficits in adolescent mice. Autism Res. 2011;4(1):17–27. doi: 10.1002/aur.163 20928844 PMC3065860

[82] Amodeo DA, Oliver B, Pahua A, Hitchcock K, Bykowski A, Tice D, et al. Serotonin 6 receptor blockade reduces repetitive behavior in the BTBR mouse model of autism spectrum disorder. Pharmacol Biochem Behav. 2021;200:173076. doi: 10.1016/j.pbb.2020.173076 33220385

[83] Yang Y, Wang JZ. From Structure to Behavior in Basolateral Amygdala-Hippocampus Circuits. Front Neural Circuits. 2017;11:86. doi: 10.3389/fncir.2017.00086 29163066 PMC5671506

[84] Janak PH, Tye KM. From circuits to behaviour in the amygdala. Nature. 2015;517(7534):284–92. doi: 10.1038/nature14188 25592533 PMC4565157

[85] Deng W, Li F, Ke H, Wang S, Li Z, Lv P, et al. Effect of metformin in autistic BTBR T + Itpr3tf/J mice administered a high-fat diet. Brain Res Bull. 2022;183:172–83. 35240246 10.1016/j.brainresbull.2022.02.021

[86] Hwang SR, Kim CY, Shin KM, Jo JH, Kim HA, Heo Y. Altered expression levels of neurodevelopmental proteins in fetal brains of BTBR T+tf/J mice with autism-like behavioral characteristics. J Toxicol Environ Health A. 2015;78(8):516–23. doi: 10.1080/15287394.2015.1010466 25849768

[87] Werling DM, Geschwind DH. Sex differences in autism spectrum disorders. Curr Opin Neurol. 2013;26(2):146–53. doi: 10.1097/WCO.0b013e32835ee548 23406909 PMC4164392

