# Supplementary material for: Hypothalamic TrkB.FL overexpression improves metabolic outcomes in the BTBR mouse model of autism
Source: PLoS One. 2023 Mar 9;18(3):e0282566. doi: 10.1371/journal.pone.0282566 (PMC9997972; doi:10.1371/journal.pone.0282566)
Supplement: S1 Raw images — (PDF) [file pone.0282566.s004.pdf]

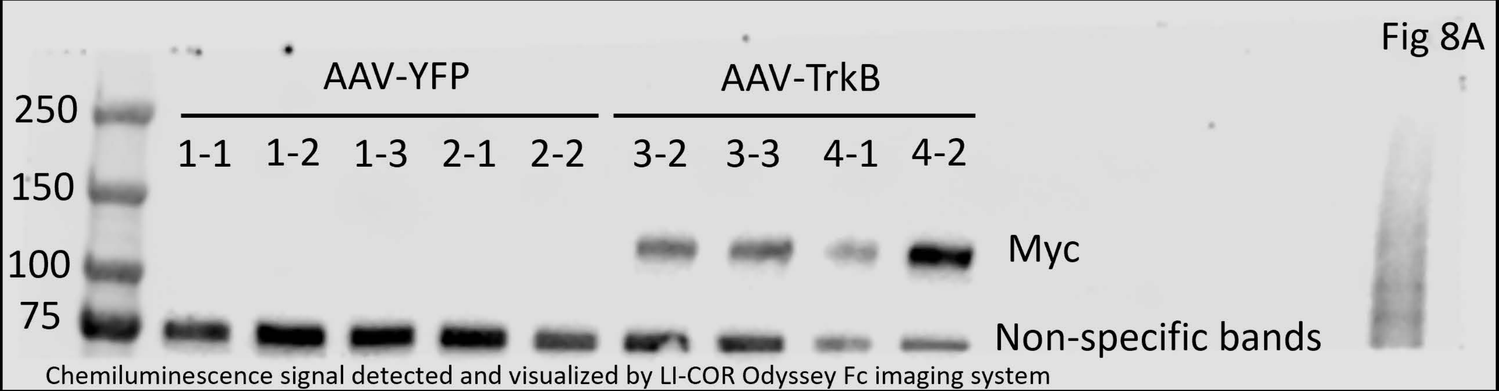
Fig 8A
AAV-YFP
AAV-TrkB
250
150
100
75
1-1
1-2
1-3
2-1
2-2
3-2
3-3
4-1
4-2
Myc
Non-specific bands
Chemiluminescence signal detected and visualized by LI-COR Odyssey Fc imaging system

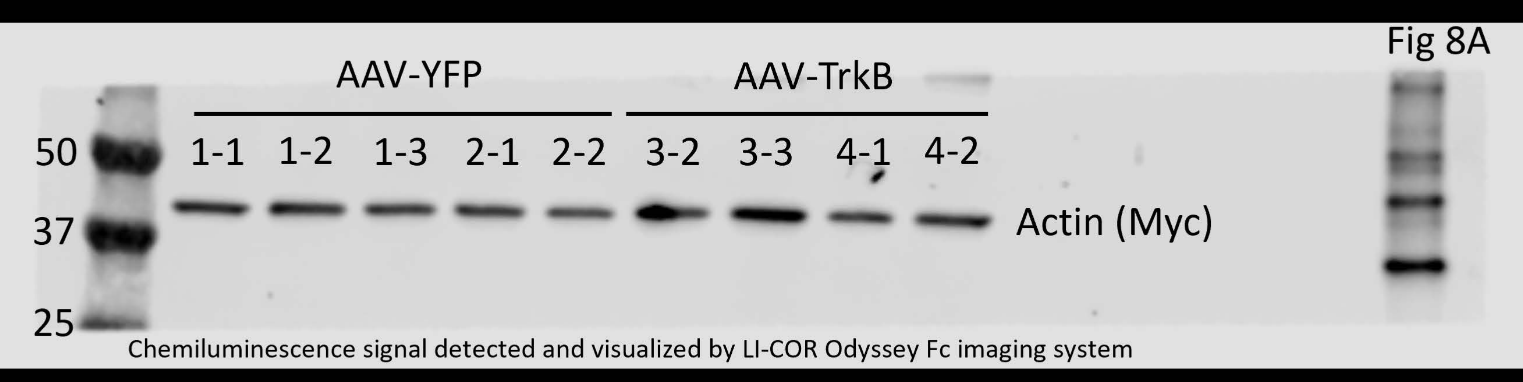
Fig 8A
AAV-YFP
AAV-TrkB
50
37
25
1-1
1-2
1-3
2-1
2-2
3-2
3-3
4-1
4-2
Actin (Myc)
Chemiluminescence signal detected and visualized by LI-COR Odyssey Fc imaging system

Fig 8A

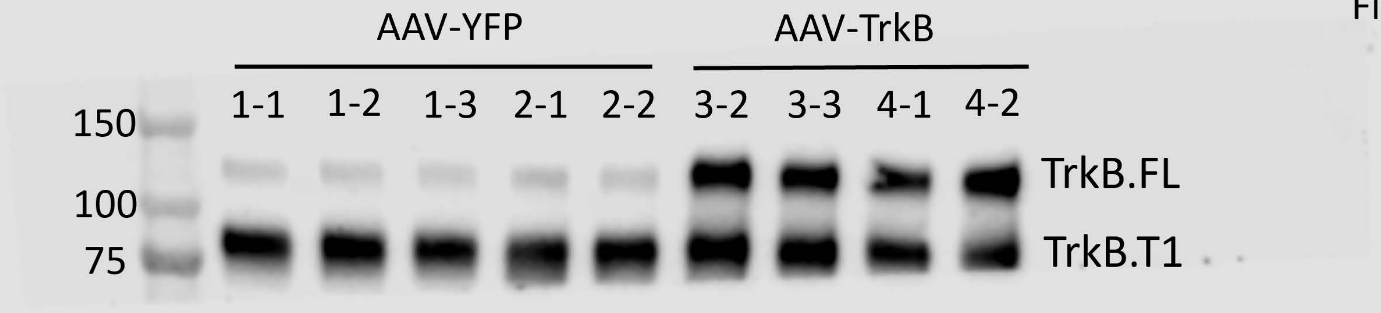

Chemiluminescence signal detected and visualized by LI-COR Odyssey Fc imaging system

Fig 8A

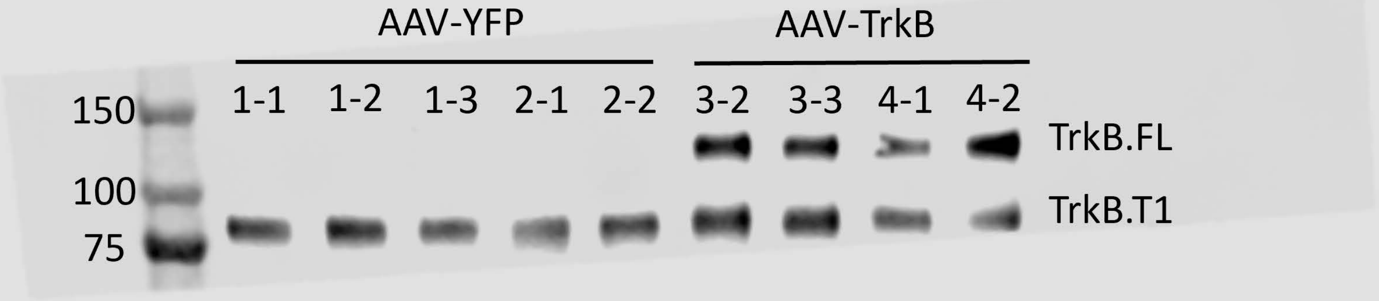

Chemiluminescence signal detected and visualized by LI-COR Odyssey Fc imaging system

Fig 8A

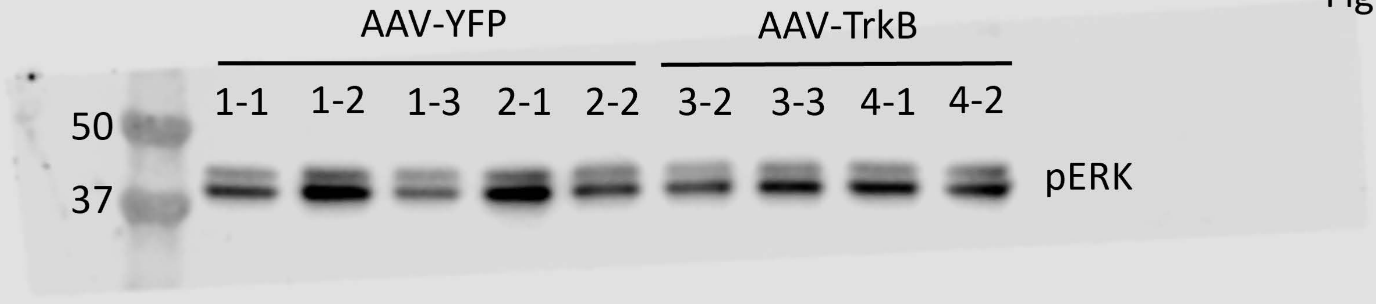

Chemiluminescence signal detected and visualized by LI-COR Odyssey Fc imaging system

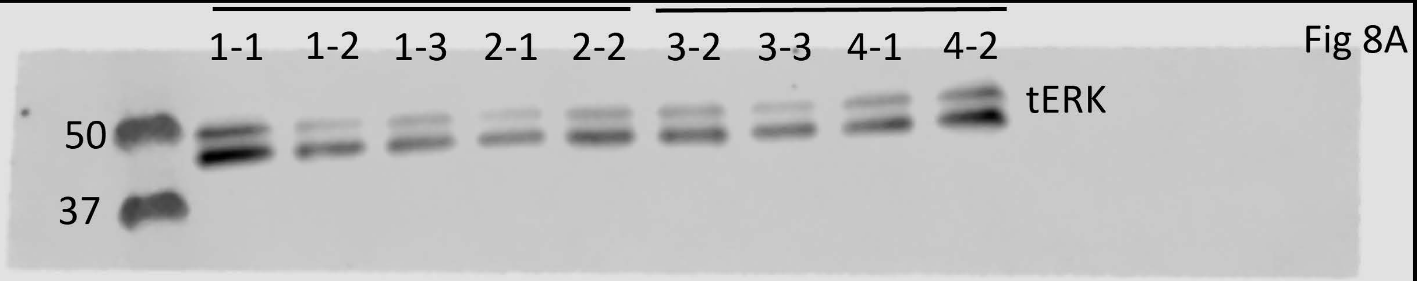

Chemiluminescence signal detected and visualized by LI-COR Odyssey Fc imaging system

Fig 8A

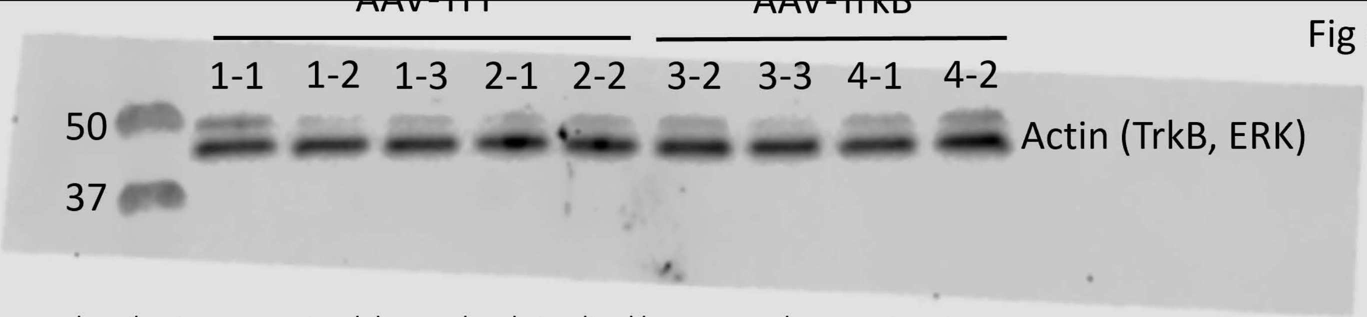

Chemiluminescence signal detected and visualized by LI-COR Odyssey Fc imaging system

Fig 8A

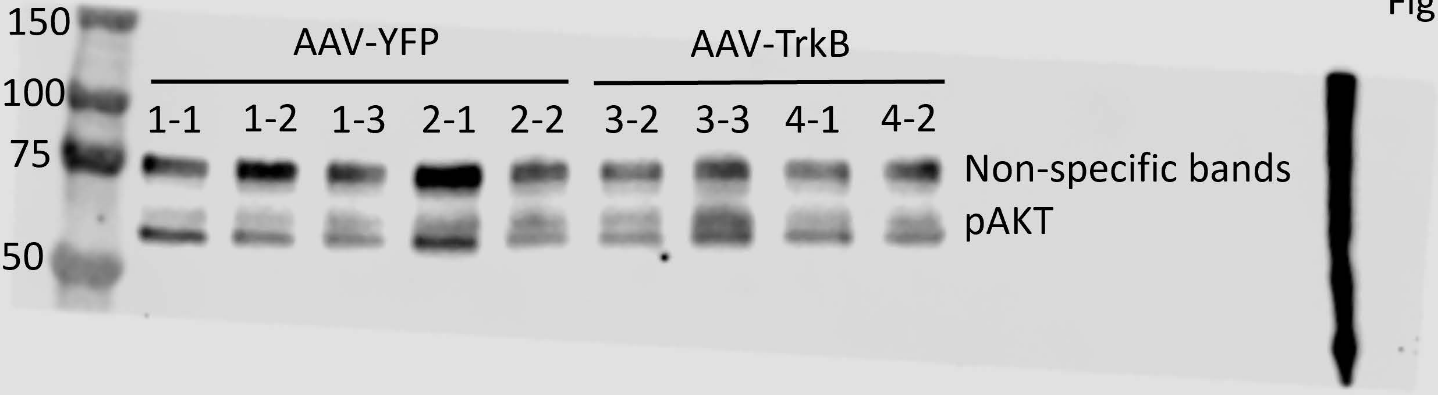

Chemiluminescence signal detected and visualized by LI-COR Odyssey Fc imaging system

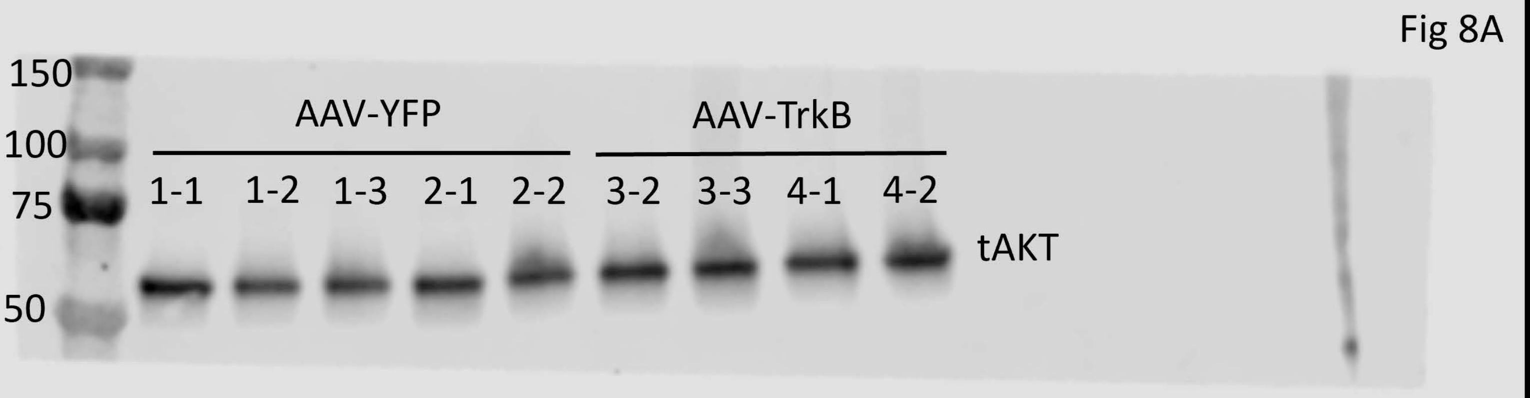

Chemiluminescence signal detected and visualized by LI-COR Odyssey Fc imaging system

Fig 8A

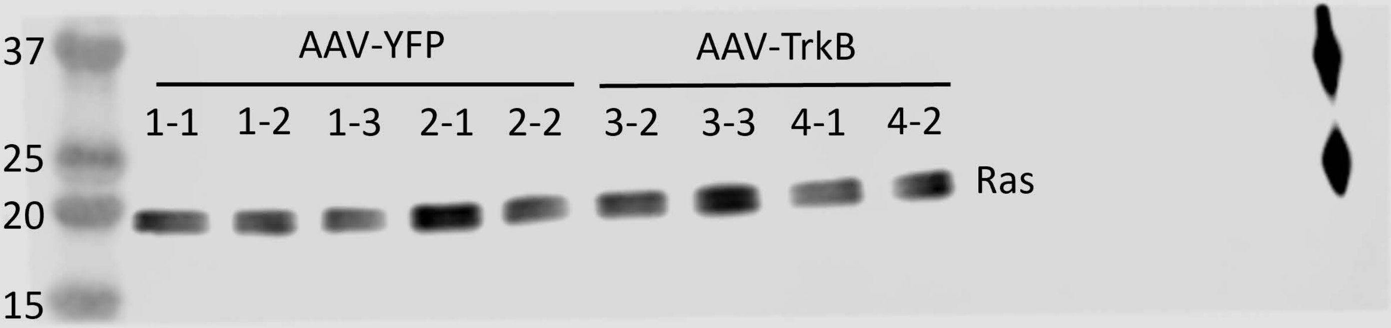

Chemiluminescence signal detected and visualized by LI-COR Odyssey Fc imaging system

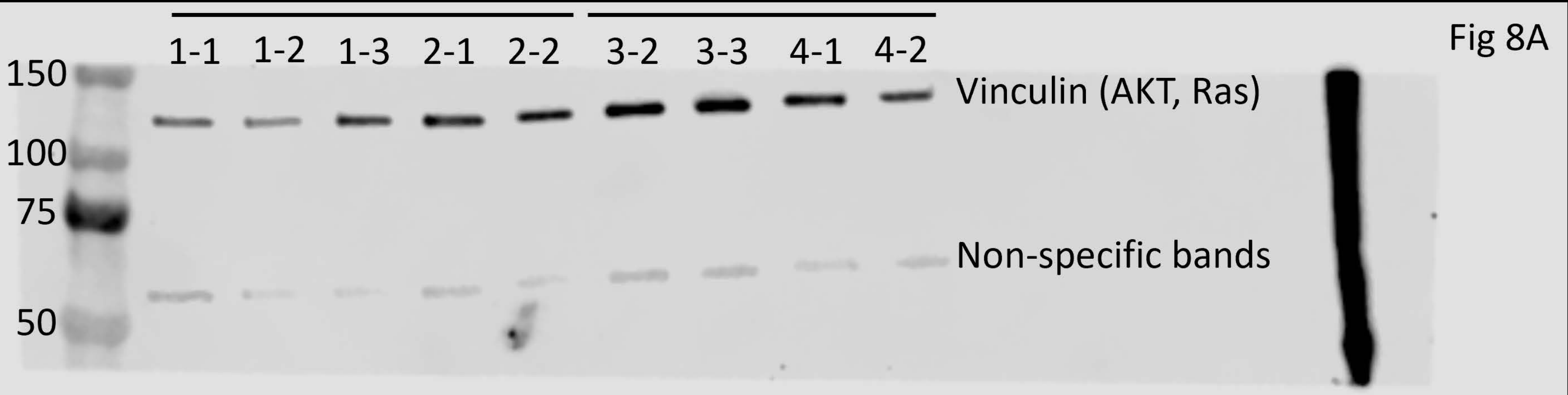

Chemiluminescence signal detected and visualized by LI-COR Odyssey Fc imaging system

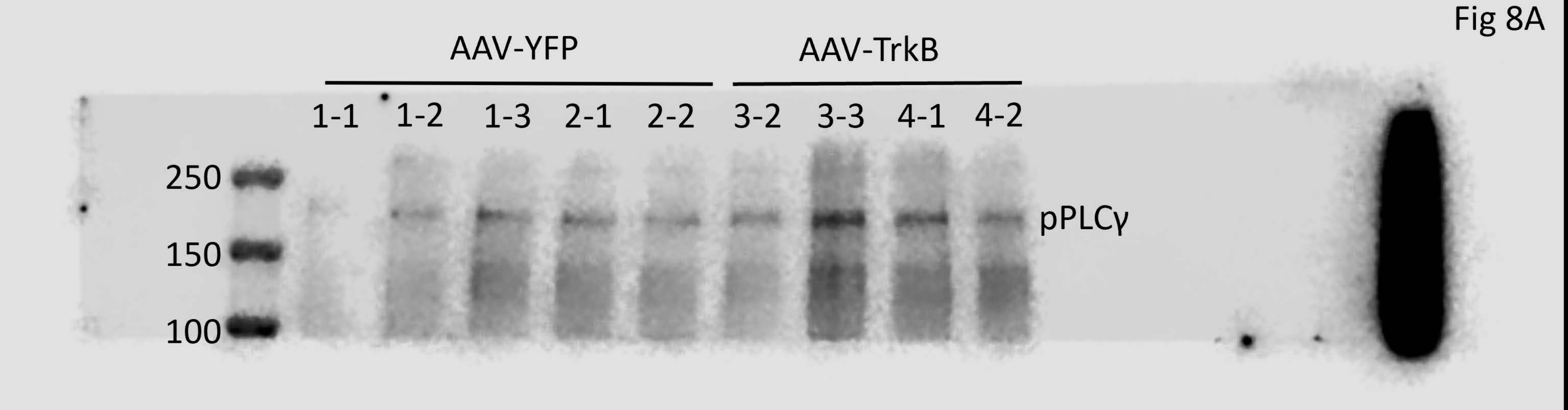

Chemiluminescence signal detected and visualized by LI-COR Odyssey Fc imaging system

Fig 8A

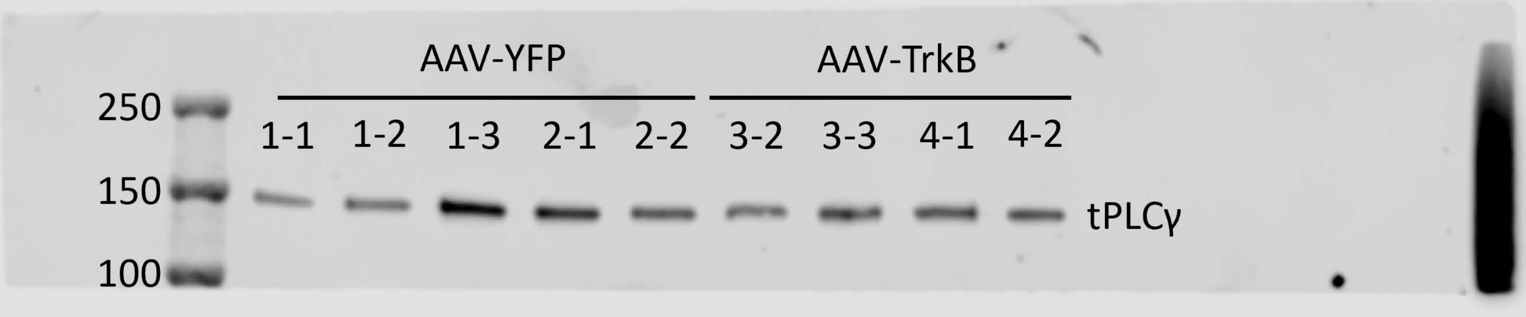

Chemiluminescence signal detected and visualized by LI-COR Odyssey Fc imaging system

Fig 8A

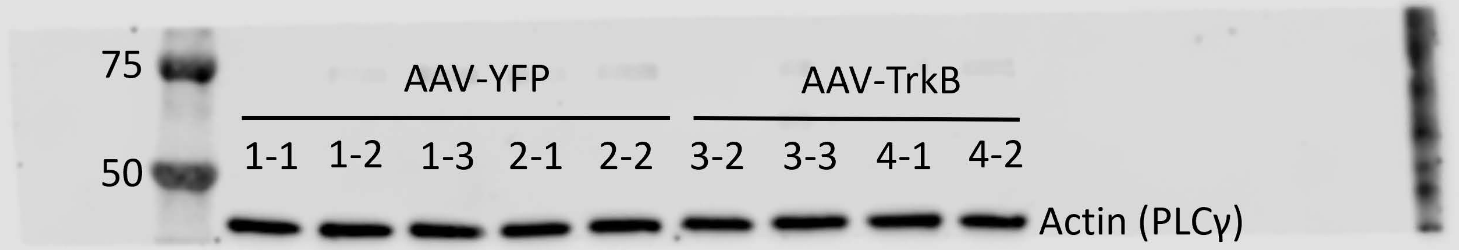

Chemiluminescence signal detected and visualized by LI-COR Odyssey Fc imaging system

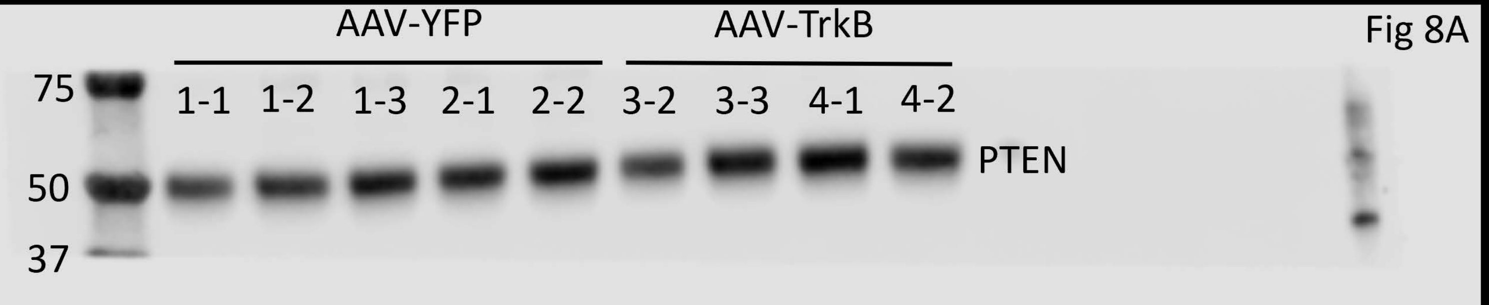

Chemiluminescence signal detected and visualized by LI-COR Odyssey Fc imaging system

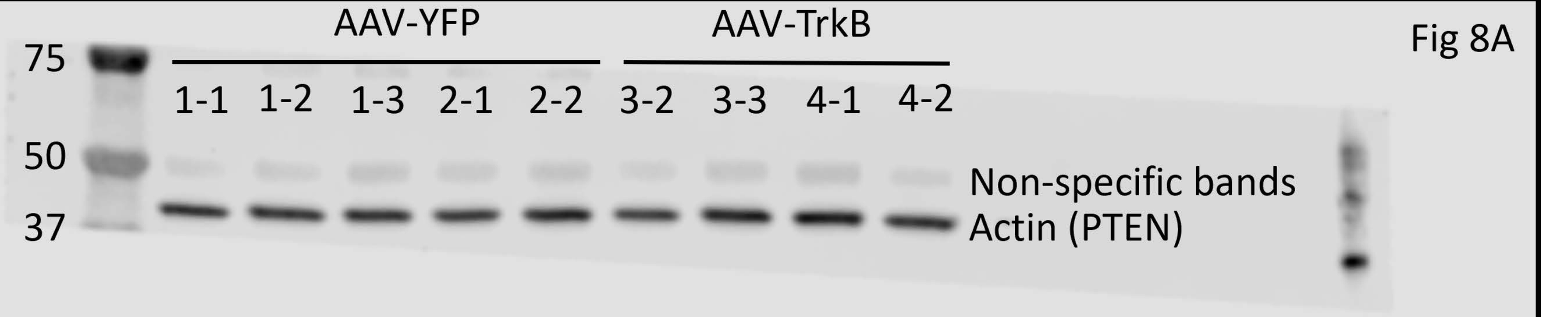

Chemiluminescence signal detected and visualized by LI-COR Odyssey Fc imaging system

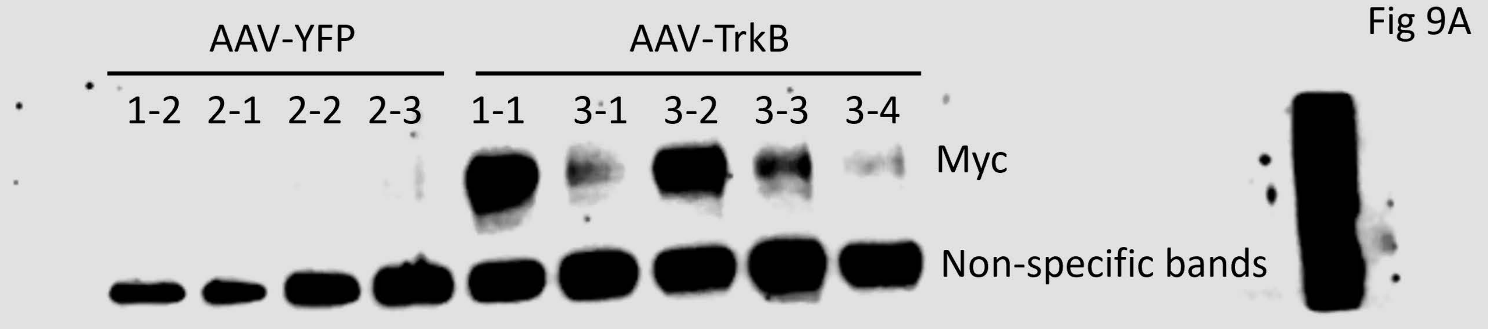

Chemiluminescence signal detected and visualized by LI-COR Odyssey Fc imaging system

Fig 9A

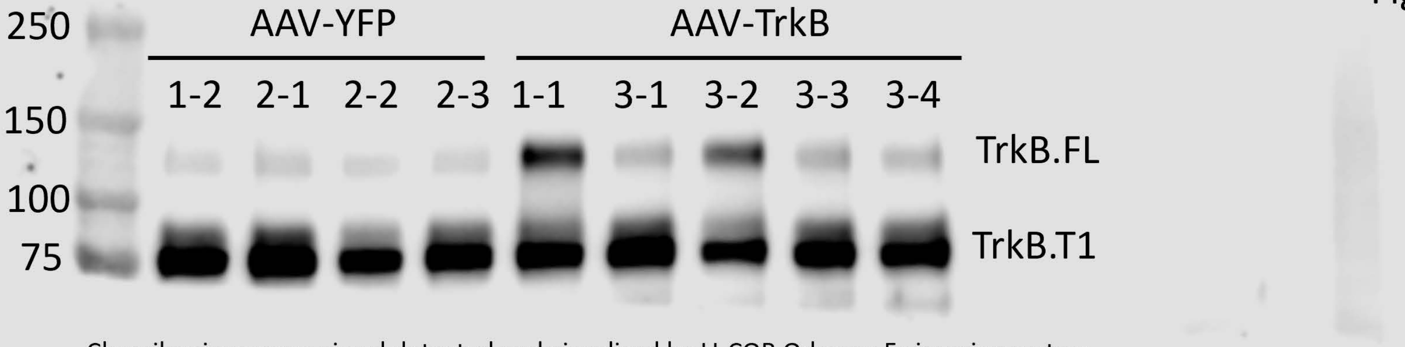

Chemiluminescence signal detected and visualized by LI-COR Odyssey Fc imaging system

Fig 9A

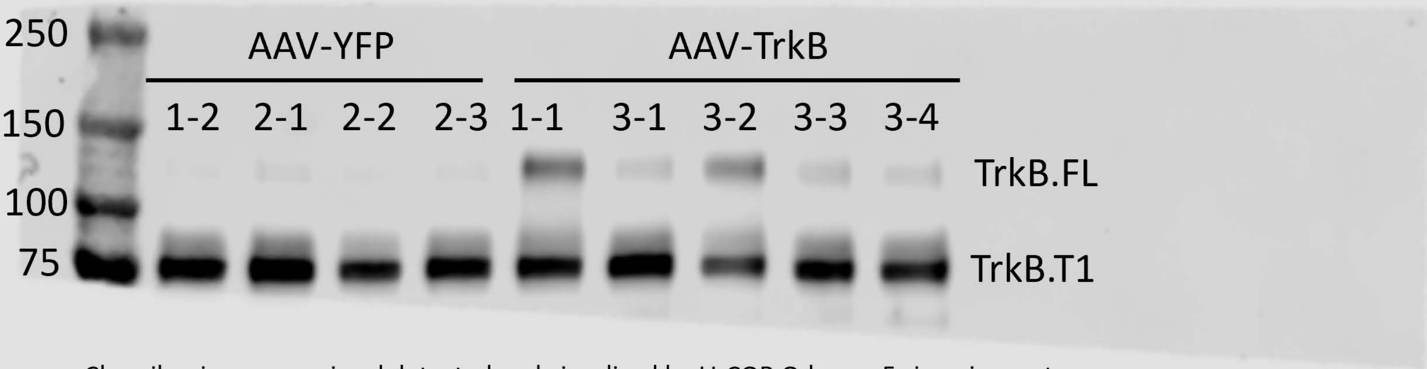

Chemiluminescence signal detected and visualized by LI-COR Odyssey Fc imaging system

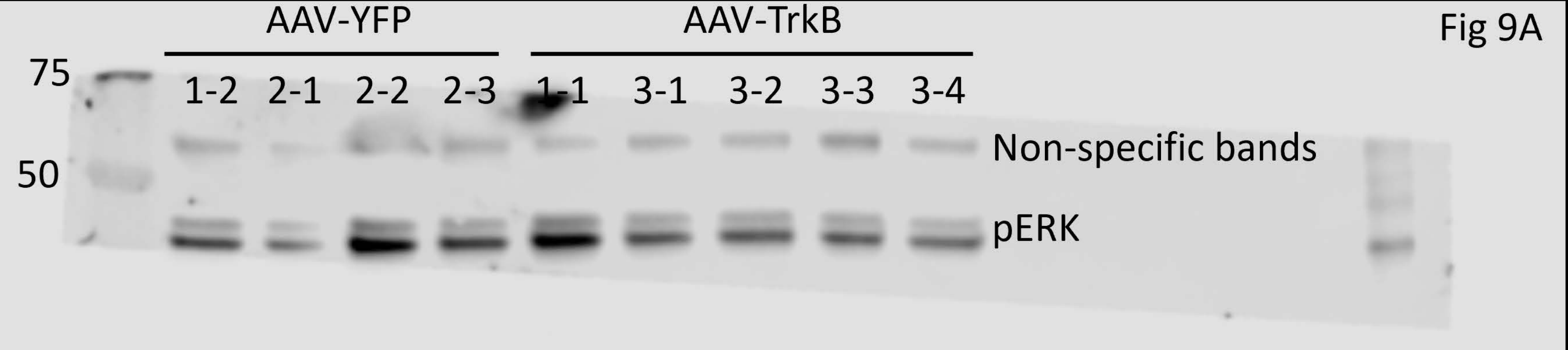

Chemiluminescence signal detected and visualized by LI-COR Odyssey Fc imaging system

Fig 9A

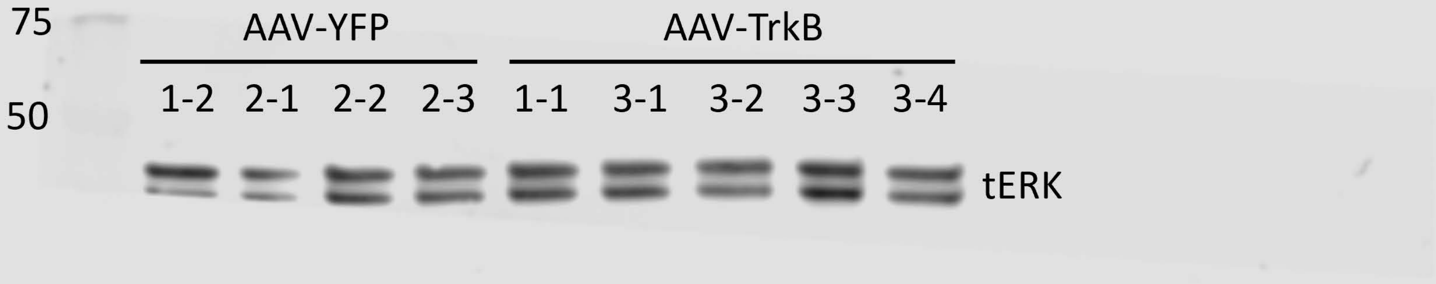

Chemiluminescence signal detected and visualized by LI-COR Odyssey Fc imaging system

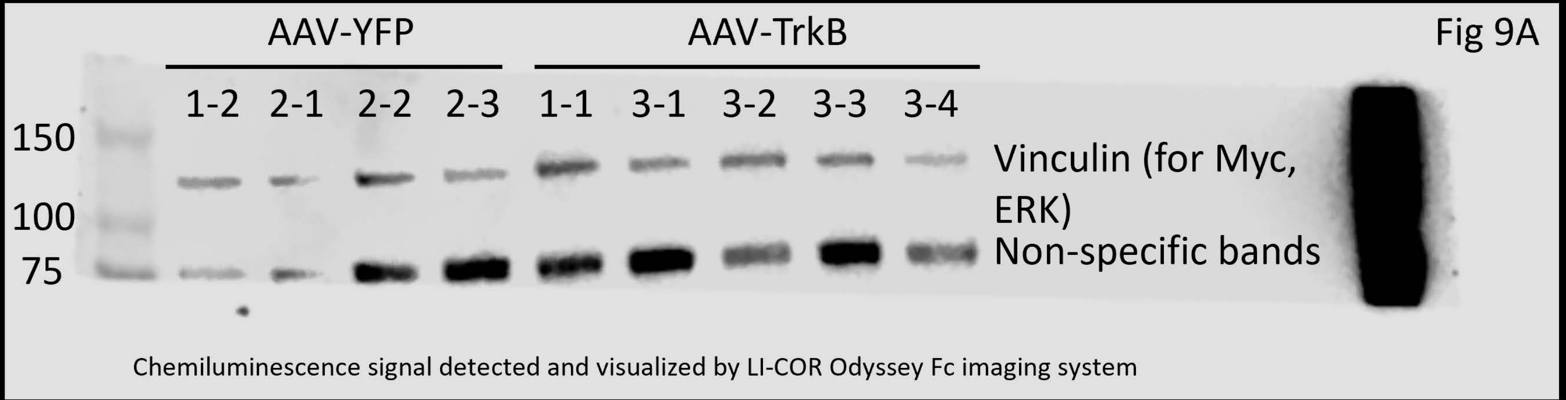
Fig 9A
AAV-YFP
AAV-TrkB
1-2
2-1
2-2
2-3
1-1
3-1
3-2
3-3
3-4
150
100
75
Vinculin (for Myc, ERK)
Non-specific bands
Chemiluminescence signal detected and visualized by LI-COR Odyssey Fc imaging system

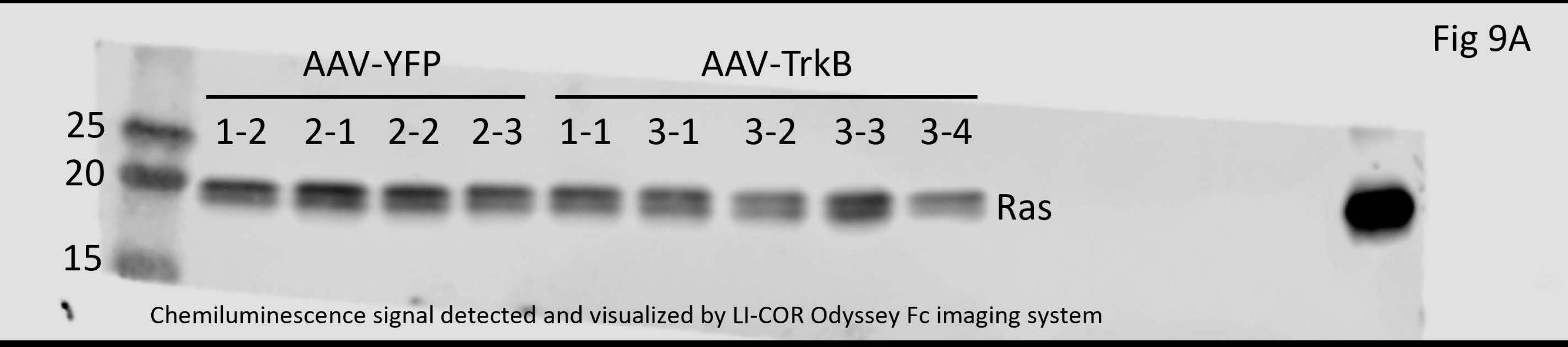
Fig 9A
AAV-YFP
AAV-TrkB
25
20
15
1-2
2-1
2-2
2-3
1-1
3-1
3-2
3-3
3-4
Ras
Chemiluminescence signal detected and visualized by LI-COR Odyssey Fc imaging system

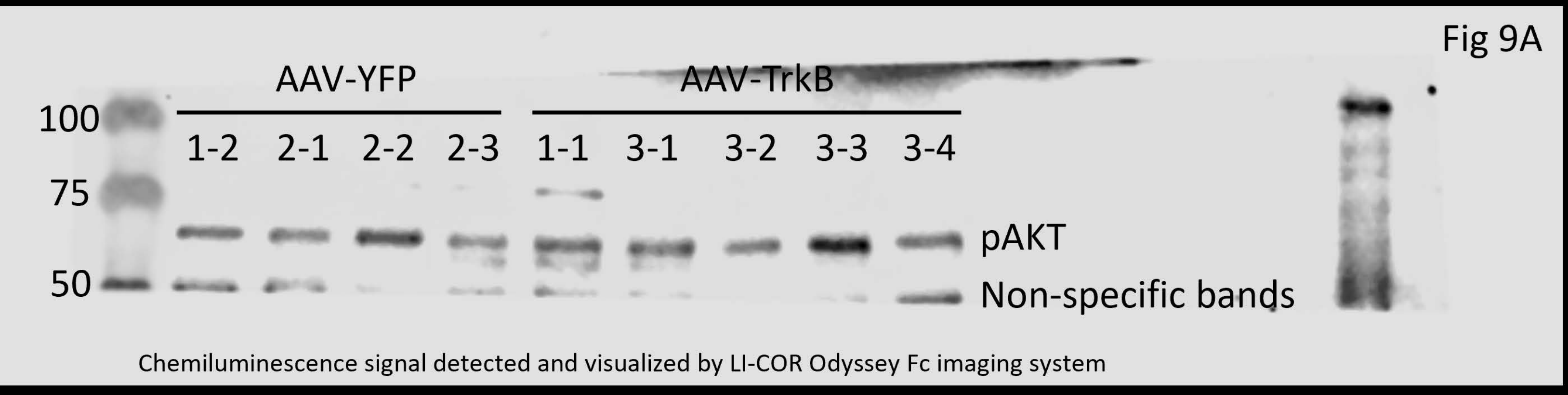
Fig 9A
AAV-YFP
AAV-TrkB
100
75
50
1-2
2-1
2-2
2-3
1-1
3-1
3-2
3-3
3-4
pAKT
Non-specific bands
Chemiluminescence signal detected and visualized by LI-COR Odyssey Fc imaging system

Fig 9A

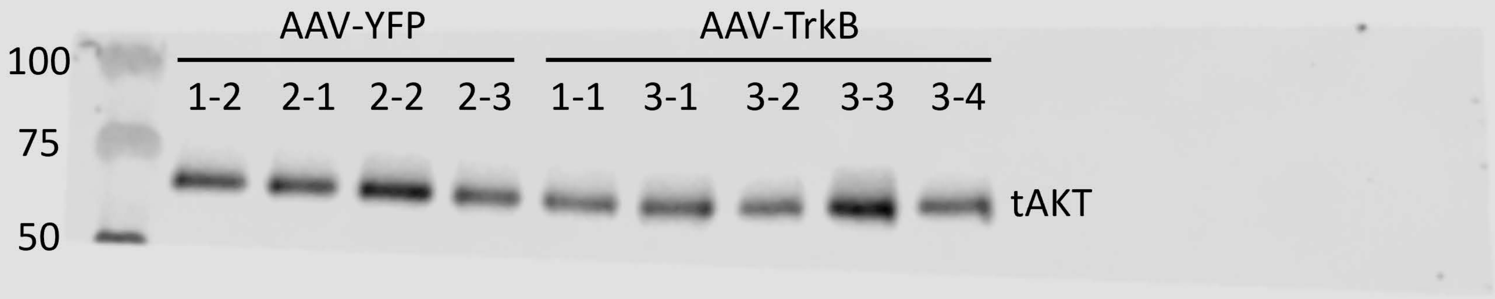

Chemiluminescence signal detected and visualized by LI-COR Odyssey Fc imaging system

Fig 9A

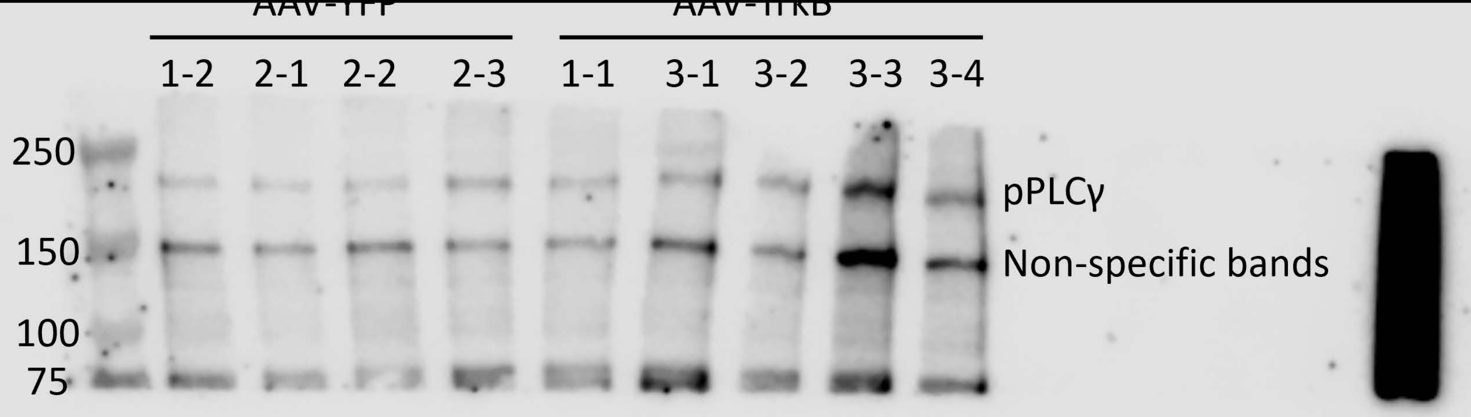

Chemiluminescence signal detected and visualized by LI-COR Odyssey Fc imaging system

[redacted]

Fig 9A

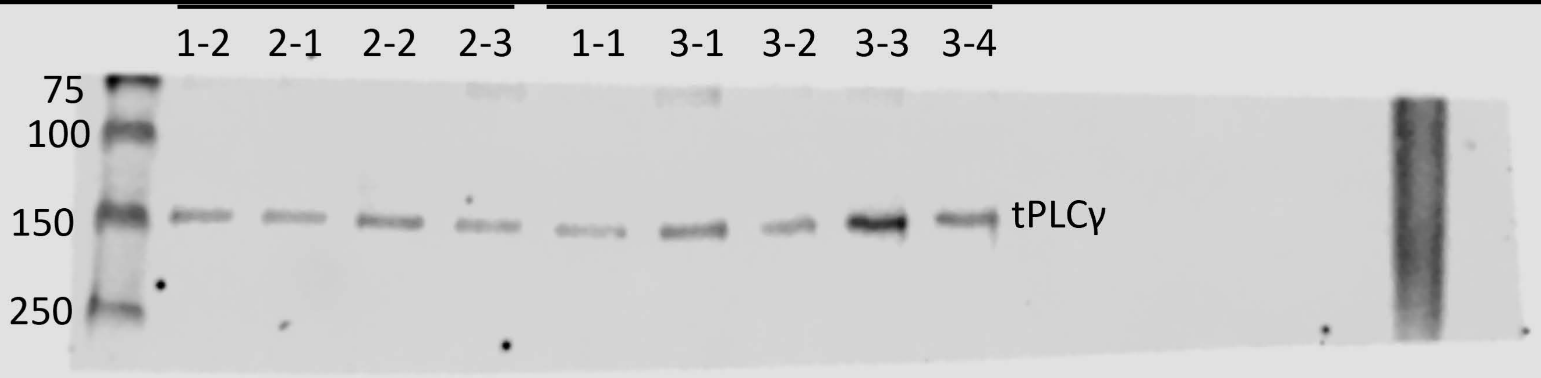

Chemiluminescence signal detected and visualized by LI-COR Odyssey Fc imaging system

Fig 9A

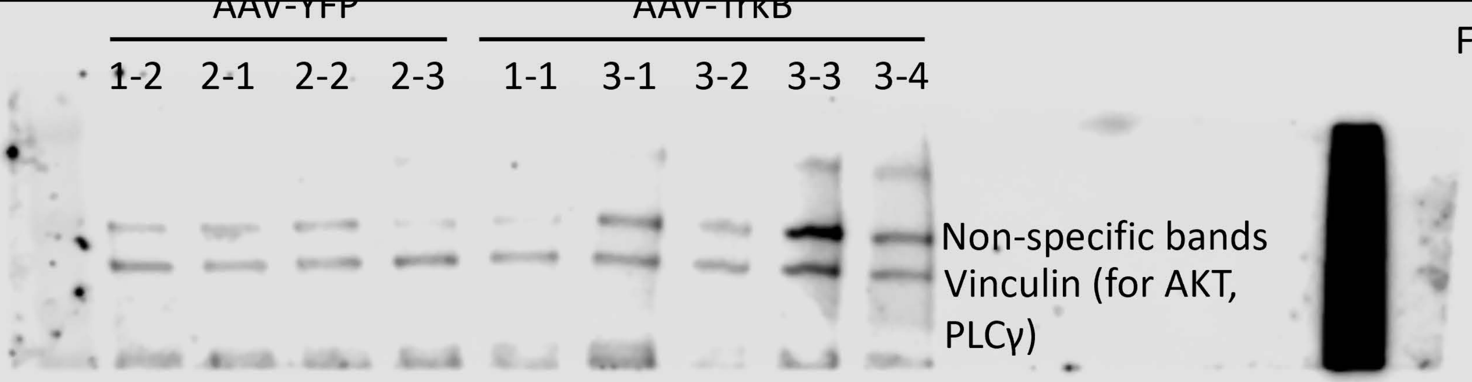

Chemiluminescence signal detected and visualized by LI-COR Odyssey Fc imaging system

Fig 9A

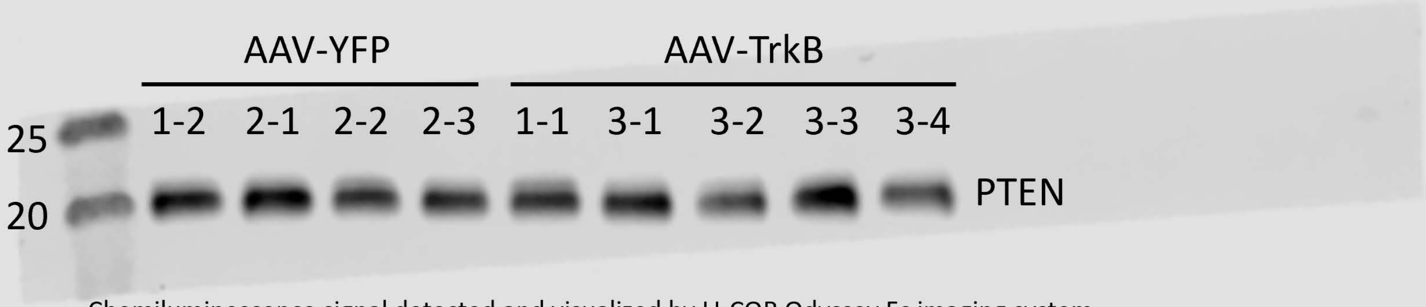

Chemiluminescence signal detected and visualized by LI-COR Odyssey Fc imaging system

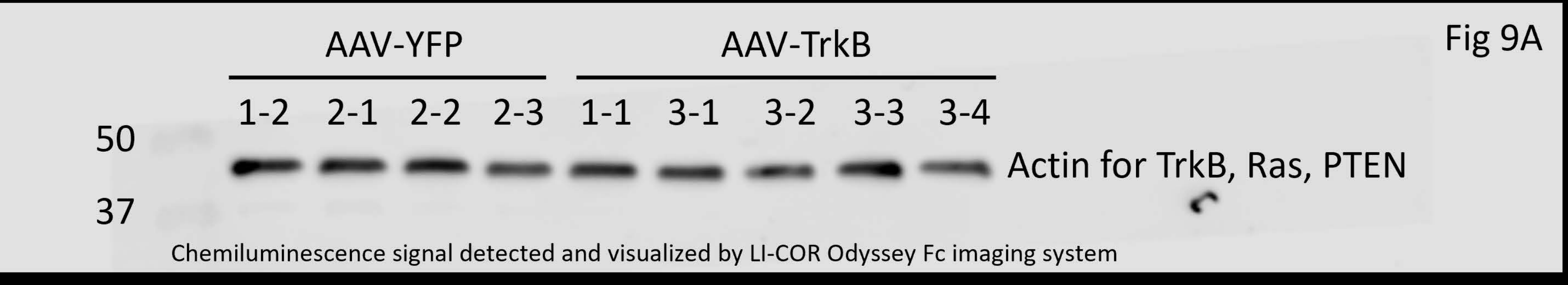
Fig 9A
AAV-YFP
AAV-TrkB
1-2
2-1
2-2
2-3
1-1
3-1
3-2
3-3
3-4
50
37
Actin for TrkB, Ras, PTEN
Chemiluminescence signal detected and visualized by LI-COR Odyssey Fc imaging system

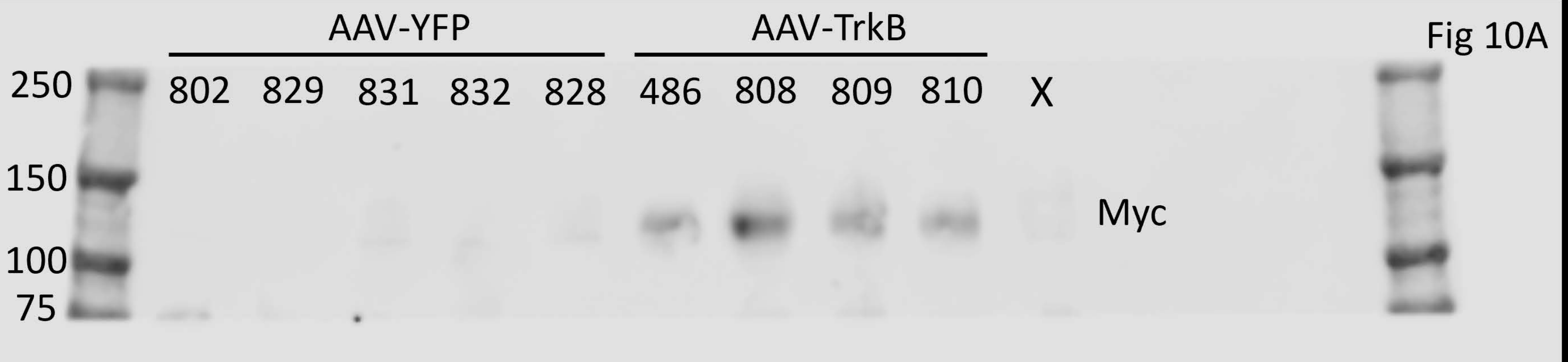

Chemiluminescence signal detected and visualized by LI-COR Odyssey Fc imaging system

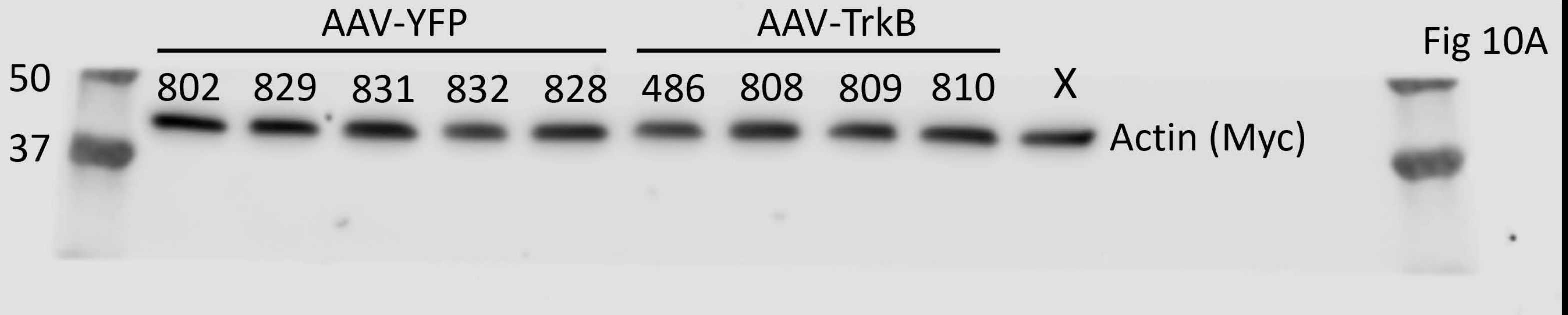

Chemiluminescence signal detected and visualized by LI-COR Odyssey Fc imaging system

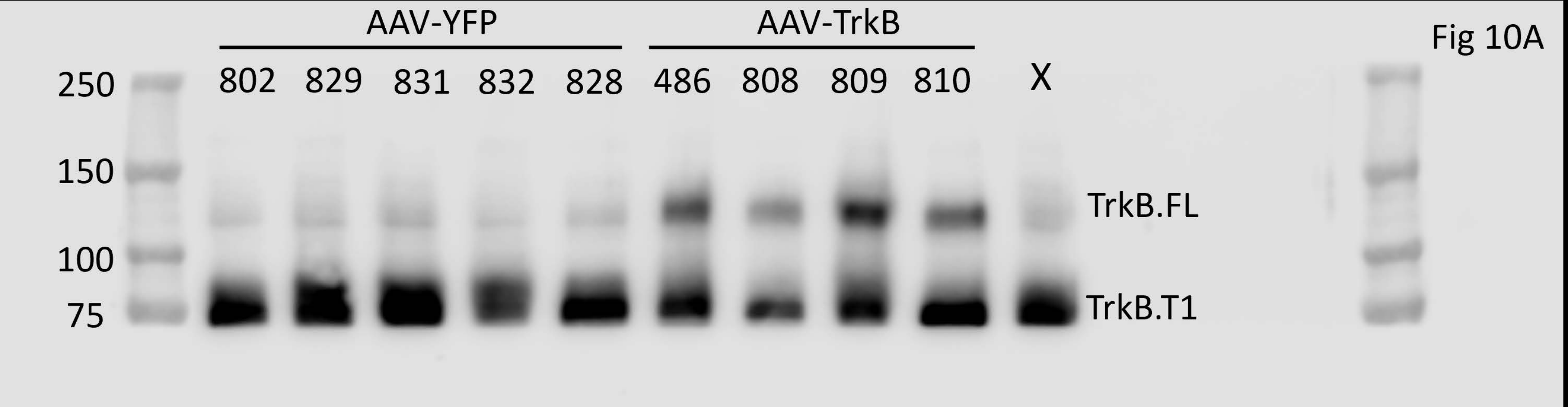

Chemiluminescence signal detected and visualized by LI-COR Odyssey Fc imaging system

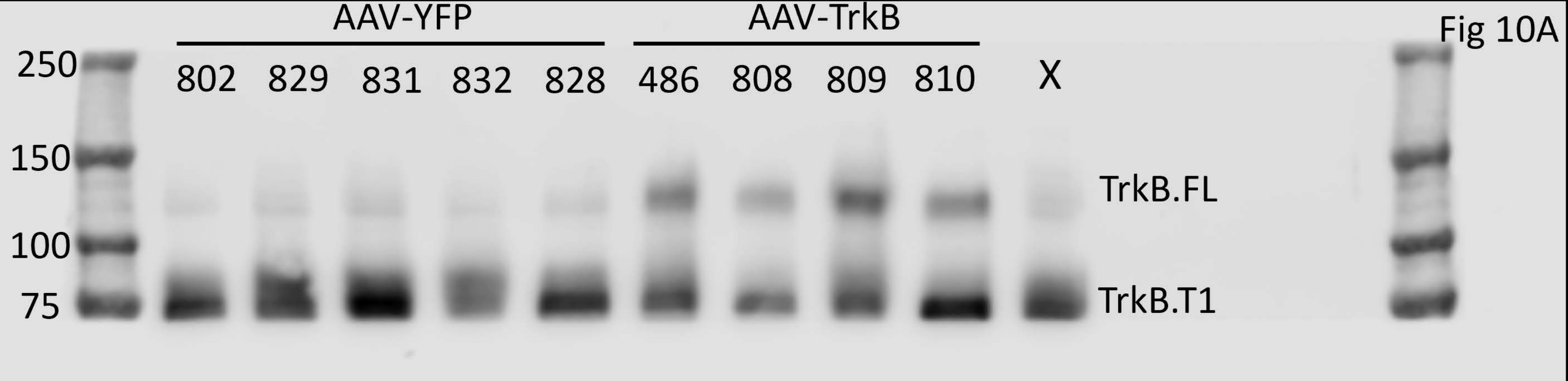

Chemiluminescence signal detected and visualized by LI-COR Odyssey Fc imaging system

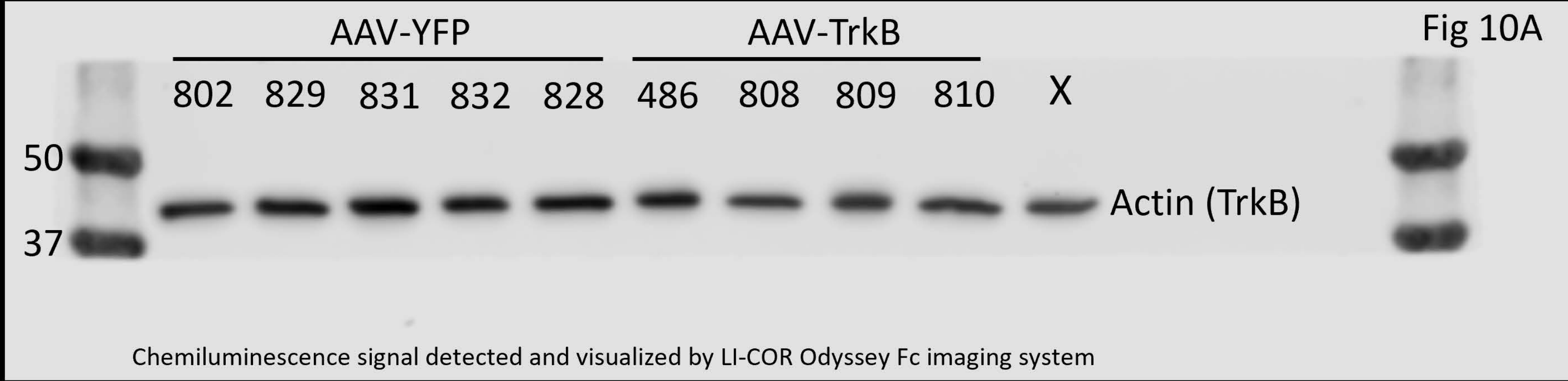

Fig 10A
AAV-YFP
AAV-TrkB
802
829
831
832
828
486
808
809
810
X
50
37
Actin (TrkB)
Chemiluminescence signal detected and visualized by LI-COR Odyssey Fc imaging system

Fig 10A

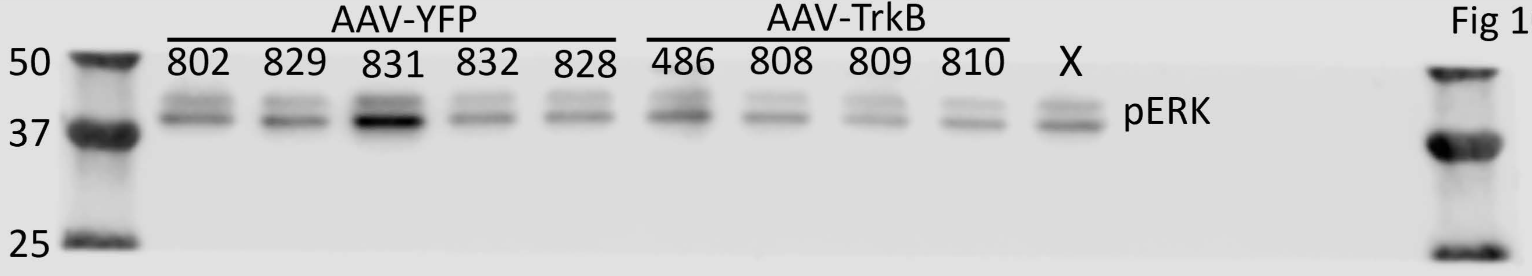

Chemiluminescence signal detected and visualized by LI-COR Odyssey Fc imaging system

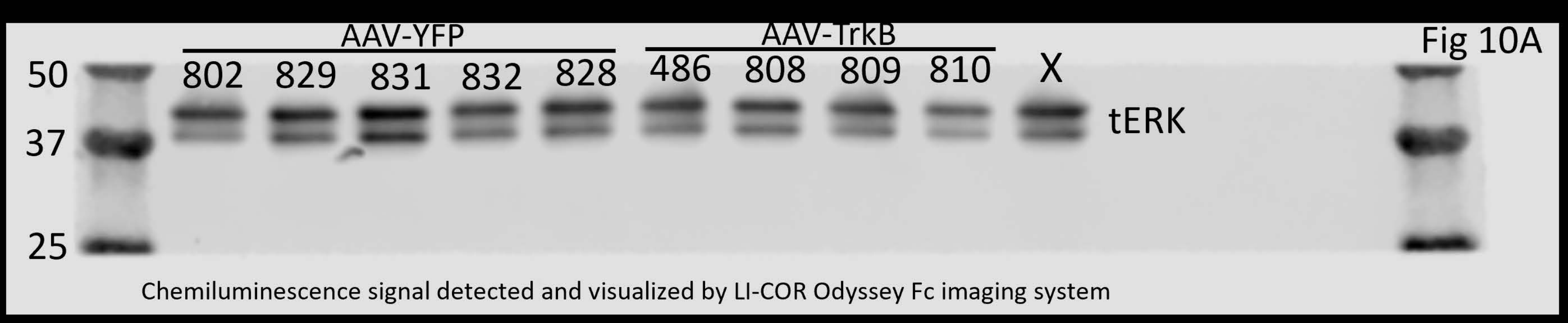

Fig 10A
AAV-YFP
AAV-TrkB
802
829
831
832
828
486
808
809
810
X
50
37
25
tERK
Chemiluminescence signal detected and visualized by LI-COR Odyssey Fc imaging system

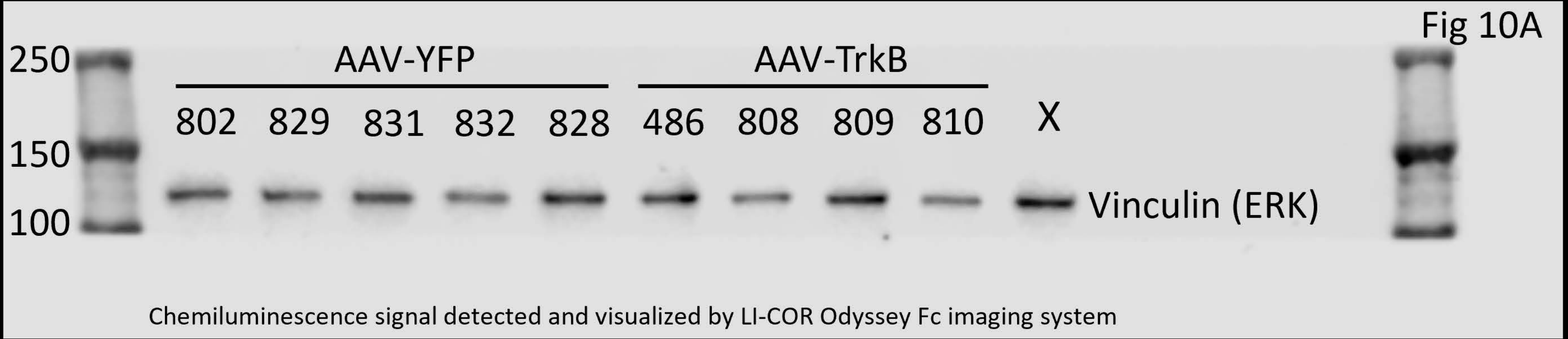
Fig 10A
250
150
100
AAV-YFP
AAV-TrkB
802
829
831
832
828
486
808
809
810
X
Vinculin (ERK)
Chemiluminescence signal detected and visualized by LI-COR Odyssey Fc imaging system

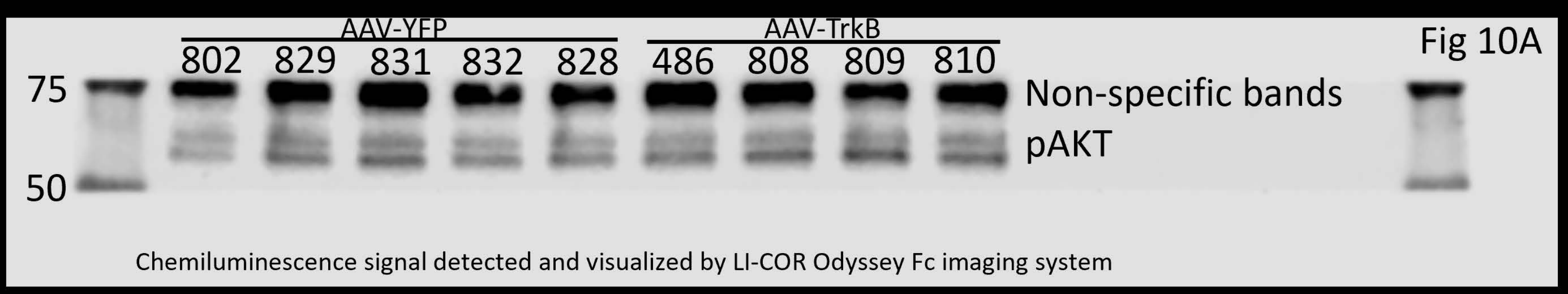
AAV-YFP
AAV-TrkB
802
829
831
832
828
486
808
809
810
75
50
Non-specific bands
pAKT
Fig 10A
Chemiluminescence signal detected and visualized by LI-COR Odyssey Fc imaging system

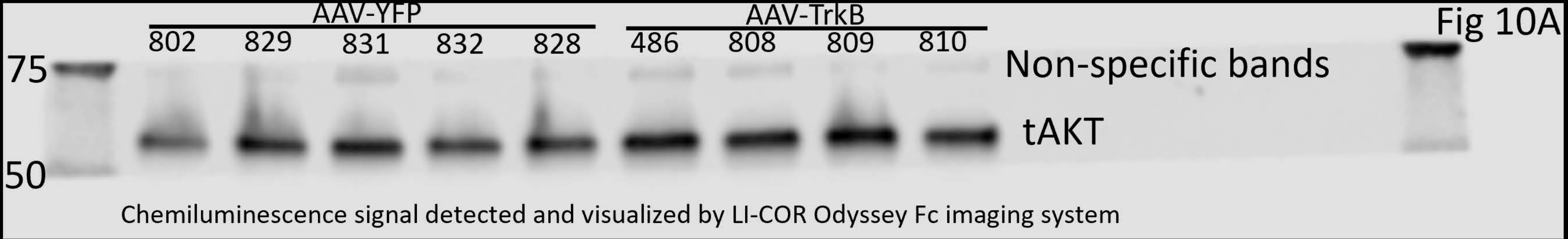
AAV-YFP
AAV-TrkB
Fig 10A
802
829
831
832
828
486
808
809
810
75
Non-specific bands
tAKT
50
Chemiluminescence signal detected and visualized by LI-COR Odyssey Fc imaging system

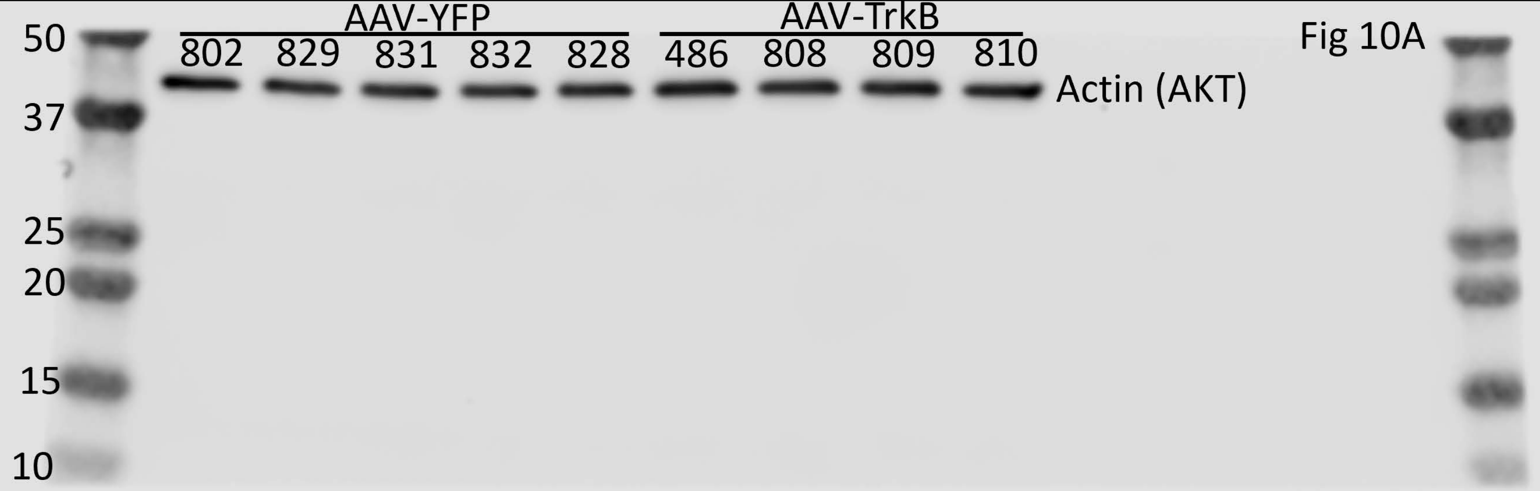

Chemiluminescence signal detected and visualized by LI-COR Odyssey Fc imaging system

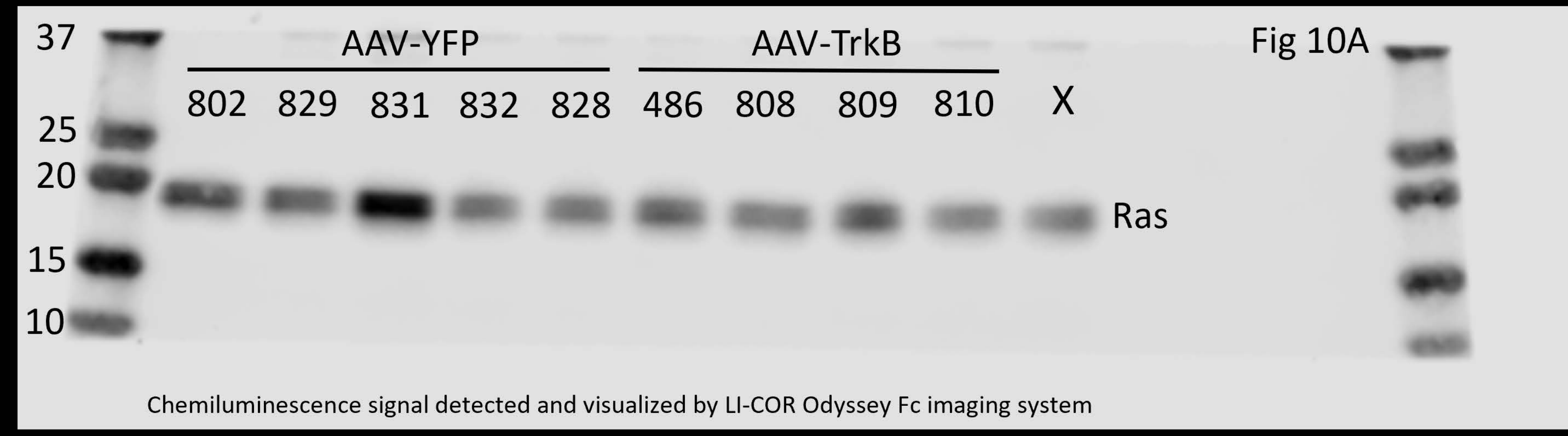
37
AAV-YFP
AAV-TrkB
Fig 10A
802
829
831
832
828
486
808
809
810
X
25
20
Ras
15
10
Chemiluminescence signal detected and visualized by LI-COR Odyssey Fc imaging system

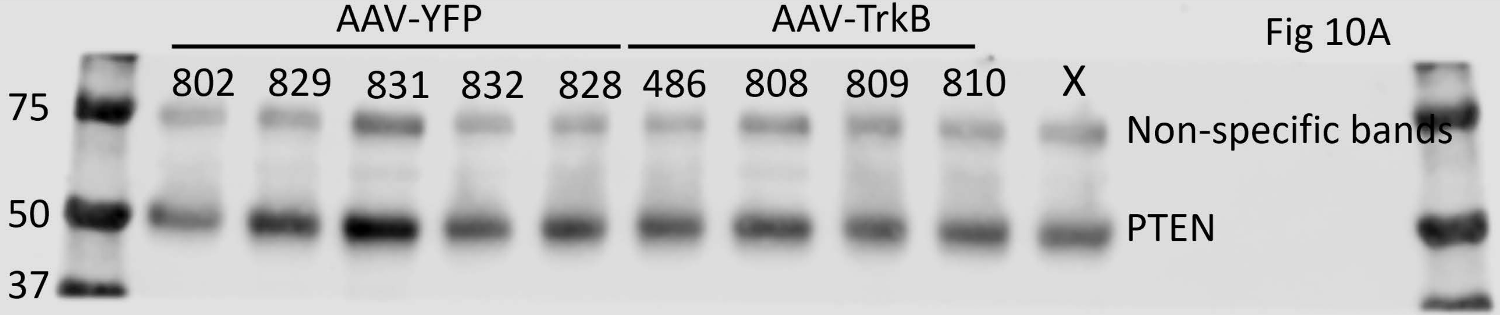

Chemiluminescence signal detected and visualized by LI-COR Odyssey Fc imaging system

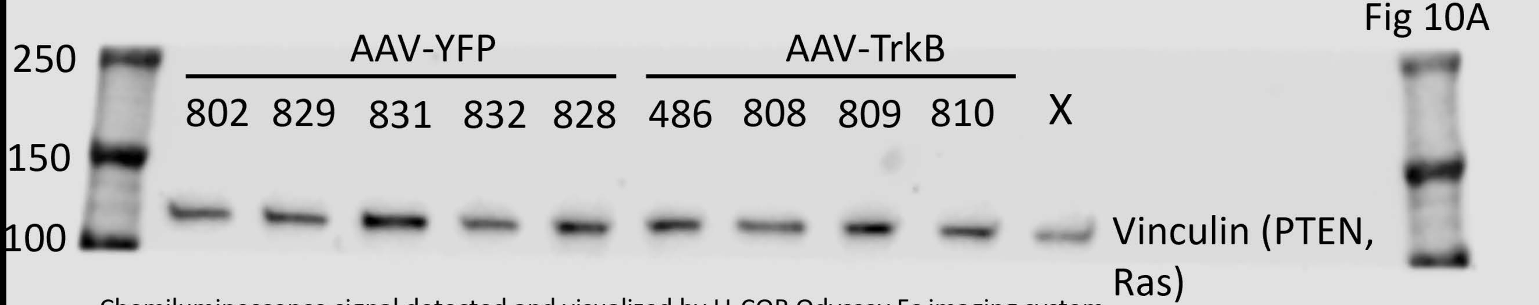

Chemiluminescence signal detected and visualized by LI-COR Odyssey Fc imaging system

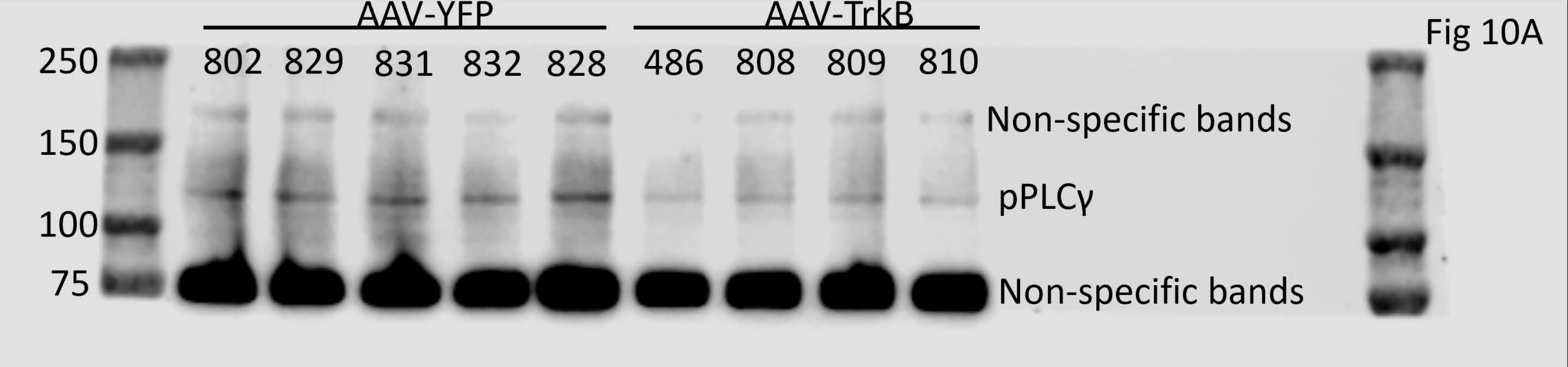

Chemiluminescence signal detected and visualized by LI-COR Odyssey Fc imaging system

Fig 10A

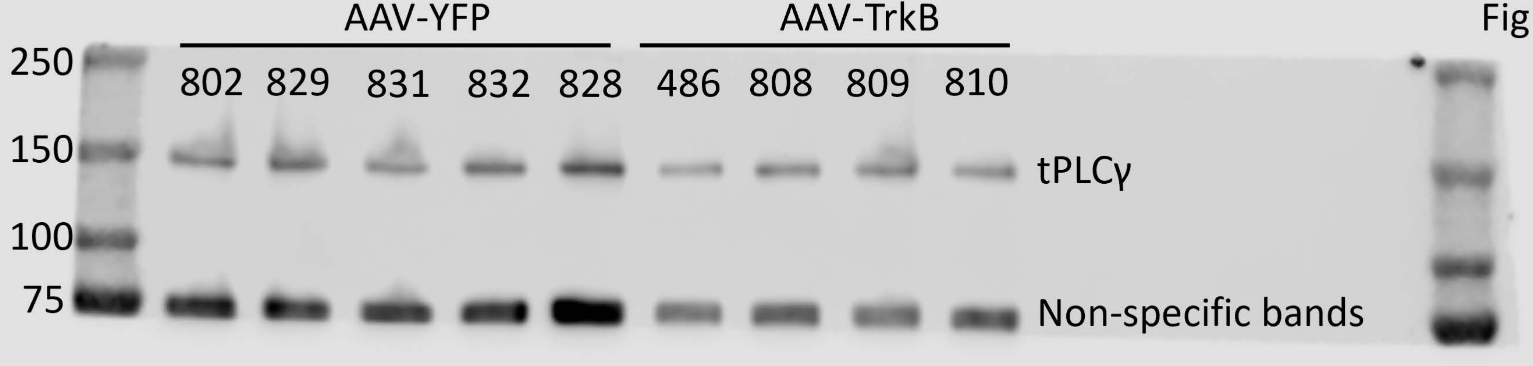

Chemiluminescence signal detected and visualized by LI-COR Odyssey Fc imaging system

Fig 10A

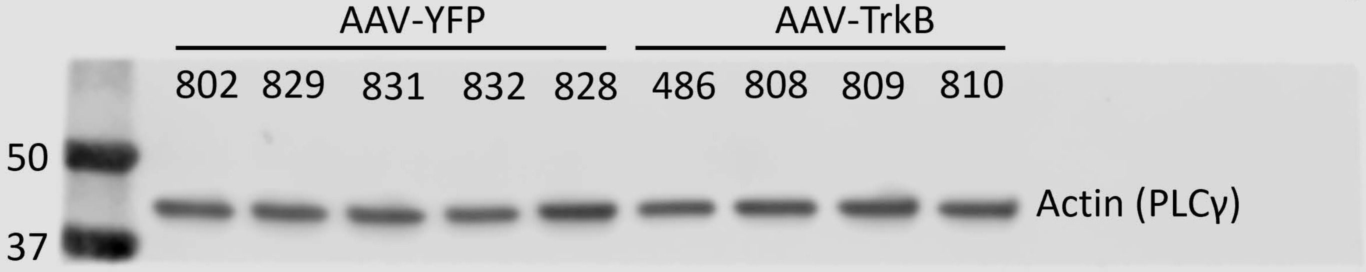

Chemiluminescence signal detected and visualized by LI-COR Odyssey Fc imaging system
