# Supplementary material for: Hypothalamic TrkB.FL overexpression improves metabolic outcomes in the BTBR mouse model of autism
Source: PLoS One. 2023 Mar 9;18(3):e0282566. doi: 10.1371/journal.pone.0282566 (PMC9997972; doi:10.1371/journal.pone.0282566)
Supplement: S1 Text — (DOCX) [file pone.0282566.s005.docx]

**Supplementary Materials**

**Supplementary Text 1: Behavioral Methods**

**Open Field Test**

Mice were subjected to an open field test (OFT) at 12 wpi (long-term NCD) and 22 wpi (long-term HFD). Mice were individually placed in the center of an open square arena (60 cm x 60 cm, enclosed by opaque walls 48 cm in height) and allowed to explore for 10 minutes. During this time, time spent and distance traveled in both the center and periphery of the arena were recorded and scored by TopScan software (Clever Sys, Inc.). The arena was cleaned with OptiCide between each trial to remove odor cues.

**Cold Induced Defecation**

At 17 wpi, long-term HFD mice were individually placed in a clear plastic bin that was placed atop a larger bin filled with ice. Mice were allowed to explore for 10 minutes, after which the number of fecal boli was recorded. Bins were cleaned with OptiCide between mice to remove odor cues.

**Three Chamber Sociability Test**

At 18 wpi, long-term HFD mice were individually placed in a three chambered Plexiglass apparatus with removable chamber dividers. Mice were allowed to habituate in the closed center chamber for 5 minutes. In the first phase, which measures social affiliation, an unfamiliar mouse was placed in a small wire cage in either the right or left chamber, while another wire cage in the opposite chamber was left empty. The cage dividers were removed, and the test subject was allowed to explore all chambers for 10 minutes. Immediately afterward, the second phase was conducted, which measures novel social engagement. The conspecific from the first phase (now the familiar mouse) was placed in one wire cage, and a new unfamiliar mouse was placed in the wire cage in the opposite chamber. The test subject was allowed to explore all three chambers for 10 minutes. All trials were video recorded and a blinded experimenter analyzed for the time spent in each chamber and number of chamber entries. Between each trial, the arena was cleaned with Opticide to remove odor cues.

**Novelty Suppressed Feeding**

At 18 wpi, long-term HFD mice were fasted for 24 hours before testing. Mice were individually placed in a clean cage containing a pre-weighed normal chow food pellet placed on top of a filter paper. Mice were allowed to roam freely within the cage for 10 minutes, while the latency to eat the pellet was recorded. At the end of the trial the food pellet was weighed for amount of food consumed.
