# Supplementary material for: Hypothalamic TrkB.FL overexpression improves metabolic outcomes in the BTBR mouse model of autism
Source: PLoS One. 2023 Mar 9;18(3):e0282566. doi: 10.1371/journal.pone.0282566 (PMC9997972; doi:10.1371/journal.pone.0282566)
Supplement: S1 Table — (DOCX) [file pone.0282566.s006.docx]

**Supplementary Table 1.** Primer sequences used for qPCR.

| Gene | Sequence |
| --- | --- |
| *Actinb* | ACCCGCGAGCACAGCTT  ATATCGTCATCCATGGCGAACT |
| *Adipoq* | CCCTCCACCCAAGGGAACT  CCATTGTGGCCAGGATGTC |
| *Adrb3* | GGACGCTGTTCCTTTAAAAGCA  TCCATCTCACCCCCCATGT |
| *Apoe* | CCTGAACCGCTTCTGGGATT  CCATCAGTGCCGTCAGTTCT |
| *Bdnf* | CCATAAGGACGCGGACTTGT  AGGCTCCAAAGGCACTTGACT |
| *Ccl2* | GCTGTAGTTTTTGTCACCAAGC AAGGCATCACAGTCCGAGTC |
| *Cidea* | TGCTCTTCTGTATCGCCCAGT  GCCGTGTTAAGGAATCTGCTG |
| *Crh* | TGGCCCCAAGGAGGAAA CCACTGCAGCTCCAAATAAAAA |
| *Cx3cr1* | GCGACAAGATGACCTCACGA  TGTCGTCTCCAGGACAATGG |
| *Dio2* | GCACGTCTCCAATCCTGAAT  TGAACCAAAGTTGACCACCA |
| *Elovl3* | TCCGCGTTCTCATGTAGGTCT  GGACCTGATGCAACCCTATGA |
| *H2Ab1* | ACGGTGTGCAGACACAACTA CGACATTGGGCTGTTCAAGC |
| *Hsl* | GCGCCAGGACTGGAAAGAAT  TGAGAACGCTGAGGCTTTGAT |
| *Il1b* | GCCACCTTTTGACAGTGATGAG GGAAGCAGCCCTTCATCTTTT |
| *Il33* | ATGAAGCCTAAAATGAAGTA  CTAAGTTTCAGAGAGCTTAA |
| *Insr* | GGCTCTCCCCAGGAAACTACA GGTTCTGTCCAGGAGCCATTT |
| *Klb* | GGACACAACCTGATCAAGGCAC AAGGTGATGGAGAGCCAACC |
| *Lep* | ATTTCACACACGCAGTCGGTAT  AGCCCAGGAATGAAGTCCAA |
| *Mc4r* | CACTGTGTCAGGCGTCCTCTT  ATGGAAATGAGGCAGATGATGA |
| *Npy* | CTCCGCTCTGCGACACTACA AGTGTCTCAGGGCTGGATCTCT |
| *Obrb* | AATGACGCAGGGCTGTATGT TCAGGCTCCAGAAGAAGAGG |
| *Ppargc1a* | AAGTGTGGAACTCTCTGGAACTG  GGGTTATCTTGGTTGGCTTTATG |
| *Pomc* | GGCCTTTCCCCTAGAGTTCAA GGACCTGCTCCAAGCCTAATG |
| *Prdm16* | CAGCACGGTGAAGCCATTC  GCGTGCATCCGCTTGTG |
| *Pten* | TGGATTCGACTTAGACTTGACCT  GCGGTGTCATAATGTCTCTCAG |
| *Tnfa* | ACGGCATGGATCTCAAAGAC AGATAGCAAATCGGCTGACG |
| *TrkB-FL* | GACAATGCACGCAAGGACTT AGTAGTCGGTGCTGTACACA |
| *TrkB.T1* | AGCAATCGGGAGCATCTCT  TACCCATCCAGTGGGATCTT |
| *Ucp1* | CGATGTCCATGTACACCAAGGA  CCCGAGTCGCAGAAAAGAAG |
| *Vegfa* | TACCTCCACCATGCCAAGTG  CATGGGACTTCTGCTCTCCTTCT |
| *Hprt1* | TGTTGTTGGATATGCCCTTG GCGCTCATCTTAGGCTTTGT |
| *Vgf* | GGGCGCCCCGATGT  TCAGCTACCTGCCCATTATGC |
