# Supplementary material for: Hypothalamic TrkB.FL overexpression improves metabolic outcomes in the BTBR mouse model of autism
Source: PLoS One. 2023 Mar 9;18(3):e0282566. doi: 10.1371/journal.pone.0282566 (PMC9997972; doi:10.1371/journal.pone.0282566)
Supplement: S2 Table — (DOCX) [file pone.0282566.s007.docx]

**Supplementary Table 2.** Primary antibodies used for western blotting.

| **Antibody** | **Concentration** | **Catalogue Number** |
| --- | --- | --- |
| β-actin, mouse mAb | 1:500 | Cell Signaling #3700 |
| Vinculin, rabbit mAb | 1:500 | Cell Signaling #13901 |
| Phospho-AKT (S473), rabbit pAb | 1:500 | Cell Signaling #9271 |
| Total AKT, rabbit pAb | 1:1000 | Cell Signaling #9272 |
| Phospho-p44/42 MAPK (T202/Y204), rabbit pAb | 1:500 | Cell Signaling #9101 |
| Total p44/42 MAPK, rabbit pAb | 1:1000 | Cell Signaling #9102 |
| Ras, rabbit pAb | 1:500 | Cell Signaling #3965 |
| Phospho-PLCγ1 (Tyr783), rabbit mAb | 1:500 | Cell Signaling #14008 |
| Total PLCγ1, rabbit pAb | 1:1000 | Cell Signaling #2822 |
| TrkB, rabbit mAb | 1:1000 | Cell Signaling #4603 |
| Myc, mouse mAb | 1:250 | Cell Signaling #2276 |
| PTEN, rabbit mAb | 1:1000 | Cell Signaling #9559 |
