# Supplementary figures and images for: Hypothalamic TrkB.FL overexpression improves metabolic outcomes in the BTBR mouse model of autism
Source: PLoS One. 2023 Mar 9;18(3):e0282566. doi: 10.1371/journal.pone.0282566 (PMC9997972; doi:10.1371/journal.pone.0282566)

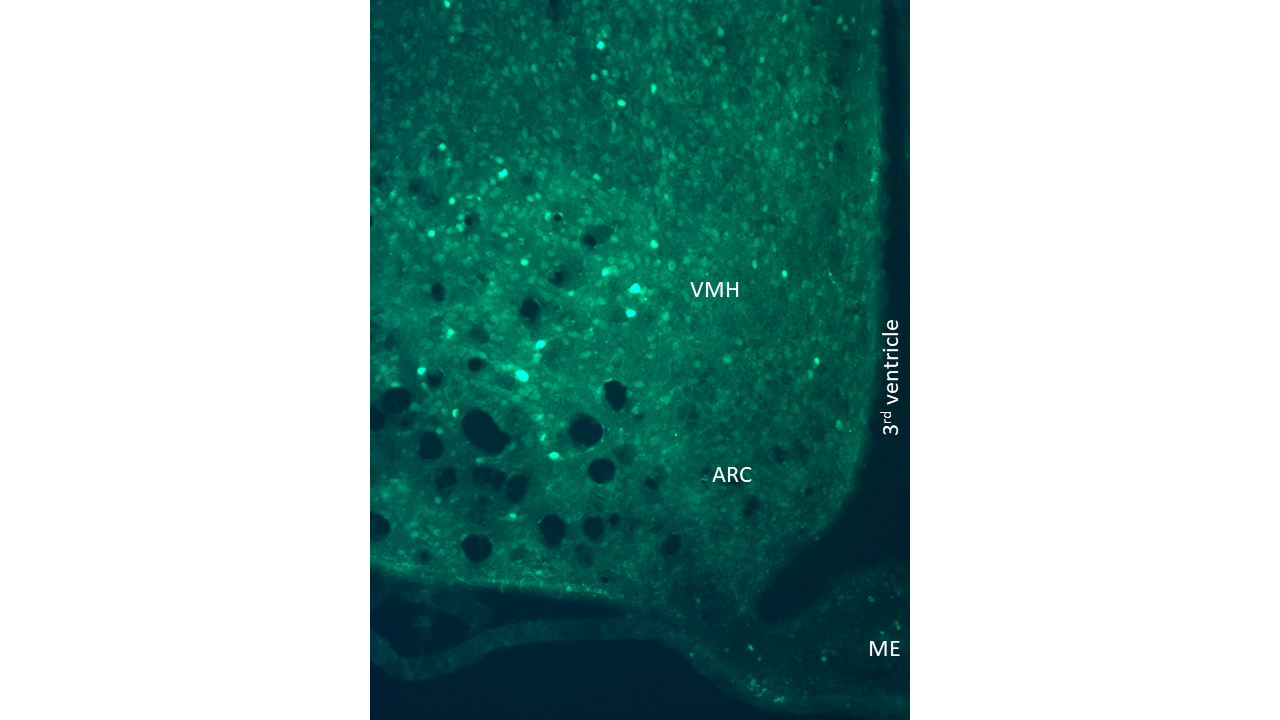

Supplement: S1 Fig — Representative image was taken at 10x magnification. ARC, arcuate nucleus; VMH, ventromedial hypothalamus. (TIF) [file pone.0282566.s008.tif]

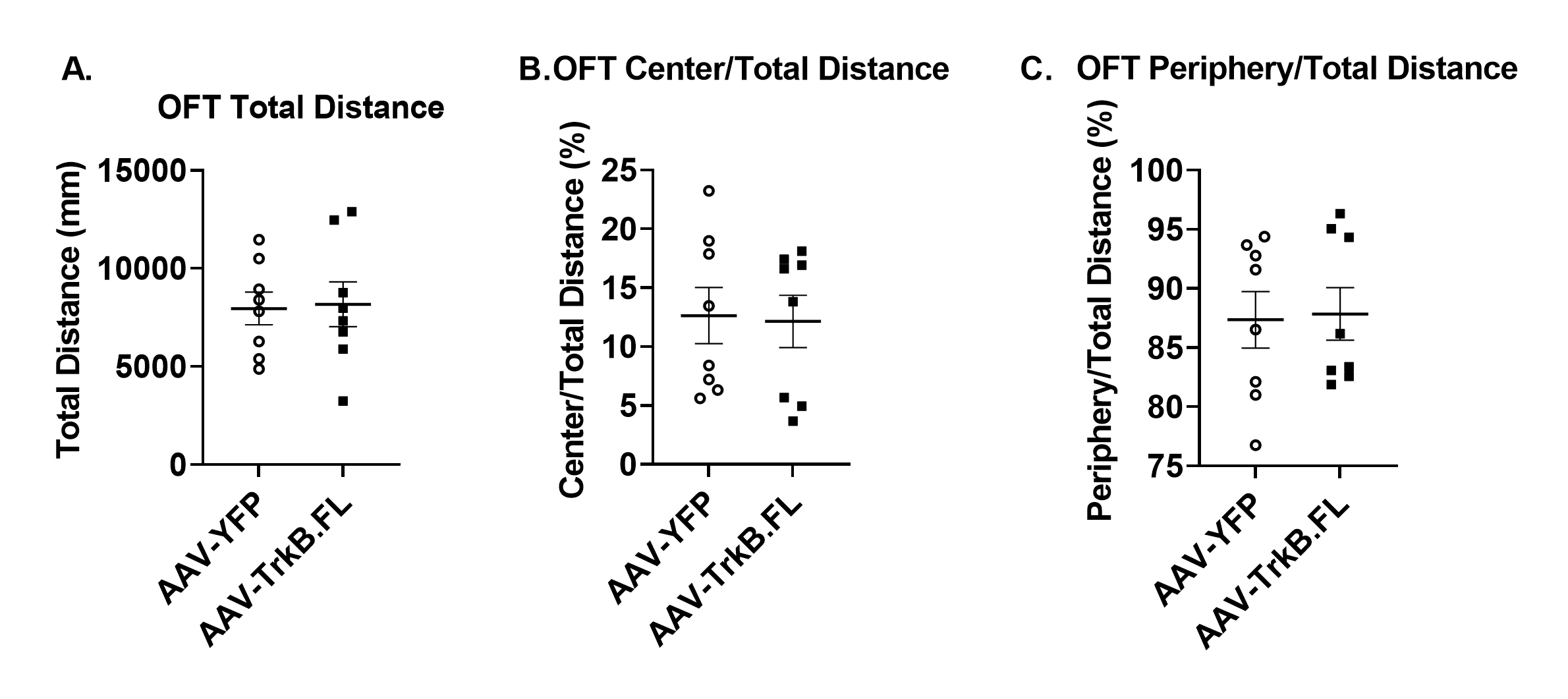

Supplement: S2 Fig — (A) Open field test total distance. (B) Open field test center/total distance ratio. (C) Open field test periphery/total distance ratio. Data are means ±SEM. AAV-YFP: n = 8, AAV-TrkB.FL: n = 8. (TIF) [file pone.0282566.s009.tif]

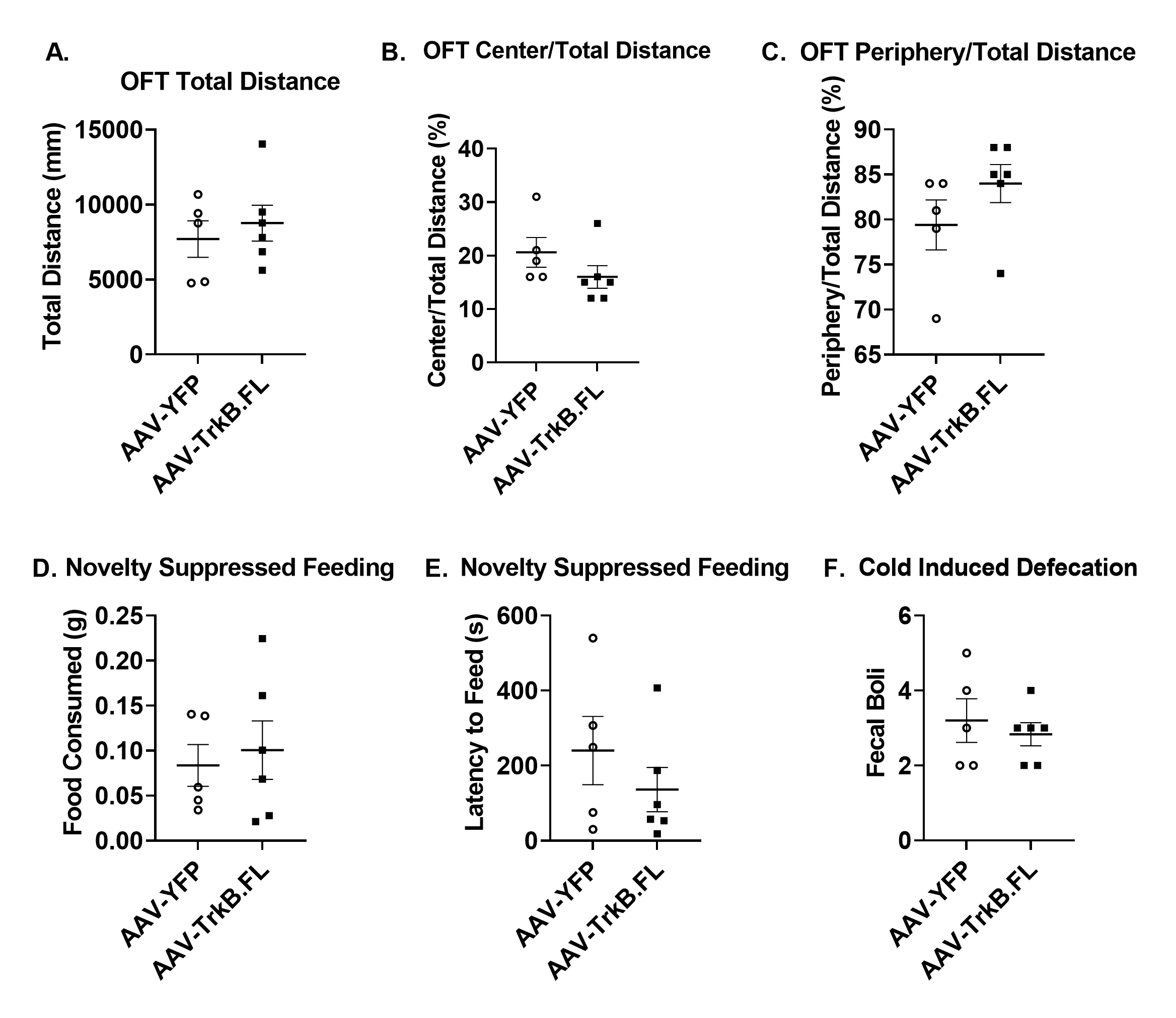

Supplement: S3 Fig — (A) Open field test total distance. (B) Open field test center/total distance ratio. (C) Open field test periphery/total distance ratio. (D) Novelty suppressed feeding food consumed. (E) Novelty suppressed feeding latency to feed. (F) Cold induced defecation fecal boli. Data are means ±SEM. AAV-YFP: n = 5, AAV-TrkB.FL: n = 6. (TIF) [file pone.0282566.s010.tif]

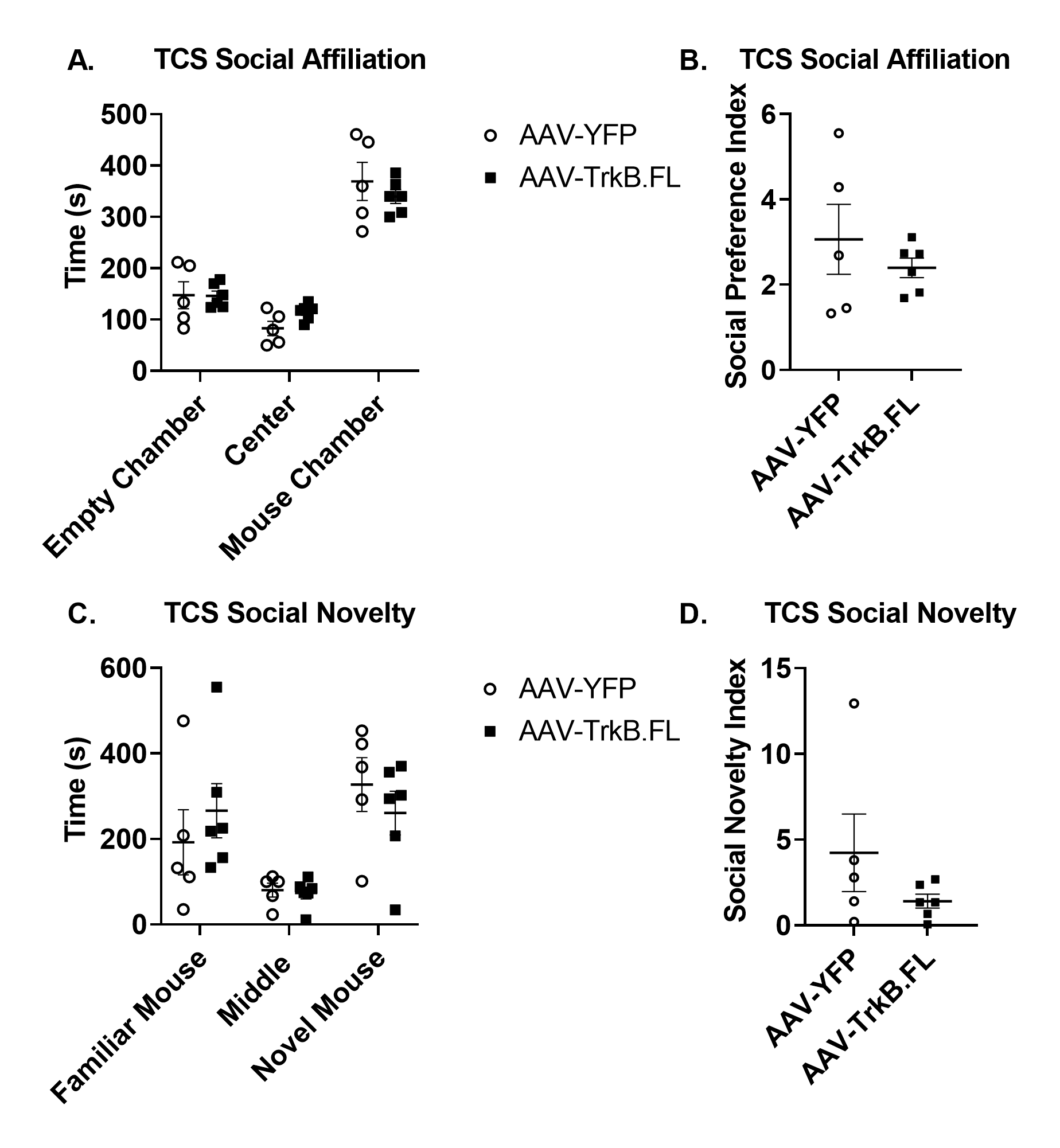

Supplement: S4 Fig — (A) Three chamber sociability social affiliation. (B) Three chamber test sociability social affiliation index. (C) Three chamber sociability test social novelty. (D) Three chamber sociability test social novelty index. Data are means ±SEM. AAV-YFP: n = 5, AAV-TrkB.FL: n = 6. (TIF) [file pone.0282566.s011.tif]

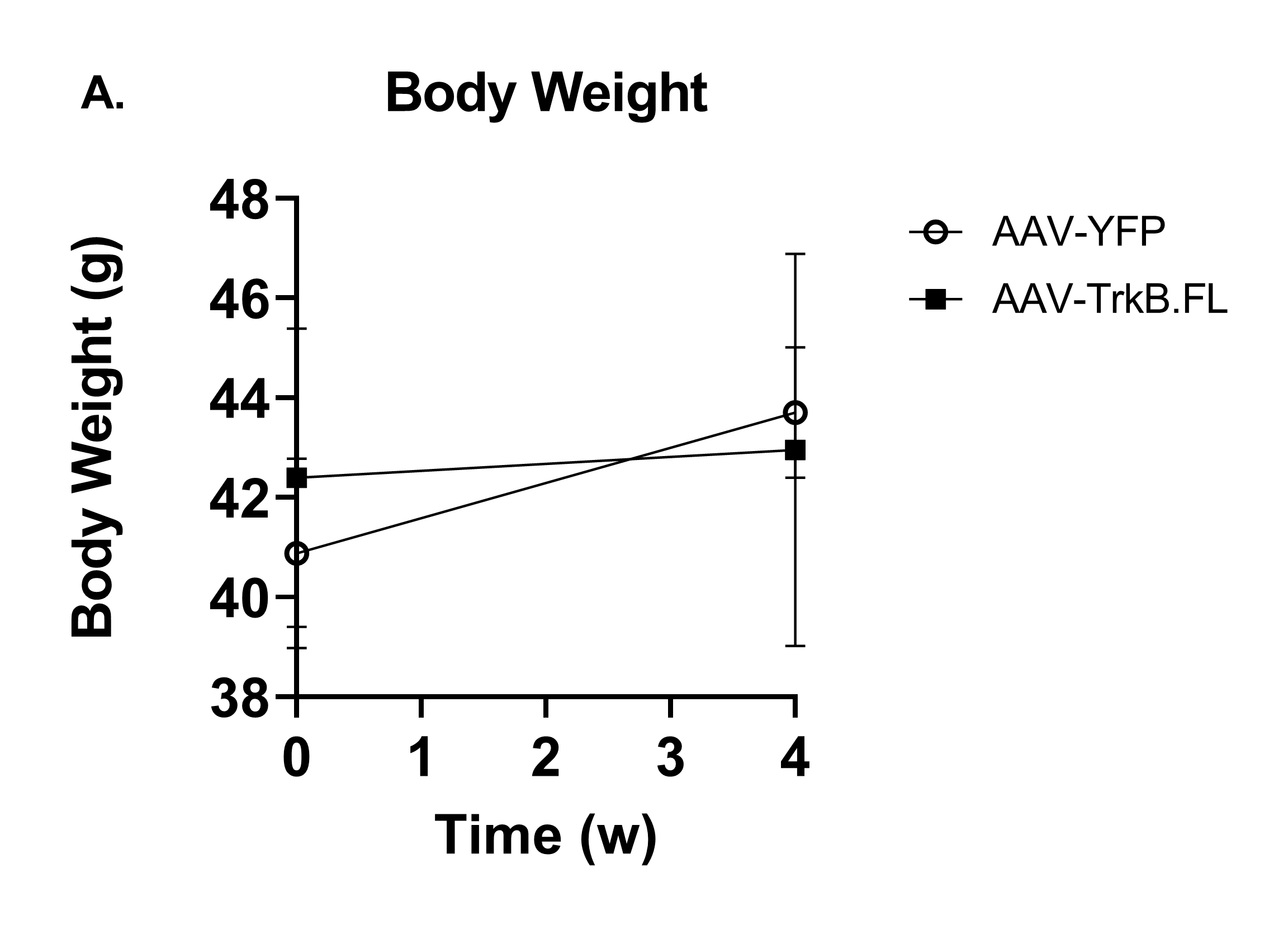

Supplement: S5 Fig — (A) Body weight. Data are means ±SEM. AAV-YFP: n = 5, AAV-TrkB.FL: n = 4. (TIF) [file pone.0282566.s012.tif]

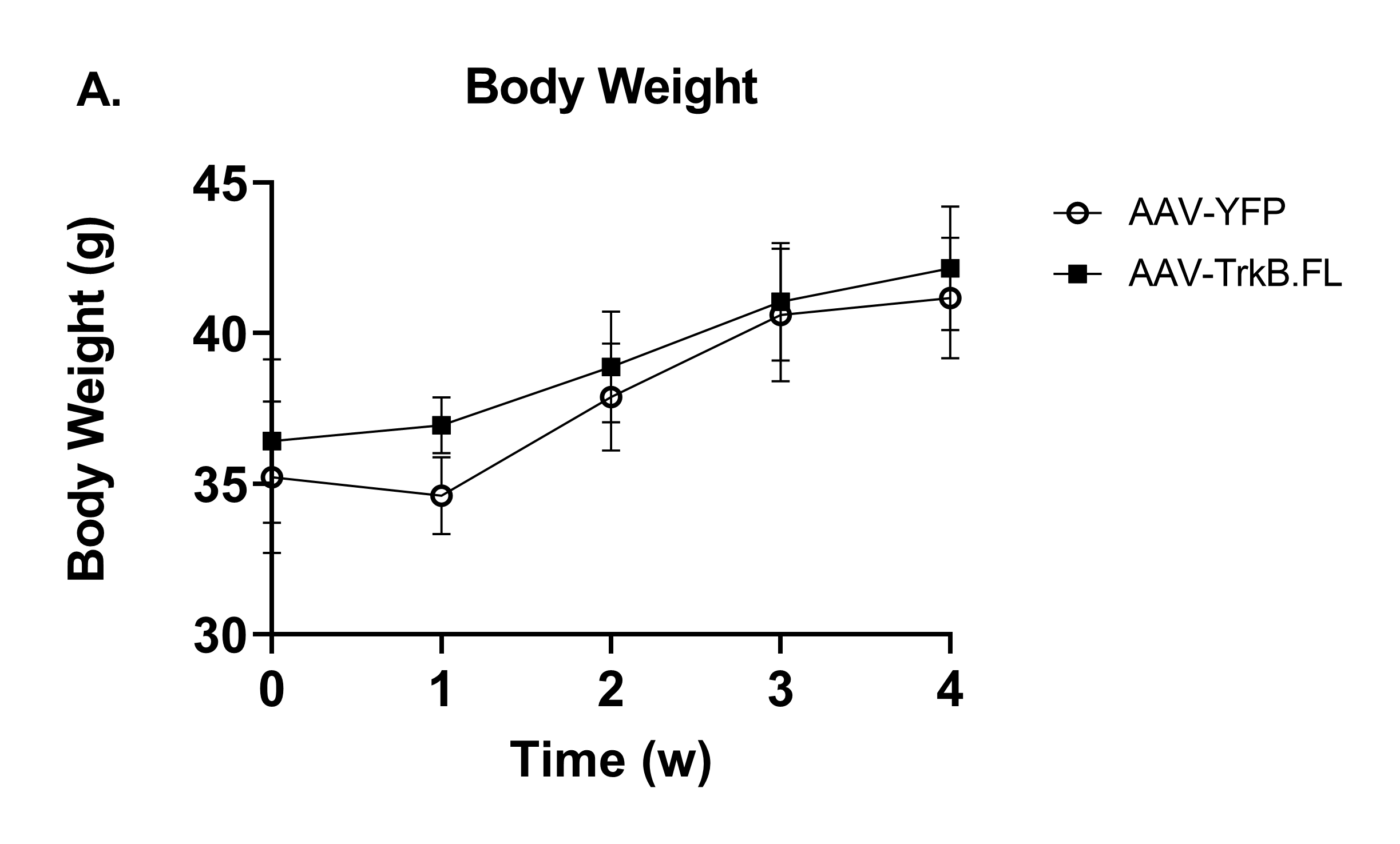

Supplement: S6 Fig — (A) Body weight. Data are means ±SEM. AAV-YFP: n = 4, AAV-TrkB.FL: n = 5. (TIF) [file pone.0282566.s013.tif]

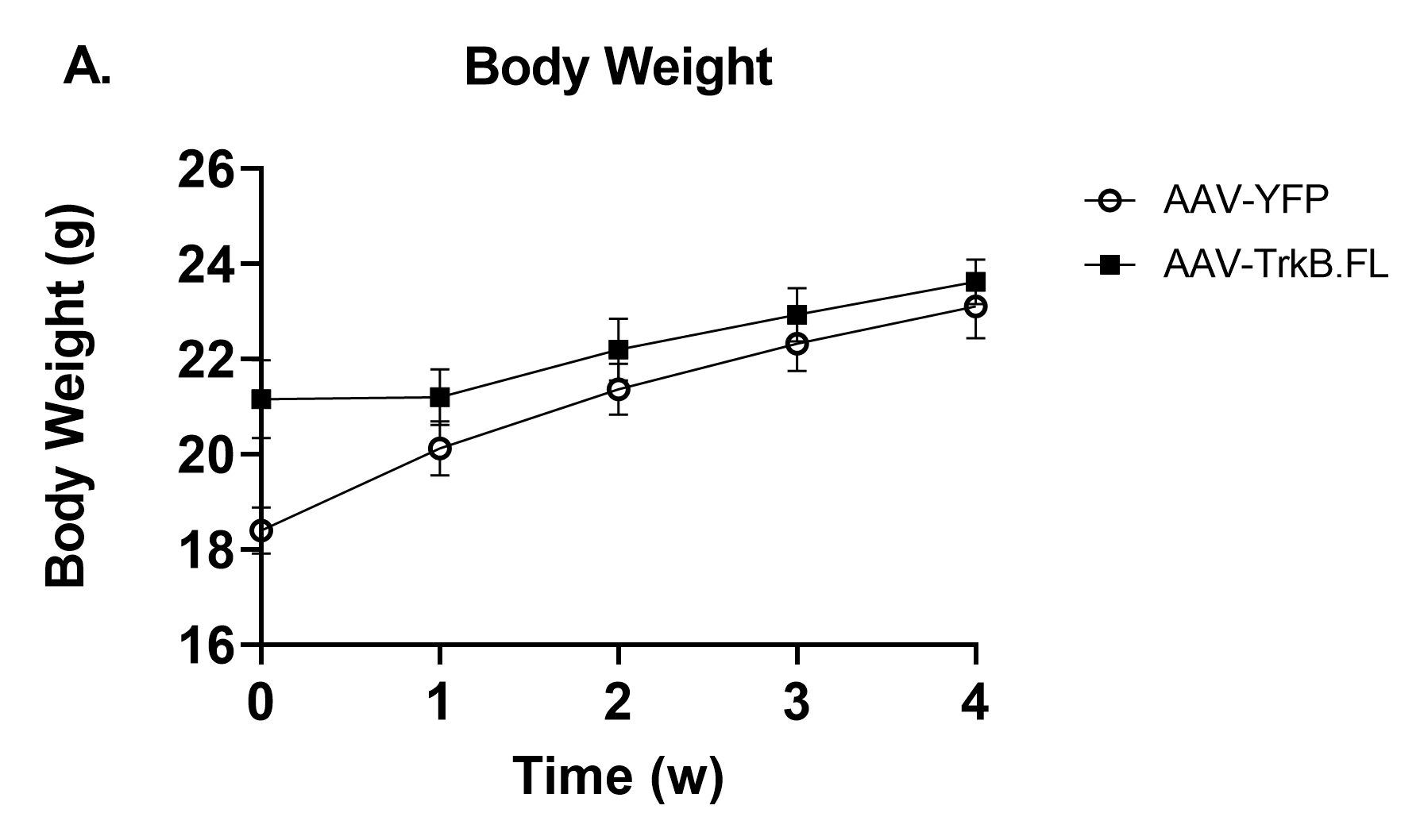

Supplement: S7 Fig — (A) Body weight. Data are means ±SEM. AAV-YFP: n = 5, AAV-TrkB.FL: n = 5. (TIF) [file pone.0282566.s014.tif]
